# Fe(III)-Catalyzed Bicyclization of Yne-Allenones With Indoles for the Atom-Economic Synthesis of 3-Indolyl Cyclobutarenes

**DOI:** 10.3389/fchem.2018.00599

**Published:** 2018-12-04

**Authors:** Heng Li, Wen-Juan Hao, Guigen Li, Shu-Jiang Tu, Bo Jiang

**Affiliations:** ^1^School of Chemistry and Materials Science, Jiangsu Key Laboratory of Green Synthetic Chemistry for Functional Materials, Jiangsu Normal University, Xuzhou, China; ^2^Institute of Chemistry and BioMedical Sciences, Collaborative Innovation Center of Chemistry for Life Sciences, Nanjing University, Nanjing, China; ^3^Department of Chemistry and Biochemistry, Texas Tech University, Lubbock, TX, United States

**Keywords:** Fe(III)-catalysis, bicyclization, 1,6-addition, yne-allenones, cyclobutarenes

## Abstract

A new Fe(III)-catalyzed bicyclization reaction of yne-allenones with indoles has been established, enabling the direct construction of cyclobuta[*a*]naphthalen-4-ols with an all-carbon quaternary center in good to excellent yields. This reaction was performed by using low-cost FeCl_3_ as the catalyst and EtOH as the environmentally benign solvent, providing a green protocol for constructing the cyclobutarene framework with a high degree of atom economy and functional group compatibility. The reaction mechanism was proposed to proceed through a [2 + 2] cycloaddition/1,6-conjugate addition cascade.

## Introduction

Development of practical and sustainable synthetic methods for the rapid construction of valuable cyclic target molecules, along with minimum environmental impacts, represents an endeavor of utmost importance in both academia, and industry (Anastas and Warner, 1998; Bruckmann et al., 2008; Martins et al., 2009; Jiang et al., 2010; Huang et al., 2018a). In this context, chemical transformations following the principles of atom-economy are generally believed to be green since such reactions enable different molecular fragments into integrated cyclic frameworks by recombining chemical bonds with maximum atom utilization and minimum generation of the chemical waste (Trost, 1995, 2002; Trost et al., 2003; Banert and Plefka, 2011; Kotha et al., 2013). The key to realize this goal is to implement reaction cascades, which allow the direct formation of multiple chemical bonds in a one-pot operation and can lead to a remarkable increase in resource efficiency for the overall process (Barluenga et al., 2009; Fuerstner, 2009; Tietze et al., 2009; Jones et al., 2010; Wang et al., 2015; Sugimoto and Matsuya, 2017; Zhang et al., 2017). Specifically, bicyclization cascades have emerged as an important platform for the synthesis of bioactive small-molecule libraries for their SAR studies (Dömling et al., 2012; Brauch et al., 2013; Vlaar et al., 2013; Koopmanschap et al., 2014; Rotstein et al., 2014; Huang et al., 2018b). Due to their annulation efficiency, economic and environmental aspects, and ease of operation as well as diminished waste disposals (Jia et al., 2014; Su et al., 2014; Tian et al., 2015; Chen et al., 2017; Huang et al., 2017; Liu et al., 2017b,c; Wang L. et al., 2017). In view of the environmental awareness of the chemical community, the combination of the presented bicyclization strategy and the use of environmentally benign solvents will furnish the transformations under avoidance of potential pollutants (Bihani et al., 2013; Wang J.-Y. et al., 2017; Sha et al., 2018a). Nevertheless, the design and development of environmentally compatible bicyclization cascades without generation of toxic waste and by-products holds considerable challenges.

**Scheme 1:**
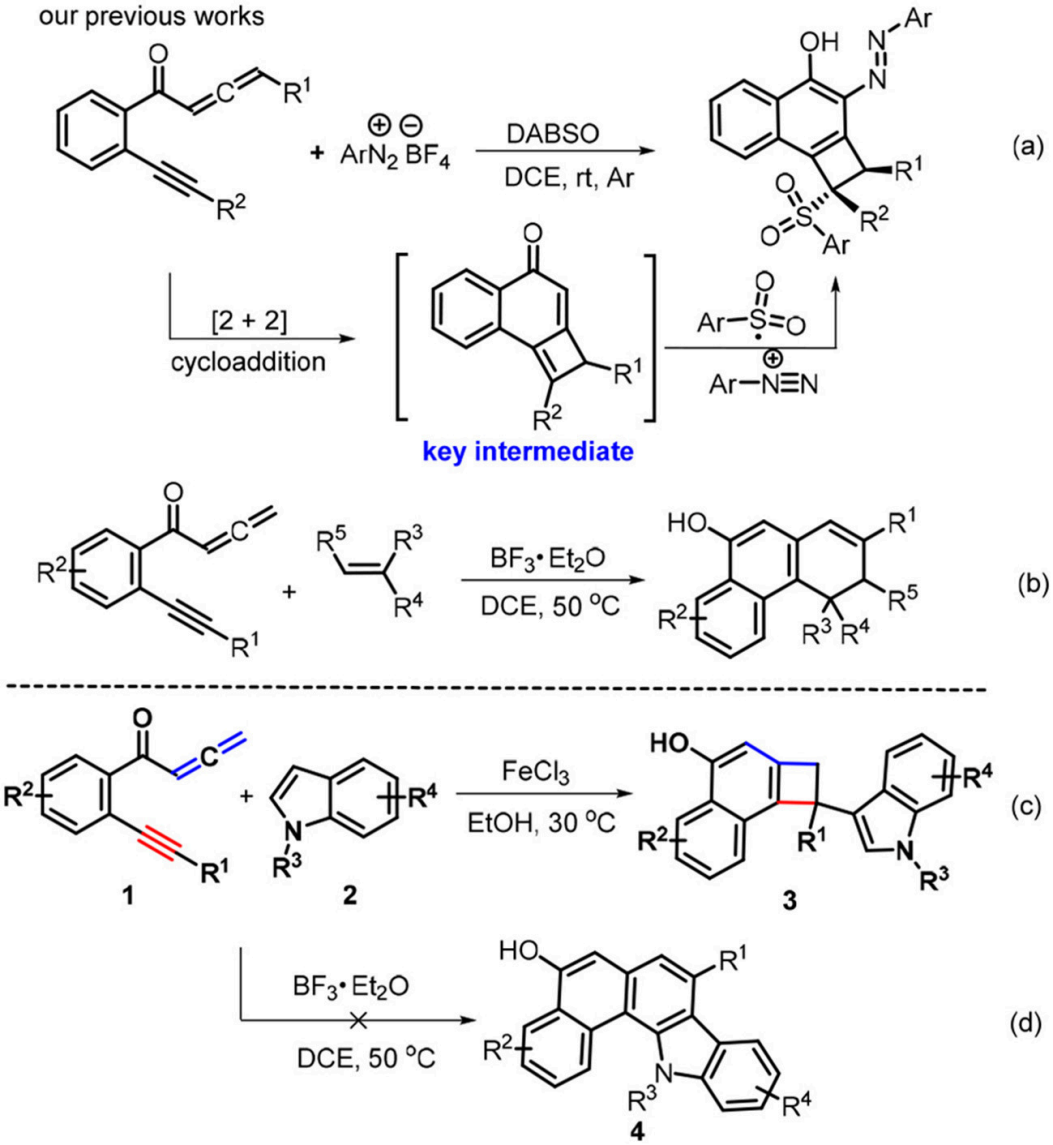
Profiles for [2 + 2] cycloaddition of yne-allenones.

Cyclobutarenes are a class of structurally unique bicarbocyclic molecules which show a wide spectrum of biological activity (Christophe et al., 1998; Sadana et al., 2003). Due to the thermodynamic stability associated with the aromatic system and the kinetic reactivity of the strained cyclobutene ring (Cava and Napier, 1956; Mehta and Kotha, 2001), these molecules behave as reliable and synthetically useful feedstocks (Christophe et al., 1998) and have been extensively applied in natural product syntheses (Funk and Vollhardt, 1977, 1979; Grieco et al., 1980; Taber et al., 1987; Nemoto et al., 1995; Michellys et al., 2001). With these contributions in mind, great efforts to establish synthetic protocols for cyclobutarene synthesis have been developed which include 1,4-elimination-cycloaddition of functionalized arenes (Gray et al., 1978; Schirch et al., 1979; Sekine et al., 1979; Lenihan and Shechter, 1994, 1998; Chou et al., 1995), Parham cyclization (Bradsher and Hunt, 1981; Buchwald et al., 1987a,b; Beak and Selling, 1989; Aidhen and Ahuja, 1992), photo-induced cycloadditions (Parham et al., 1976; Kaneko and Naito, 1979; Neckers and Wagenaar, 1981; Kaneko et al., 1982; Kanao et al., 1983; Sato et al., 1987; Hoffmann and Pete, 1996), thermal extrusion reactions (Toda et al., 1988; D'Andrea et al., 1990; Hickman et al., 1991; Shimada et al., 1993; Andersen et al., 1996; Craig et al., 1998), intramolecular addition of carbanions to benzynes (Bunnett and Skorcz, 1962; Krohn et al., 1978; Gowland and Durst, 1979), [2 + 2 + 2] cycloadditions of 1,5-hexadiyne (Peter and Vollhardt, 1977, 1984; Funk and Vollhardt, 1980; McNichols and Stang, 1992), ring expansion of cycloproparenes (Birch et al., 1964; Iskander and Stansfield, 1965; Buckland et al., 1987; Kagabu and Saito, 1988; Müller et al., 1989), and [2 + 2] cycloadditions of allene precursors (Inanaga et al., 1992; Ezcurra and Moore, 1993; Toda et al., 1994) and other methods (Markgraf et al., 1969; Garratt and Nicolaides, 1972, 1974; Bilyard et al., 1979; Warrener et al., 1993). However, these methods encounter some drawbacks such as high temperatures (400°C-800°C), strong bases (*n*-BuLi and NaNH_2_), multiple steps, or a narrow substrate range. Moreover, indole derivatives stand for another important class of heterocyclic compounds present in a myriad of bioactive substances and natural products. Therefore, the development of general and sustainable entries toward cyclobutarene-indole pairs in atom- and pot-economic manner is of potential significance. Recently, we reported the combination of [2 + 2] cycloaddition with 1,4-radical addition reaction by treating yne-allenones with aryldiazonium salts and DABCO-bis(sulfur dioxide) (DABSO), affording functional cyclobuta[*a*]naphthalen-4-ols (Scheme 1a, Liu et al., 2017a). Subsequently, we developed a BF_3_•Et_2_O-catalyzed double [2 + 2] cycloaddition relay between yne-allenones and unactivated alkenes, enabling C-C triple bond cleavage to access phenanthren-9-ols (Scheme 1b, Li et al., 2018a). To continue our efforts in this project (Liu et al., 2017a; Wang J.-Y. et al., 2017; Li et al., 2018a,b; Sha et al., 2018b; Wang et al., 2018), we attempted to employ indoles **2** to be subjected with the reaction of yne-allenones **3** under our previous conditions (Li et al., 2018a) to assemble naphtho[1,2-*a*]carbazol-5-ols **4** (Scheme 1d), owing to indoles with C2 and C3 reactive sites could acted as C_2_ synthons for the synthesis of fused indoles (Haibach et al., 2011; Li et al., 2015; Liu et al., 2016; Ozaki et al., 2017). Unexpectedly, a double [2 + 2] cycloaddition relay did not occur. Instead, the reaction involved another [2 + 2] cycloaddition /1,6-addtition cascade to furnish 3-indolyl substituted cyclobuta[*a*]naphthalen-4-ols **3** by suitably adjusting the catalysts and solvents (Scheme 1c). Notably, the current green protocol represents an atom-economic and eco-friendly entry to structurally unique cyclobutarene-indole pairs through the combination of [2 + 2] cycloaddition with 1,6-conjugate addition by FeCl_3_ as a low-cost catalyst and EtOH as an environmentally benign solvent. Herein, we elaborate this attractive and benign transformation.

## Results and Discussion

At the beginning of our studies, yne-allenone **1a** and *N*-methylindole (**2a**) were chosen as the model substrates to explore the feasibility of double [2 + 2] cycloaddition relay reaction with our previous conditions (Table 1, entry 1). Instead of the expected naphtho[1,2-*a*]carbazol-5-ol **4a**, 3-indolyl substituted cyclobuta[*a*]naphthalen-4-ol **3a** was obtained in 60% yield. The following screening of solvents, such as *N*,*N*-dimethylformamide (DMF), 1,4-dioxane, tetrahydrofuran (THF), MeOH, and EtOH, showed that use of DMF and 1,4-dioxane as reaction media completely suppressed the reaction process (entries 2–3) whereas the latter three all made the transformations work more efficiently (entries 4–6). Among these, EtOH proved to be the best choice, providing the product **3a** with the highest yield of 76% (entry 6). Increasing the component ratio to 1:2 is not beneficial for this transformation as a lower conversion was observed (63%, entry 7). In contrast, fine-tuning the component ratio to 1:1.2 could improve the reaction efficiency, resulting in a higher yield of **3a** (81%, entry 8). As the next optimization step, we conducted the screening of a variety of Lewis acid catalysts, such as ZnCl_2_, Y(OTf)_3_ and FeCl_3_ that are often employed in the catalytic transformations, for this cyclization-addition cascade by using EtOH as the reaction media. The former two led to remarkably lower conversions (entries 9–10). Delightingly, the latter one showed the best catalytic performance in this transformation, delivering higher yield of **3a** as compared with BF_3_•Et_2_O (85%, entry 11 vs. 8). It is found that the reaction efficiency was proven to display an important dependence on the loading of the Fe-catalyst. An increase in the FeCl_3_ loading had a detrimental impact on the reaction yield (entry 12) whereas reducing the catalytic amount of FeCl_3_ to 10 mol% could accelerate the conversion into **3a** in an increased the yield to 88%. When the reaction temperature was elevated to 70°C, the reaction process was inhibited in some extent (entry 14). On the contrary, decreasing the reaction temperature to 30°C facilitated the current transformation and gave a higher yield of 90% (entry 15).

**Table 1:** Optimization of Reaction Conditions^[a]^.

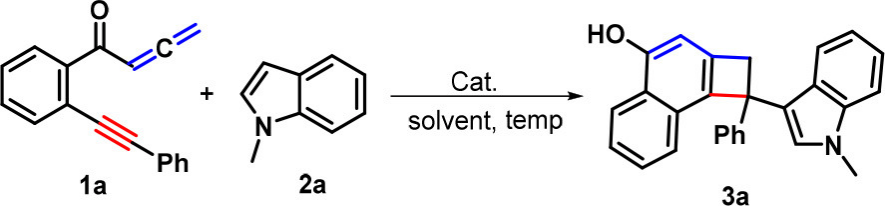					
**Entry**	**Ratio (1a:2a)**	**Cat. (mol%)**	**Solvent**	**Temp (°C)**	**Yield (%)**^[b]^
1	1:1.5	BF_3_•Et_2_O (15)	DCE	50	60
2	1:1.5	BF_3_•Et_2_O (15)	DMF	50	N.D.
3	1:1.5	BF_3_•Et_2_O (15)	1,4-dioxane	50	N.D.
4	1:1.5	BF_3_•Et_2_O (15)	THF	50	66
5	1:1.5	BF_3_•Et_2_O (15)	MeOH	50	62
6	1:1.5	BF_3_•Et_2_O (15)	EtOH	50	76
7	1:2	BF_3_•Et_2_O (15)	EtOH	50	63
8	1:1.2	BF_3_•Et_2_O (15)	EtOH	50	81
9	1:1.2	ZnCl_2_ (15)	EtOH	50	60
10	1:1.2	Y(OTf)_3_ (15)	EtOH	50	25
11	1:1.2	FeCl_3_ (15)	EtOH	50	85
12	1:1.2	FeCl_3_ (20)	EtOH	50	74
13	1:1.2	FeCl_3_ (10)	EtOH	50	88
14	1:1.2	FeCl_3_ (10)	EtOH	70	72
15	1:1.2	FeCl_3_ (10)	EtOH	30	90

[a] Reaction conditions: Benzene-tethered yne-allenone (**1a**, 0.1 mmol, 1.0 equiv), N-methylindole (**2a**, x mmol), catalyst (y mol%), solvent (5.0 mL), air, 8 h

[b] *Isolated yield based on **1a***.

**Scheme 2:**
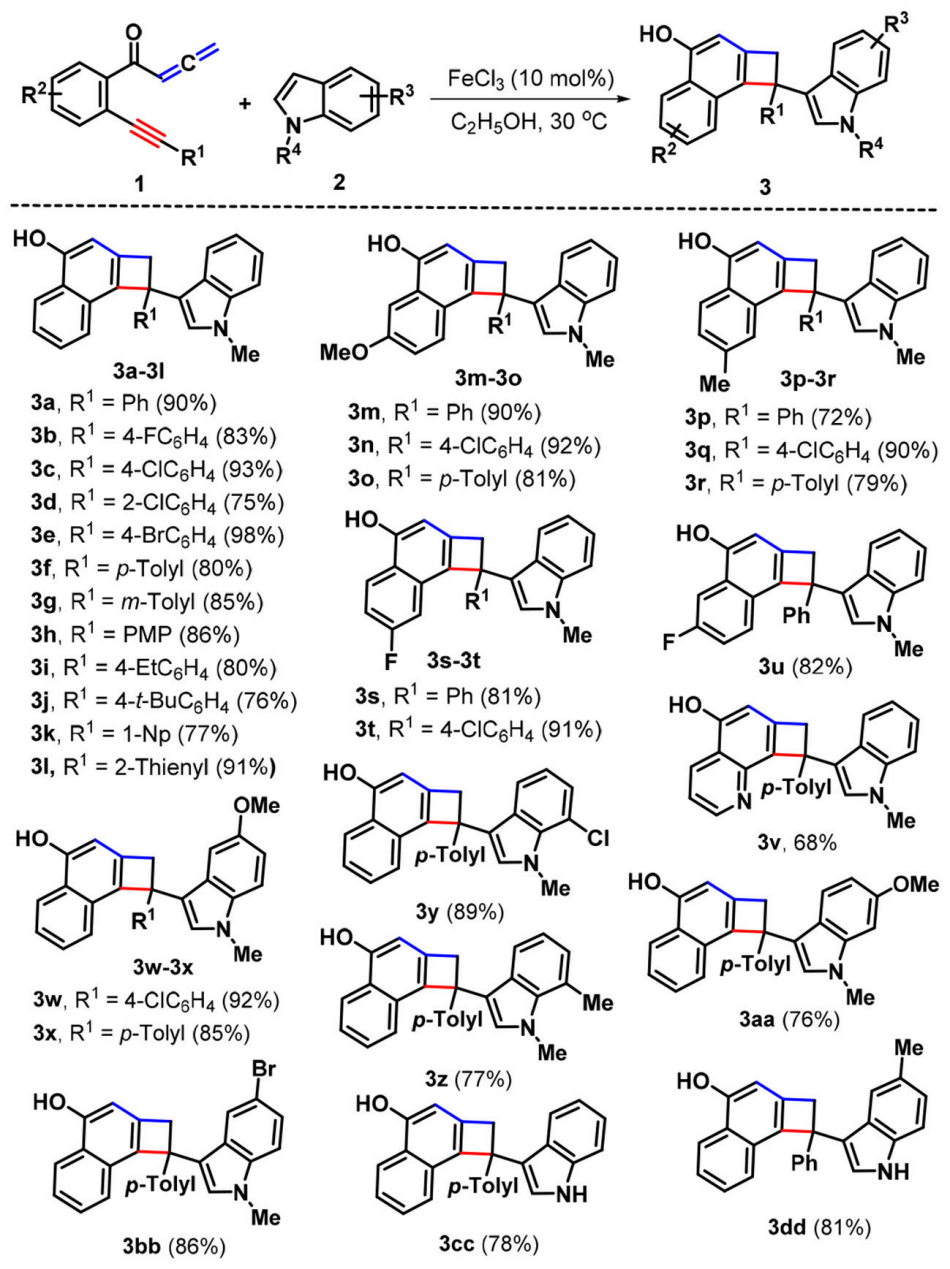
Substrate scope for products 3.

With these optimal conditions in hand (Table 1, entry 15), we set out to examine the scope of this Fe-catalyzed [2 + 2] cycloaddition /1,6-addition cascade by using a variety of yne-allenones and indoles. As depicted in Scheme 2, *N*-methylindole (**2a**) was first selected to evaluate the influence of substituents (R^1^) in the arylalkynyl moiety of yne-allenone **1**. Both electron-poor and electron-rich groups at different positions of the arylalkynyl moiety (R^1^) can all tolerate this catalytic system, efficiently accessing the corresponding products **3b-3j** in 75–98% yields. Diverse substituents, such as fluoro (**1b**), chloro (**1c** and **1d**), bromo (**1e**), methyl (**1f** and **1g**), methoxy (PMP = *p*-methoxyphenyl, **1h**), ethyl(**1i**), *t*-butyl (**1j**) were suitable for this transformation. The presence of soft electron-withdrawing substituents (chloro, **1c** and bromo, **1e**) at the *para*-positions seemed to result in higher reactivity than that of electron-donating counterparts (**3c** and **3e** vs. **3f** and **3h-3j**). Moreover, a sterically encumbered 1-naphthyl (1-Np) analog **1k** was an effective candidate, which proceeded through a similar cyclization-addition process to give the corresponding product **3k** in 77% yield. Besides, 2-thienyl counterpart **1l** still showed high reactivity, delivering 2-thienyl product **3l** in 91% yield. Next, we placed different functional groups (R^2^) including methoxy, methyl, and fluoro into the C4 or C5 position of the internal arene ring of substrates **1** and explored the synthetic utility of these substrates. Satisfyingly, all those substituents (**1m**−**1u**) would be compatible in the present reaction protocol, and the corresponding functionalized cyclobuta[a]naphthalen-4-ols **3m-3u** in 72–92% yields were produced. Interestingly, the pyridine-tethered yne-allenone **1v** could be successfully converted into cyclobutarene product **3v** in 68% yield.

**Figure 1:**
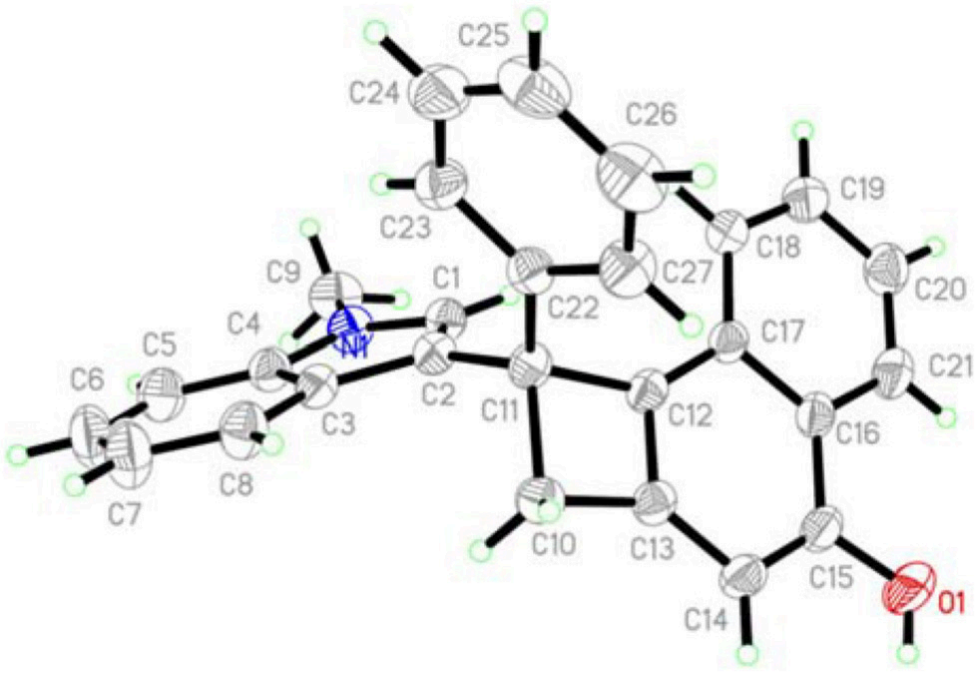
X-Ray structure of product **3a**.

Next, the scope with respect to indoles components was evaluated. As anticipated, the different substituents including methoxy (**2b**), chloro (**1c** and **1d**), bromo (**1e**), methyl (**1f** and **1g**), located at different positions of the indole ring would be accommodated, confirming the reaction efficiency, as the cyclobuta[a]naphthalen-4-ol products **3w-3bb** were offered in 76–92% yields. Finally, the free indole turned out to be a suitable reaction partner, leading to the formation of products **3cc** and **3dd** in 78 and 81% yields, respectively. Products **3** were fully characterized by their NMR and HR-MS spectral analysis. In the case of product **3a**, its structure was further confirmed by X-ray crystallography (Figure 1).

## Mechanism

**Scheme 3:**
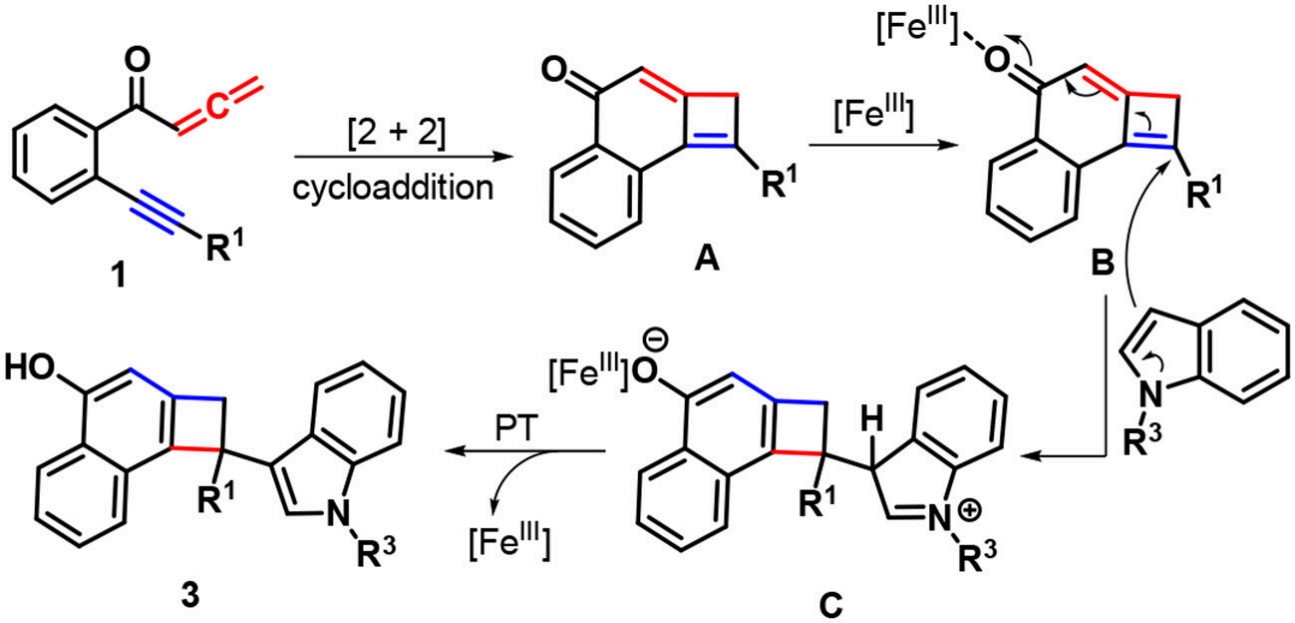
Plausible reaction pathway.

Based on the above experimental observations and literature reports (Haibach et al., 2011; Li et al., 2015, 2018a; Liu et al., 2016, 2017a; Ozaki et al., 2017; Sha et al., 2018b), a feasible mechanism for forming products **3** was proposed in Scheme 3. Initially, the intramolecular [2 + 2] cycloaddition of yne-allenones **1** rapidly occurs to yield cyclobutene intermediate **A**. In the presence of Fe-catalyst, 1,6-addition of indoles into intermediate **B** gives intermediate **C**, which converts into the final products **3** through proton transfer (PT), together with the regeneration of Fe-catalyst.

## Conclusion

In summary, starting from readily available yne-allenones and indoles, we have established a new Fe-catalyzed [2 + 2] cycloaddition/1,6-conjugate addition cascade for the high-efficient and benign synthesis of a variety of 3-indolyl cyclobuta[*a*]naphthalen-4-ols with good to excellent yields. The current green protocol has the advantages of broad scope of substrates, good tolerance of functional group and high atom utilization as well as mild reaction conditions. Further application of the resulting cyclobutarenes is underway in our laboratory.

## Materials and Methods

### General

All melting points are uncorrected. The NMR spectra were recorded in CDCl_3_ or DMSO-*d*_6_ on a 400 MHz instrument with TMS as internal standard. Chemical shifts (δ) were reported in ppm with respect to TMS. Data are represented as follows: chemical shift, mutiplicity (s = singlet, d = doublet, t = triplet, m = multiplet), coupling constant (J, Hz) and integration. HRMS analyses were carried out using a TOF-MS instrument with an ESI source. X-Ray crystallographic analysis was performed with a SMART CCD and a P4 diffractometer (the copies of NMR see Supplementary Material). All yne-allenones **1** are known compounds and their preparation followed the previously reported procedures (Wei et al., 2009; Liu et al., 2017a; Li et al., 2018b).

### General Procedure for the Synthesis of 3

#### Example for the Synthesis of 3a

1-(2-(Phenylethynyl)phenyl)buta-2,3-dien-1-one (**1a**, 0.3 mmol, 73.2 mg) was added to a 10-mL reaction tubing under the air conditions. Then, *N*-methylindole (**2a**, 0.36 mmol, 47.2 mg) and EtOH (5 mL) were continuously added into the above reaction mixture. Subsequently, FeCl_3_ (10 mol%, 4.8 mg) was added to the reaction system. Then the mixture was stirred at 30°C for 8 h until complete consumption of **1a** as monitored by TLC analysis. After the reaction was finished, the reaction mixture was concentrated in vacuum, and the resulting residue was purified by column chromatography on silica gel (eluent, petroleum ether/ethyl acetate = 20:1) to afford the desired product **3a** as a white solid.

#### 1-(1-methyl-1H-indol-3-yl)-1-phenyl-1,2-dihydrocyclobuta[a]naphthalen-4-ol (3a)

White solid, 102 mg, 90% yield; mp 179-181°C; ^1^H NMR (400 MHz, DMSO-*d*_6_; δ, ppm) 10.23 (s, 1H), 8.24 (d, *J* = 8.0 Hz, 1H), 7.51 (d, *J* = 7.6 Hz, 1H), 7.38 (m, 5H), 7.29 (m, 2H), 7.21 (m, 1H), 7.07 (m, 1H), 6.91-6.78 (m, 4H), 3.88 (m, 2H), 3.68 (s, 3H); ^13^C NMR (100 MHz, DMSO-*d*_6_; δ, ppm) 154.8, 146.1, 140.3, 137.7, 137.0, 130.1, 128.5, 128.1, 127.5, 127.4, 126.8, 126.5, 125.2, 124.4, 123.9, 122.4, 121.5, 120.1, 119.0, 118.8, 110.3, 104.9, 54.4, 47.7, 32.7; IR (KBr, ν, cm^−1^) 3341, 3044, 1579, 1474, 1228, 1181, 1024, 905, 806, 738; HRMS (APCI-TOF) m/z calcd for C_27_H_20_NO [M-H]^−^ 374.1545; found 374.1546.

#### 1-(4-fluorophenyl)-1-(1-methyl-1H-indol-3-yl)-1,2-dihydrocyclobuta[a]naphthalen-4-ol (3b)

White solid, 97 mg, 83% yield; mp 160-162°C; ^1^H NMR (400 MHz, DMSO-*d*_6_; δ, ppm) 10.24 (s, 1H), 8.25 (d, *J* = 8.0 Hz, 1H), 7.49 (d, *J* = 8.0 Hz, 1H), 7.43-7.34 (m, 5H), 7.11 (m, 3H), 6.87 (m, 4H), 3.88 (m, 2H), 3.68 (s, 3H); ^13^C NMR (100 MHz, DMSO-*d*_6_; δ, ppm) 162.2 (^1^*J*_CF_ = 240.5 Hz), 159.8, 154.9, 142.3 (^4^*J*_CF_ = 2.9 Hz), 142.2, 140.3, 137.7, 136.8, 129.9, 129.4 (^3^*J*_CF_ = 7.9 Hz), 129.3, 128.1, 127.5, 126.7, 125.2, 124.4, 123.9, 122.2, 121.6, 120.0, 119.1, 118.6, 115.3 (^2^*J*_CF_ = 21.0 Hz), 115.1, 110.4, 105.0, 53.7, 47.9, 32.7; IR (KBr, ν, cm^−1^) 3399, 3051, 1573, 1464, 1226, 1188, 1014, 904, 808, 748; HRMS (APCI-TOF) m/z calcd for C_27_H_19_FNO [M-H]^−^ 392.1451; found 392.1453.

#### 1-(4-chlorophenyl)-1-(1-methyl-1H-indol-3-yl)-1,2-dihydrocyclobuta[a]naphthalen-4-ol (3c)

White solid, 114 mg, 93% yield; mp 170-172°C; ^1^H NMR (400 MHz, DMSO-*d*_6_; δ, ppm) 10.27 (s, 1H), 8.24 (d, *J* = 8.0 Hz, 1H), 7.47 (d, *J* = 7.6 Hz, 1H), 7.44-7.31 (m, 7H), 7.12-7.06 (m, 1H), 6.93 (s, 1H), 6.87 (m, 3H), 3.82 (m, 2H), 3.69 (s, 3H); ^13^C NMR (100 MHz, DMSO-*d*_6_; δ, ppm) 154.9, 145.1, 140.3, 137.7, 136.5, 131.0, 129.9, 129.3, 128.5, 128.2, 127.6, 126.7, 125.2, 124.4, 124.0, 122.1, 121.6, 120.0, 119.1, 118.2, 110.4, 104.9, 53.7, 47.8, 32.7; IR (KBr, ν, cm^−1^) 3407, 3031, 1563, 1441, 1223, 1103, 1012, 909, 838, 741; HRMS (APCI-TOF) m/z calcd for C_27_H_19_ClNO [M-H]^−^ 408.1156; found 408.1143.

#### 1-(2-chlorophenyl)-1-(1-methyl-1H-indol-3-yl)-1,2-dihydrocyclobuta[a]naphthalen-4-ol (3d)

White solid, 92 mg, 75% yield; mp 177-179°C; ^1^H NMR (400 MHz, DMSO-*d*_6_; δ, ppm) 10.31 (s, 1H), 8.30-8.19 (m, 1H), 7.65 (d, *J* = 7.6 Hz, 1H), 7.53 (d, *J* = 7.6 Hz, 1H), 7.44-7.28 (m, 6H), 6.99 (m, 1H), 6.93 (s, 1H), 6.79 (s, 1H), 6.69 (m, 1H), 6.60 (d, *J* = 8.0 Hz, 1H), 4.32 (d, *J* = 14.4 Hz, 1H), 3.71 (s, 1H), 3.66 (s, 3H); ^13^C NMR (100 MHz, DMSO-*d*_6_; δ, ppm) 155.2, 143.4, 140.3, 137.7, 135.9, 134.0, 131.0, 130.5, 130.1, 128.9, 127.9, 127.6, 127.4, 126.2, 125.2, 124.4, 123.9, 122.2, 121.1, 119.3, 118.9, 116.1, 110.2, 104.7, 55.3, 47.5, 32.7; IR (KBr, ν, cm^−1^) 3442, 3022, 1533, 1421, 1203, 1123, 1010, 903, 834, 721; HRMS (APCI-TOF) m/z calcd for C_27_H_19_ClNO [M-H]^−^ 408.1156; found 408.1162.

#### 1-(4-bromophenyl)-1-(1-methyl-1H-indol-3-yl)-1,2-dihydrocyclobuta[a]naphthalen-4-ol (3e)

White solid, 133 mg, 98% yield; mp 166-168°C; ^1^H NMR (400 MHz, DMSO-*d*_6_; δ, ppm) 10.26 (s, 1H), 8.24 (d, *J* = 8.0 Hz, 1H), 7.48 (d, *J* = 8.4 Hz, 3H), 7.43-7.34 (m, 3H), 7.30 (d, *J* = 8.4 Hz, 2H), 7.09 (m, 1H), 6.93 (s, 1H), 6.92-6.83 (m, 3H), 3.81 (m, 2H), 3.69 (s, 3H). ^13^C NMR (100 MHz, DMSO-*d*_6_; δ, ppm) 154.9, 145.5, 140.3, 137.7, 136.5, 131.5, 129.9, 129.7, 128.2, 127.6, 126.7, 125.3, 124.4, 124.0, 122.1, 121.6, 120.0, 119.6, 119.1, 118.1, 110.4, 104.9, 53.8, 47.8, 32.7. IR (KBr, ν, cm^−1^) 3412, 3021, 1538, 1391, 1202, 1129, 1017, 906, 818, 733; HRMS (APCI-TOF) m/z calcd for C_27_H_19_BrNO [M-H]^−^ 452.0651; found 452.0634.

#### 1-(1-methyl-1H-indol-3-yl)-1-(p-tolyl)-1,2-dihydrocyclobuta[a]naphthalen-4-ol (3f)

White solid, 94 mg, 80% yield; mp 155-157°C; ^1^H NMR (400 MHz, DMSO-*d*_6_; δ, ppm) 10.20 (s, 1H), 8.23 (d, *J* = 8.0 Hz, 1H), 7.49 (d, *J* = 8.0 Hz, 1H), 7.42-7.34 (m, 3H), 7.26 (d, *J* = 8.0 Hz, 2H), 7.08 (d, *J* = 8.0 Hz, 3H), 6.92-6.79 (m, 4H), 3.80 (m, 2H), 3.68 (s, 3H), 2.26 (s, 3H); ^13^C NMR (100 MHz, DMSO-*d*_6_; δ, ppm) 154.7, 143.0, 140.3, 137.7, 137.2, 135.3, 130.0, 129.1, 128.0, 127.4, 127.3, 126.8, 125.2, 124.3, 123.8, 122.4, 121.5, 120.2, 119.0, 118.9, 110.3, 105.0, 54.0, 47.8, 32.7, 21.1; IR (KBr, ν, cm^−1^) 3418, 3009, 1541, 1401, 1192, 1121, 1012, 916, 813, 730; HRMS (APCI-TOF) m/z calcd for C_28_H_22_NO [M-H]^−^ 388.1702; found 388.1723.

#### 1-(1-methyl-1H-indol-3-yl)-1-(m-tolyl)-1,2-dihydrocyclobuta[a]naphthalen-4-ol (3g)

White solid, 99 mg, 85% yield; mp 149-151°C; ^1^H NMR (400 MHz, DMSO-*d*_6_; δ, ppm) 10.20 (s, 1H), 8.23 (d, *J* = 8.4 Hz, 1H), 7.51 (d, *J* = 8.0 Hz, 1H), 7.38 (m, 3H), 7.23 (s, 1H), 7.18 (d, *J* = 5.6 Hz, 2H), 7.09-7.01 (m, 2H), 6.85 (m, 4H), 3.82 (m, 2H), 3.68 (s, 3H), 2.22 (s, 3H); ^13^C NMR (100 MHz, DMSO-*d*_6_; δ, ppm) 154.7, 146.0, 140.3, 137.7, 137.4, 137.1, 128.4, 128.0, 127.4, 127.2, 126.8, 125.2, 124.8, 124.3, 123.8, 120.1, 118.9, 110.3, 105.0, 54.3, 47.7, 32.7, 21.8; IR (KBr, ν, cm^−1^) 3411, 2966, 1511, 1406, 1162, 1091, 1015, 911, 833, 727; HRMS (APCI-TOF) m/z calcd for C_28_H_22_NO [M-H]^−^ 388.1702; found 388.1720.

#### 1-(4-methoxyphenyl)-1-(1-methyl-1H-indol-3-yl)-1,2-dihydrocyclobuta[a]naphthalen-4-ol (3h)

White solid, 105 mg, 86% yield; mp 167-169°C; ^1^H NMR (400 MHz, DMSO-*d*_6_; δ, ppm) 10.19 (s, 1H), 8.25 (d, *J* = 8.0 Hz, 1H), 7.50 (d, *J* = 8.0 Hz, 1H), 7.42-7.33 (m, 3H), 7.29 (d, *J* = 8.8 Hz, 2H), 7.07 (m, 1H), 6.91-6.81 (m, 6H), 3.79 (m, 2H), 3.70 (s, 3H), 3.67 (s, 3H); ^13^C NMR (100 MHz, DMSO-*d*_6_; δ, ppm) 157.9, 154.7, 140.3, 138.1, 137.7, 137.3, 130.0, 128.5, 128.0, 127.3, 126.8, 125.2, 124.4, 123.8, 122.4, 121.5, 120.2, 119.2, 118.9, 113.8, 110.3, 105.0, 55.4, 53.7, 47.9, 32.7; IR (KBr, ν, cm^−1^) 3417, 2996, 1517, 1403, 1177, 1096, 1013, 914, 845, 720; HRMS (APCI-TOF) m/z calcd for C_28_H_22_NO_2_ [M-H]^−^ 404.1651; found 404.1638.

#### 1-(4-ethylphenyl)-1-(1-methyl-1H-indol-3-yl)-1,2-dihydrocyclobuta[a]naphthalen-4-ol (3i)

White solid, 97 mg, 80% yield; mp 165-167°C; ^1^H NMR (400 MHz, DMSO-*d*_6_; δ, ppm) 10.19 (s, 1H), 8.24 (d, *J* = 8.0 Hz, 1H), 7.50 (d, *J* = 8.0 Hz, 1H), 7.42-7.33 (m, 3H), 7.29 (d, *J* = 8.0 Hz, 2H), 7.13-7.04 (m, 3H), 6.90-6.77 (m, 4H), 3.80 (m, 2H), 3.68 (s, 3H), 2.56 (m, 2H), 1.15 (m, 3H); ^13^C NMR (100 MHz, DMSO-*d*_6_; δ, ppm) 154.7, 143.3, 141.7, 140.3, 137.7, 137.2, 130.0, 128.0, 127.9, 127.4, 127.4, 126.8, 125.2, 124.3, 123.8, 122.4, 121.5, 120.2, 119.0, 118.9, 110.3, 105.0, 54.0, 47.8, 32.7, 28.2, 16.0; IR (KBr, ν, cm^−1^) 3387, 3013, 1510, 1406, 1167, 1091, 1012, 918, 832, 722; HRMS (APCI-TOF) m/z calcd for C_29_H_24_NO [M-H]^−^ 402.1858; found 402.1874.

#### 1-(4-(tert-butyl)phenyl)-1-(1-methyl-1H-indol-3-yl)-1,2-dihydrocyclobuta[a]naphthalen-4-ol (3j)

White solid, 104 mg, 80% yield; mp 178-180°C; ^1^H NMR (400 MHz, DMSO-*d*_6_; δ, ppm) 10.19 (s, 1H), 8.24 (d, *J* = 8.4 Hz, 1H), 7.53 (d, *J* = 8.0 Hz, 1H), 7.35 (m, 7H), 7.07 (m, 1H), 6.93-6.78 (m, 4H), 3.81 (m, 2H), 3.68 (s, 3H), 1.25 (s, 9H); ^13^C NMR (100 MHz, DMSO-*d*_6_; δ, ppm) 154.7, 148.5, 143.0, 140.3, 137.7, 137.1, 130.1, 128.0, 127.4, 127.2, 126.8, 125.3, 125.2, 124.3, 123.8, 122.5, 121.5, 120.2, 119.0, 118.9, 110.3, 104.9, 54.0, 47.7, 34.5, 32.7, 31.6; IR (KBr, ν, cm^−1^) 3402, 3010, 1512, 1423, 1177, 1093, 1018, 933, 814, 711; HRMS (APCI-TOF) m/z calcd for C_31_H_28_NO [M-H]^−^ 430.2171; found 430.2187.

#### 1-(1-methyl-1H-indol-3-yl)-1-(naphthalen-1-yl)-1,2-dihydrocyclobuta[a]naphthalen-4-ol (3k)

White solid, 98 mg, 77% yield; mp 162-164°C; ^1^H NMR (400 MHz, DMSO-*d*_6_; δ, ppm) 10.25 (s, 1H), 8.27 (d, *J* = 8.0 Hz, 1H), 7.89-7.81 (m, 3H), 7.71 (d, *J* = 7.2 Hz, 1H), 7.57 (m, 2H), 7.47-7.34 (m, 5H), 7.08 (m, 1H), 6.95 (s, 1H), 6.91 (d, *J* = 6.0 Hz, 2H), 6.81 (m, 1H), 3.93 (s, 2H), 3.69 (m, 3H); ^13^C NMR (100 MHz, DMSO-*d*_6_; δ, ppm) 154.9, 143.7, 140.4, 137.8, 137.1, 133.3, 132.1, 130.1, 128.2, 128.1, 127.8, 127.5, 126.9, 126.6, 126.5, 126.0, 125.3, 125.2, 124.4, 123.9, 122.3, 121.5, 120.1, 119.0, 118.6, 110.4, 105.1, 54.5, 47.5, 32.7; IR (KBr, ν, cm^−1^) 3422, 3014, 1510, 1413, 1171, 1088, 1015, 937, 825, 727; HRMS (APCI-TOF) m/z calcd for C_31_H_22_NO [M-H]^−^ 424.1702; found 424.1713.

#### 1-(1-methyl-1H-indol-3-yl)-1-(thiophen-2-yl)-1,2-dihydrocyclobuta[a]naphthalen-4-ol (3l)

White solid, 104 mg, 91% yield; mp 158-160°C; ^1^H NMR (400 MHz, DMSO-*d*_6_; δ, ppm) 10.26 (s, 1H), 8.26 (d, *J* = 8.4 Hz, 1H), 7.71 (d, *J* = 8.4 Hz, 1H), 7.48 (m, 1H), 7.43-7.36 (m, 2H), 7.31 (d, *J* = 4.8 Hz, 1H), 7.12 (m, 2H), 6.99 (d, *J* = 5.6 Hz, 2H), 6.92 (m, 2H), 6.85 (s, 1H), 3.98 (d, *J* = 13.6 Hz, 1H), 3.77 (d, *J* = 13.6 Hz, 1H), 3.69 (s, 3H); ^13^C NMR (100 MHz, DMSO-*d*_6_; δ, ppm) 155.0, 151.2, 140.1, 137.6, 136.8, 129.5, 127.5, 126.9, 126.6, 125.3, 124.6, 124.5, 124.4, 124.0, 122.4, 121.6, 120.2, 119.1, 119.0, 110.4, 105.0, 50.9, 48.9, 32.7; IR (KBr, ν, cm^−1^) 3427, 3050, 1517, 1421, 1178, 1068, 1020, 936, 821, 734; HRMS (APCI-TOF) m/z calcd for C_25_H_18_NOS [M-H]^−^ 380.1110; found 380.1108.

#### 6-methoxy-1-(1-methyl-1H-indol-3-yl)-1-phenyl-1,2-dihydrocyclobuta[a]naphthalen-4-ol (3m)

White solid, 110 mg, 90% yield; mp 172-174°C; ^1^H NMR (400 MHz, DMSO-*d*_6_; δ, ppm) 10.11 (s, 1H), 7.58 (d, *J* = 2.4 Hz, 1H), 7.43 (d, *J* = 8.8 Hz, 1H), 7.37 (m, 3H), 7.29 (m, 2H), 7.20 (m, 1H), 7.11-7.05 (m, 2H), 6.88-6.79 (m, 4H), 3.87 (d, *J* = 13.2 Hz, 1H), 3.84 (s, 3H), 3.76 (d, *J* = 13.6 Hz, 1H), 3.68 (s, 3H); ^13^C NMR (100 MHz, DMSO-*d*_6_; δ, ppm) 156.1, 153.7, 146.1, 137.7, 137.3, 137.2, 128.5, 128.1, 127.5, 126.8, 126.4, 126.2, 125.5, 124.0, 121.5, 120.1, 119.6, 118.9, 110.3, 105.4, 103.2, 55.5, 54.3, 47.7, 32.7; IR (KBr, ν, cm^−1^) 3387, 3045, 1507, 1422, 1172, 1058, 1011, 932, 827, 739; HRMS (APCI-TOF) m/z calcd for C_28_H_22_NO_2_ [M-H]^−^ 404.1651; found 404.1630.

#### 1-(4-chlorophenyl)-6-methoxy-1-(1-methyl-1H-indol-3-yl)-1,2-dihydrocyclobuta[a]naphthalen-4-ol (3n)

White solid, 121 mg, 92% yield; mp 175-177°C; ^1^H NMR (400 MHz, DMSO-*d*_6_; δ, ppm) 10.15 (s, 1H), 7.59 (d, *J* = 2.4 Hz, 1H), 7.44-7.31 (m, 6H), 7.13-7.05 (m, 2H), 6.94-6.81 (m, 4H), 3.85 (s, 3H), 3.83-3.72 (m, 2H), 3.68 (s, 3H); ^13^C NMR (100 MHz, DMSO-*d*_6_; δ, ppm) 156.2, 153.9, 145.1, 137.7, 137.2, 136.7, 131.0, 129.3, 128.5, 128.1, 126.7, 126.3, 125.3, 123.8, 121.6, 120.0, 119.7, 119.1, 118.3, 110.4, 105.4, 103.2, 55.5, 53.7, 47.7, 32.7. IR (KBr, ν, cm^−1^) 3382, 3025, 1523, 1421, 1192, 1074, 1008, 934, 828, 736. HRMS (APCI-TOF) m/z calcd for C_28_H_21_NClO_2_ [M-H]^−^ 438.1261; found 438.1277.

#### 6-methoxy-1-(1-methyl-1H-indol-3-yl)-1-(p-tolyl)-1,2-dihydrocyclobuta[a]naphthalen-4-ol (3o)

White solid, 102 mg, 81% yield; mp 170-172°C; ^1^H NMR (400 MHz, DMSO-*d*_6_; δ, ppm) 10.09 (s, 1H), 7.59 (d, *J* = 2.4 Hz, 1H), 7.43 (d, *J* = 9.2 Hz, 1H), 7.35 (d, *J* = 8.0 Hz, 1H), 7.26 (d, *J* = 8.0 Hz, 2H), 7.07 (m, 4H), 6.89-6.80 (m, 4H), 3.84 (s, 3H), 3.83-3.72 (m, 2H), 3.67 (s, 3H), 2.26 (s, 3H); ^13^C NMR (100 MHz, DMSO-*d*_6_; δ, ppm) 156.1, 153.6, 143.1, 137.7, 137.4, 137.3, 135.3, 129.1, 128.0, 127.4, 126.8, 126.2, 125.5, 124.0, 121.5, 120.2, 119.5, 119.1, 118.9, 110.2, 105.4, 103.1, 55.5, 54.0, 47.7, 32.6, 21.1; IR (KBr, ν, cm^−1^) 3387, 3022, 1521, 1425, 1202, 1094, 1016, 942, 835, 721; HRMS (APCI-TOF) m/z calcd for C_29_H_24_NO_2_ [M-H]^−^ 418.1808; found 418.1822.

#### 7-methyl-1-(1-methyl-1H-indol-3-yl)-1-phenyl-1,2-dihydrocyclobuta[a]naphthalen-4-ol (3p)

White solid, 84 mg, 72% yield; mp 147-149°C; ^1^H NMR (400 MHz, DMSO-*d*_6_; δ, ppm) 10.12 (s, 1H), 8.14 (d, *J* = 8.8 Hz, 1H), 7.38 (m, 3H), 7.32-7.26 (m, 3H), 7.20 (m, 2H), 7.08 (m, 1H), 6.88 (d, *J* = 4.4 Hz, 2H), 6.86-6.77 (m, 2H), 3.86 (d, *J* = 13.6 Hz, 1H), 3.73 (d, *J* = 13.6 Hz, 1H), 3.69 (s, 3H), 2.32 (s, 3H); ^13^C NMR (100 MHz, DMSO-*d*_6_; δ, ppm) 154.8, 146.2, 140.5, 137.7, 136.6, 136.3, 130.4, 128.5, 128.1, 127.5, 126.8, 126.4, 126.0, 124.3, 123.5, 121.5, 121.3, 120.2, 118.9, 118.9, 110.3, 104.2, 54.4, 47.9, 32.7, 22.0; IR (KBr, ν, cm^−1^) 3507, 3052, 1522, 1420, 1201, 1098, 1015, 944, 841, 727; HRMS (APCI-TOF) m/z calcd for C_28_H_22_NO [M-H]^−^ 388.1702; found 388.1727.

#### 1-(4-chlorophenyl)-7-methyl-1-(1-methyl-1H-indol-3-yl)-1,2-dihydrocyclobuta[a]naphthalen-4-ol (3q)

White solid, 114 mg, 90% yield; mp 152-154°C; ^1^H NMR (400 MHz, DMSO-*d*_6_; δ, ppm) 10.16 (s, 1H), 8.14 (d, *J* = 8.4 Hz, 1H), 7.40-7.33 (m, 5H), 7.25 (s, 1H), 7.19 (d, *J* = 8.8 Hz, 1H), 7.09 (m, 1H), 6.94-6.83 (m, 3H), 6.79 (s, 1H), 3.85 (d, *J* = 13.6 Hz, 1H), 3.73 (s, 1H), 3.70 (s, 3H), 2.33 (s, 3H); ^13^C NMR (100 MHz, DMSO-*d*_6_; δ, ppm) 154.9, 145.2, 140.4, 137.7, 136.7, 135.9, 131.0, 130.2, 129.4, 128.5, 128.2, 126.7, 126.1, 124.4, 123.5, 121.6, 121.1, 120.1, 119.1, 118.3, 110.4, 104.2, 53.7, 47.9, 32.7, 22.0; IR (KBr, ν, cm^−1^) 3495, 3042, 1520, 1402, 1221, 1084, 1016, 947, 831, 709; HRMS (APCI-TOF) m/z calcd for C_28_H_21_ClNO [M-H]^−^ 422.1312; found 422.1314.

#### 7-methyl-1-(1-methyl-1H-indol-3-yl)-1-(p-tolyl)-1,2-dihydrocyclobuta[a]naphthalen-4-ol (3r)

White solid, 96 mg, 79% yield; mp 153-155°C; ^1^H NMR (400 MHz, DMSO-*d*_6_; δ, ppm) 10.10 (s, 1H), 8.14 (d, *J* = 8.4 Hz, 1H), 7.36 (d, *J* = 8.4 Hz, 1H), 7.26 (d, *J* = 8.0 Hz, 3H), 7.18 (d, *J* = 8.8 Hz, 1H), 7.08 (m, 3H), 6.90 (d, *J* = 8.8 Hz, 2H), 6.83 (m, 1H), 6.79 (s, 1H), 3.88-3.69 (m, 2H), 3.68 (s, 3H), 2.32 (s, 3H), 2.26 (s, 3H); ^13^C NMR (100 MHz, DMSO-*d*_6_; δ, ppm) 154.7, 143.1, 140.4, 137.7, 136.5, 136.5, 135.3, 130.3, 129.1, 128.0, 127.4, 126.9, 126.0, 124.3, 123.4, 121.5, 121.3, 120.3, 119.0, 118.9, 110.3, 104.2, 54.0, 47.9, 32.7, 22.0, 21.1; IR (KBr, ν, cm^−1^) 3490, 3031, 1500, 1422, 1213, 1090, 1012, 948, 843, 719; HRMS (APCI-TOF) m/z calcd for C_29_H_24_NO [M-H]^−^ 402.1858; found 402.1844.

#### 7-fluoro-1-(1-methyl-1H-indol-3-yl)-1-phenyl-1,2-dihydrocyclobuta[a]naphthalen-4-ol (3s)

White solid, 96 mg, 81% yield; mp 144-146°C; ^1^H NMR (400 MHz, DMSO-*d*_6_; δ, ppm) 10.36 (s, 1H), 7.87 (m, 1H), 7.55 (m, 1H), 7.33 (m, 6H), 7.21 (m, 1H), 7.08 (m, 1H), 6.93 (s, 1H), 6.91-6.77 (m, 3H), 3.84 (m, 2H), 3.68 (s, 3H). ^13^C NMR (100 MHz, DMSO-*d*_6_; δ, ppm) 160.3 (^1^*J*_CF_ = 239.4 Hz), 157.9, 154.2, 154.1, 145.8, 139.7 (^6^*J*_CF_ = 2.4 Hz), 139.6, 137.7, 137.3, 128.6, 128.1, 127.5, 127.2, 126.8, 126.7 (^3^*J*_CF_ = 19.7 Hz), 125.9 (^5^*J*_CF_ = 8.1 Hz), 125.8, 125.1 (^4^*J*_CF_ = 8.4 Hz), 125.0, 121.5, 120.0, 119.0, 118.6, 117.5, 117.3, 110.3, 108.0 (^2^*J*_CF_ = 21.7 Hz), 107.8, 106.0, 54.4, 47.8, 32.7; IR (KBr, ν, cm^−1^) 3501, 3021, 1502, 1421, 1215, 1099, 1014, 962, 883, 712; HRMS (APCI-TOF) m/z calcd for C_27_H_19_FNO [M-H]^−^ 392.1451; found 392.1459.

#### 1-(4-chlorophenyl)-7-fluoro-1-(1-methyl-1H-indol-3-yl)-1,2-dihydrocyclobuta[a]naphthalen-4-ol (3t)

White solid, 117 mg, 91% yield; mp 149-151°C; ^1^H NMR (400 MHz, DMSO-*d*_6_; δ, ppm) 10.44 (s, 1H), 8.30 (m, 1H), 7.41-7.32 (m, 5H), 7.24 (m, 1H), 7.12-7.04 (m, 2H), 6.98 (s, 1H), 6.86 (d, *J* = 13.2 Hz, 3H), 3.82 (m, 2H), 3.71 (s, 3H); ^13^C NMR (100 MHz, DMSO-*d*_6_; δ, ppm) 160.0 (^1^*J*_CF_ = 231.6 Hz), 157.1, 155.3, 144.8, 142.4, 137.8, 136.2 (^6^*J*_CF_ = 3.4 Hz), 136.1, 131.1, 130.5 (^5^*J*_CF_ = 9.6 Hz), 130.4, 129.3, 128.6, 128.2, 127.8 (^4^*J*_CF_ = 9.9 Hz), 126.6, 122.4, 121.6, 119.9, 119.2, 117.8, 113.9 (^2^*J*_CF_ = 24.1 Hz), 113.6, 110.5, 105.5 (^3^*J*_CF_ = 20.3 Hz), 105.3, 104.5, 53.7, 47.7, 32.7; IR (KBr, ν, cm^−1^) 3512, 3020, 1505, 1411, 1210, 1095, 1010, 966, 861, 740; HRMS (APCI-TOF) m/z calcd for C_27_H_18_ClFNO [M-H]^−^ 426.1061; found 426.1066.

#### 6-fluoro-1-(1-methyl-1H-indol-3-yl)-1-phenyl-1,2-dihydrocyclobuta[a]naphthalen-4-ol (3u)

White solid, 97 mg, 82% yield; mp 148-150°C; ^1^H NMR (400 MHz, DMSO-*d*_6_; δ, ppm) 10.41 (s, 1H), 8.30 (m, 1H), 7.38 (d, *J* = 8.8 Hz, 3H), 7.30 (m, 2H), 7.24 (m, 2H), 7.08 (m, 2H), 6.94 (s, 1H), 6.90-6.79 (m, 3H), 3.84 (m, 2H), 3.70 (s, 3H); ^13^C NMR (100 MHz, DMSO-*d*_6_; δ, ppm) 162.3 (^1^*J*_CF_ = 243.8 Hz), 160.0, 155.1, 145.8, 142.4, 137.8, 136.7 (^6^*J*_CF_ = 5.1 Hz), 130.7 (^5^*J*_CF_ = 9.3 Hz), 130.6, 128.6, 128.1, 127.8, 127.7 (^4^*J*_CF_ = 9.6 Hz), 127.4, 126.7, 126.6, 122.4, 121.5, 120.0, 119.0, 118.4, 113.8 (^2^*J*_CF_ = 24.8 Hz), 113.5, 110.4, 105.7 (^3^*J*_CF_ = 20.1 Hz), 105.5, 104.6, 54.3, 47.7, 32.7; IR (KBr, ν, cm^−1^) 3502, 3047, 1502, 1422, 1235, 1099, 1015, 946, 851, 733; HRMS (APCI-TOF) m/z calcd for C_27_H_19_FNO [M-H]^−^ 392.1451; found 392.1450.

#### 8-(1-methyl-1H-indol-3-yl)-8-(p-tolyl)-7,8-dihydrocyclobuta[h]quinolin-5-ol (3v)

White solid, 80 mg, 68% yield; mp 162-164°C; ^1^H NMR (400 MHz, DMSO-*d*_6_; δ, ppm) 10.56 (s, 1H), 8.88 (m, 1H), 8.60 (d, *J* = 8.4 Hz, 1H), 7.72 (d, *J* = 8.0 Hz, 2H), 7.41 (dd, *J* = 8.4, 4.2 Hz, 1H), 7.32 (d, *J* = 8.0 Hz, 1H), 7.23 (d, *J* = 8.0 Hz, 1H), 7.07 (d, *J* = 8.0 Hz, 3H), 7.01 (s, 1H), 6.90 (s, 1H), 6.85 (m, 1H), 3.91 (m, 2H), 3.64 (s, 3H), 2.23 (s, 3H); ^13^C NMR (100 MHz, DMSO-*d*_6_; δ, ppm) 155.0, 151.2, 145.1, 143.9, 137.5, 137.1, 135.0, 132.7, 128.8, 128.3, 128.1, 126.5, 121.3, 120.4, 120.3, 120.1, 119.3, 118.8, 110.1, 105.3, 55.2, 46.1, 32.6, 21.1; IR (KBr, ν, cm^−1^) 3545, 3067, 1552, 1421, 1230, 1129, 1012, 949, 840, 730; HRMS (APCI-TOF) m/z calcd for C_27_H_21_N_2_O [M-H]^−^ 389.1654; found 389.1657.

#### 1-(4-chlorophenyl)-1-(5-methoxy-1-methyl-1H-indol-3-yl)-1,2-dihydrocyclobuta[a]naphthalen-4-ol (3w)

White solid, 121 mg, 92% yield; mp 166-168°C; ^1^H NMR (400 MHz, DMSO-*d*_6_; δ, ppm) 10.27 (s, 1H), 8.27 (d, *J* = 8.0 Hz, 1H), 7.48 (d, *J* = 8.0 Hz, 1H), 7.44-7.33 (m, 6H), 7.27 (d, *J* = 8.8 Hz, 1H), 6.88 (d, *J* = 10.8 Hz, 2H), 6.74 (m, 1H), 6.24 (d, *J* = 2.0 Hz, 1H), 3.81 (m, 2H), 3.64 (s, 3H), 3.49 (s, 3H); ^13^C NMR (100 MHz, DMSO-*d*_6_; δ, ppm) 155.0, 153.3, 145.0, 140.5, 136.5, 133.1, 131.1, 130.0, 129.4, 128.7, 128.5, 127.6, 127.0, 125.3, 124.4, 124.0, 122.2, 117.6, 111.0, 110.9, 104.8, 102.4, 55.5, 53.8, 47.7, 32.8; IR (KBr, ν, cm^−1^) 3504, 2997, 1534, 1401, 1233, 1149, 1010, 942, 847, 736; HRMS (APCI-TOF) m/z calcd for C_28_H_21_NClO_2_ [M-H]^−^ 438.1261; found 438.1267.

#### 1-(5-methoxy-1-methyl-1H-indol-3-yl)-1-(p-tolyl)-1,2-dihydrocyclobuta[a]naphthalen-4-ol (3x)

White solid, 107 mg, 85% yield; mp 170-172°C; ^1^H NMR (400 MHz, DMSO-*d*_6_; δ, ppm) 10.21 (s, 1H), 8.25 (d, *J* = 8.0 Hz, 1H), 7.49 (d, *J* = 8.0 Hz, 1H), 7.43-7.33 (m, 2H), 7.29 (d, *J* = 8.0 Hz, 2H), 7.25 (d, *J* = 8.8 Hz, 1H), 7.11 (d, *J* = 7.6 Hz, 2H), 6.88 (s, 1H), 6.82 (s, 1H), 6.72 (m, 1H), 6.22 (d, *J* = 2.0 Hz, 1H), 3.79 (m, 2H), 3.63 (s, 3H), 3.47 (s, 3H), 2.27 (s, 3H); ^13^C NMR (100 MHz, DMSO-*d*_6_; δ, ppm) 154.8, 153.1, 143.0, 140.5, 137.1, 135.3, 133.1, 130.2, 129.1, 128.6, 127.5, 127.4, 127.1, 125.2, 124.4, 123.8, 122.4, 118.4, 110.8, 110.7, 104.9, 102.6, 55.5, 54.1, 47.7, 32.8, 21.1; IR (KBr, ν, cm^−1^) 3487, 3022, 1520, 1422, 1200, 1097, 1012, 944, 833, 725; HRMS (APCI-TOF) m/z calcd for C_29_H_24_NO_2_ [M-H]^−^ 418.1808; found 418.1838.

#### 1-(7-chloro-1-methyl-1H-indol-3-yl)-1-(p-tolyl)-1,2-dihydrocyclobuta[a]naphthalen-4-ol (3y)

White solid, 113 mg, 89% yield; mp 163-165°C; ^1^H NMR (400 MHz, DMSO-*d*_6_; δ, ppm) 10.24 (s, 1H), 8.24 (d, *J* = 8.0 Hz, 1H), 7.48 (d, *J* = 7.6 Hz, 1H), 7.38 (m, 2H), 7.23 (d, *J* = 8.0 Hz, 2H), 7.06 (d, *J* = 8.4 Hz, 2H), 6.93 (s, 1H), 6.88-6.76 (m, 4H), 3.99 (s, 3H), 3.78 (s, 2H), 2.24 (s, 3H); ^13^C NMR (100 MHz, DMSO-*d*_6_; δ, ppm) 154.8, 142.5, 140.2, 136.9, 135.5, 132.6, 131.2, 130.2, 129.9, 129.2, 127.4, 127.3, 125.2, 124.4, 123.9, 123.0, 122.2, 120.1, 119.6, 119.3, 116.5, 104.9, 53.6, 47.6, 36.5, 21.0; IR (KBr, ν, cm^−1^) 3505, 3022, 1524, 1400, 1209, 1082, 1012, 945, 833, 719; HRMS (APCI-TOF) m/z calcd for C_28_H_21_ClNO [M-H]^−^ 422.1312; found 422.1304.

#### 1-(1,7-dimethyl-1H-indol-3-yl)-1-(p-tolyl)-1,2-dihydrocyclobuta[a]naphthalen-4-ol (3z)

White solid, 93 mg, 77% yield; mp 165-167°C; ^1^H NMR (400 MHz, DMSO-*d*_6_; δ, ppm) 10.17 (s, 1H), 8.23 (d, *J* = 8.0 Hz, 1H), 7.48 (d, *J* = 8.0 Hz, 1H), 7.37 (m, 2H), 7.22 (d, *J* = 8.0 Hz, 2H), 7.06 (d, *J* = 8.0 Hz, 2H), 6.85 (s, 1H), 6.79-6.64 (m, 4H), 3.93 (s, 3H), 3.77 (s, 2H), 2.68 (s, 3H), 2.25 (s, 3H). ^13^C NMR (100 MHz, DMSO-*d*_6_; δ, ppm) 154.6, 143.0, 140.3, 137.4, 136.3, 135.3, 129.9, 129.6, 129.1, 128.0, 127.3, 125.2, 124.3, 124.0, 123.8, 122.4, 121.8, 119.1, 118.5, 118.4(5), 118.3(9), 105.0, 53.8, 47.6, 36.6, 21.1, 19.7; IR (KBr, ν, cm^−1^) 3468, 3056, 1480, 1424, 1203, 1095, 1010, 958, 844, 722; HRMS (APCI-TOF) m/z calcd for C_29_H_24_NO [M-H]^−^ 402.1858; found 402.1844.

#### 1-(6-methoxy-1-methyl-1H-indol-3-yl)-1-(p-tolyl)-1,2-dihydrocyclobuta[a]naphthalen-4-ol (3aa)

White solid, 96 mg, 76% yield; mp 171-173°C; ^1^H NMR (400 MHz, DMSO-*d*_6_; δ, ppm) 10.18 (s, 1H), 8.23 (d, *J* = 8.0 Hz, 1H), 7.48 (d, *J* = 8.0 Hz, 1H), 7.41-7.32 (m, 2H), 7.26 (d, *J* = 8.0 Hz, 2H), 7.08 (d, *J* = 8.0 Hz, 2H), 6.89 (d, *J* = 2.0 Hz, 1H), 6.85 (s, 1H), 6.70 (d, *J* = 10.4 Hz, 2H), 6.48 (m, 1H), 3.81 (m, 2H), 3.75 (s, 3H), 3.63 (s, 3H), 2.26 (s, 3H); ^13^C NMR (100 MHz, DMSO-*d*_6_; δ, ppm) 156.0, 154.6, 143.1, 140.3, 138.5, 137.3, 135.3, 130.0, 129.1, 127.4, 127.3, 126.8, 125.2, 124.3, 123.8, 122.4, 121.1, 120.7, 119.1, 109.0, 105.0, 93.6, 55.7, 54.0, 47.8, 32.7, 21.1; IR (KBr, ν, cm^−1^) 3487, 3020, 1521, 1420, 1212, 1091, 1011, 944, 836, 729; HRMS (APCI-TOF) m/z calcd for C_29_H_24_NO_2_ [M-H]^−^ 418.1808; found 418.1822.

#### 1-(5-bromo-1-methyl-1H-indol-3-yl)-1-(p-tolyl)-1,2-dihydrocyclobuta[a]naphthalen-4-ol (3bb)

White solid, 121 mg, 76% yield; mp 175-177°C; ^1^H NMR (400 MHz, DMSO-*d*_6_; δ, ppm) 10.22 (s, 1H), 8.25 (d, *J* = 8.0 Hz, 1H), 7.46 (d, *J* = 8.0 Hz, 1H), 7.40 (m, 3H), 7.25 (d, *J* = 8.4 Hz, 2H), 7.20 (d, *J* = 8.0 Hz, 1H), 7.10 (d, *J* = 8.0 Hz, 2H), 6.95 (s, 1H), 6.93 (s, 1H), 6.86 (s, 1H), 3.84-3.74 (m, 2H), 3.69 (s, 3H), 2.27 (s, 3H); ^13^C NMR (100 MHz, DMSO-*d*_6_; δ, ppm) 154.8, 142.5, 140.3, 136.8, 136.5, 135.5, 129.9, 129.6, 129.2, 128.4, 127.5, 127.3, 125.2, 124.4, 124.0, 122.2, 118.8, 112.6, 111.6, 104.9, 53.7, 47.8, 32.9, 21.1; IR (KBr, ν, cm^−1^) 3531, 3050, 1522, 1404, 1242, 1121, 1017, 945, 836, 720; HRMS (APCI-TOF) m/z calcd for C_28_H_21_BrNO [M-H]^−^ 466.0807; found 466.0815.

#### 1-(1H-indol-3-yl)-1-(p-tolyl)-1,2-dihydrocyclobuta[a]naphthalen-4-ol (3cc)

White solid, 88 mg, 78% yield; mp 156-158°C; ^1^H NMR (400 MHz, DMSO-*d*_6_; δ, ppm) 10.86 (s, 1H), 10.20 (s, 1H), 8.26 (d, *J* = 8.4 Hz, 1H), 7.52 (d, *J* = 8.0 Hz, 1H), 7.38 (m, 3H), 7.27 (d, *J* = 8.4 Hz, 2H), 7.08 (d, *J* = 8.0 Hz, 2H), 7.02 (m, 1H), 6.96-6.88 (m, 3H), 6.81 (m, 1H), 3.82 (m, 2H), 2.25 (s, 3H); ^13^C NMR (100 MHz, DMSO-*d*_6_; δ, ppm) 154.6, 143.2, 140.3, 137.4, 137.4, 135.3, 130.0, 129.1, 127.4, 127.3, 126.6, 125.2, 124.4, 123.8, 123.7, 122.4, 122.0, 121.3, 120.0, 119.7, 118.8, 112.1, 105.0, 54.1, 47.6, 21.1; IR (KBr, ν, cm^−1^) 3501, 3408, 3051, 1520, 1402, 1240, 1118, 1010, 943, 816, 723; HRMS (APCI-TOF) m/z calcd for C_27_H_20_NO [M-H]^−^ 374.1545; found 374.1529.

#### 1-(6-methyl-1H-indol-3-yl)-1-phenyl-1,2-dihydrocyclobuta[a]naphthalen-4-ol (3dd)

White solid, 91 mg, 81% yield; mp 172-174°C; ^1^H NMR (400 MHz, DMSO-*d*_6_; δ, ppm) 10.72 (s, 1H), 10.18 (s, 1H), 8.25 (d, *J* = 8.4 Hz, 1H), 7.52 (d, *J* = 8.0 Hz, 1H), 7.44-7.34 (m, 4H), 7.26 (m, 3H), 7.18 (m, 1H), 6.86 (d, *J* = 6.8 Hz, 3H), 6.79 (s, 1H), 3.83 (s, 2H), 2.22 (s, 3H); ^13^C NMR (100 MHz, DMSO-*d*_6_; δ, ppm) 154.6, 146.2, 140.4, 137.5, 135.7, 129.9, 128.5, 127.3, 127.1, 126.9, 126.3, 125.2, 124.4, 123.8, 123.0, 122.4, 119.6, 119.0, 111.9, 105.0, 54.2, 47.5, 21.9; IR (KBr, ν, cm^−1^) 3518, 3401, 3050, 1570, 1392, 1242, 1110, 1023, 941, 826, 721; HRMS (APCI-TOF) m/z calcd for C_27_H_20_NO [M-H]^−^ 374.1545; found 374.1553.

#### X-Ray Structure of Product 3a (CCDC 1867087)

The crystal of compound **3a** belongs to Triclinic, space group *P-1* with *a* = 8.5599(7) Å, *b* = 12.1512(11) Å, *c* = 12.5112(12) Å, α = 100.943(2)°, β = 94.2510(10)°, γ = 106.823(3)°, *V* = 1211.40(19) Å^3^, *Mr* = 433.53, *Z* = 2, *Dc* = 1.743 g/cm^3^, μ(Mo*K*α) = 0.074 mm^−1^, *F*(000) = 460, the final *R* = 0.0495 and *wR* = 0.1118.

## Author Contributions

HL, BJ, and GL designed the project. HL performed the experiments. HL, W-JH, and S-JT analyzed the data. HL, BJ, and GL wrote the manuscript.

### Conflict of Interest Statement

The authors declare that the research was conducted in the absence of any commercial or financial relationships that could be construed as a potential conflict of interest.
