# Supplementary material for: Fe(III)-Catalyzed Bicyclization of Yne-Allenones With Indoles for the Atom-Economic Synthesis of 3-Indolyl Cyclobutarenes
Source: Front Chem. 2018 Dec 4;6:599. doi: 10.3389/fchem.2018.00599 (PMC6288644; doi:10.3389/fchem.2018.00599)
Supplement: Supplementary file 1 [file Data_Sheet_1.PDF]

Supporting Information

Fe(III)-Catalyzed Bicylization of Yne-Allenones with Indoles for Atom-Economic Synthesis of 3-Indolyl Cyclobutarenes

Heng Li[1], Wen-Juan Hao[1], Guigen Li[2,3*], Shu-Jiang Tu[1], Bo Jiang[1*]

[1]School of Chemistry & Materials Science, Jiangsu Key Laboratory of Green Synthetic Chemistry for Functional Materials, Jiangsu Normal University, Xuzhou 221116, P. R. China

[2]Institute of Chemistry & BioMedical Sciences, Collaborative Innovation Center of Chemistry for Life Sciences, Nanjing University, Nanjing 210093, P. R. China

[3]Department of Chemistry and Biochemistry, Texas Tech University, Lubbock, Texas 79409-1061, United States.

Email: guigen.li@ttu.edu; jiangchem@jsnu.edu.cn

Context

Copies of ^{1}H and ^{13}C NMR Spectra for Compounds **3a-3dd**................................S2-S61

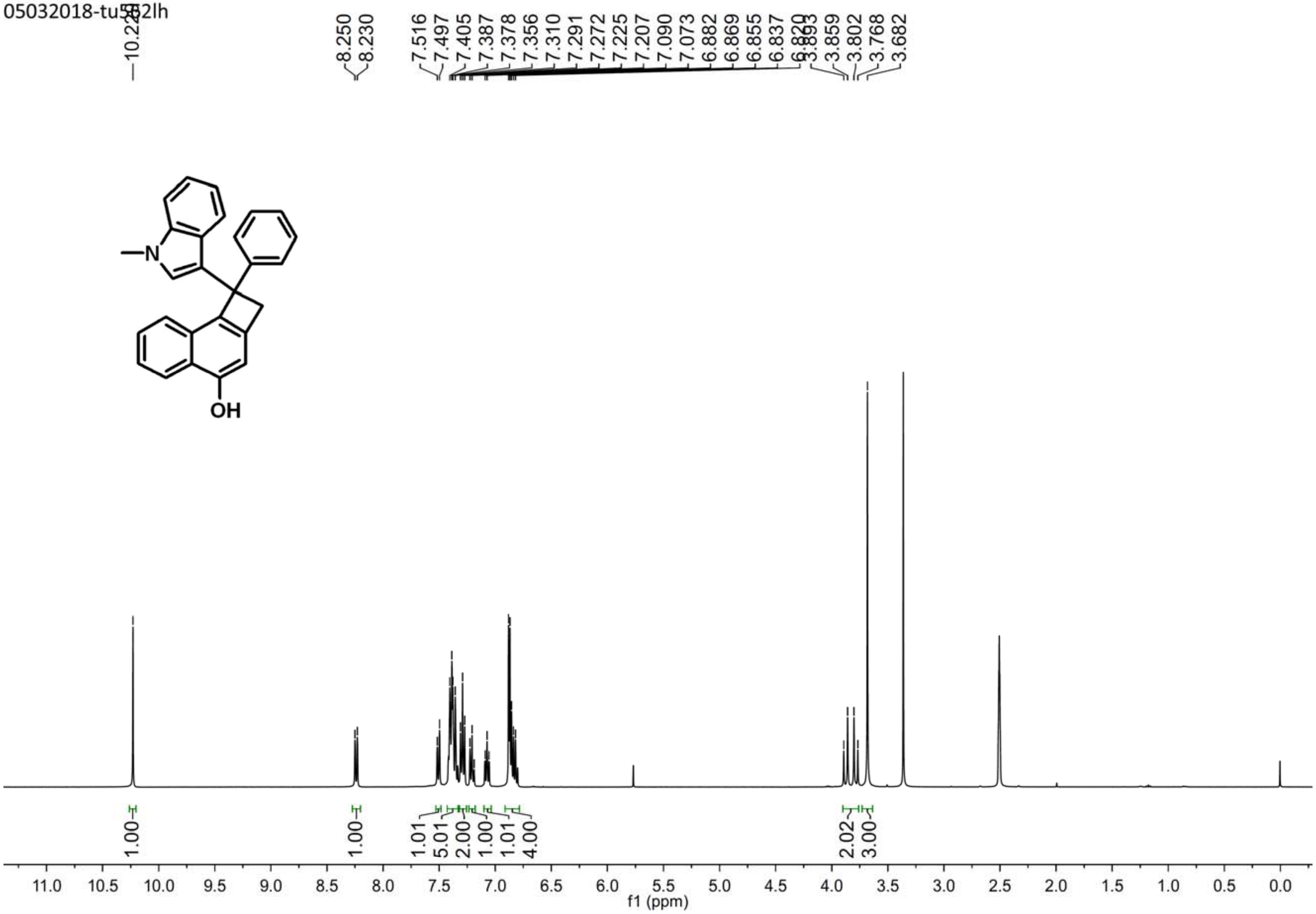

^{1}H NMR Spectrum of Compound 3a

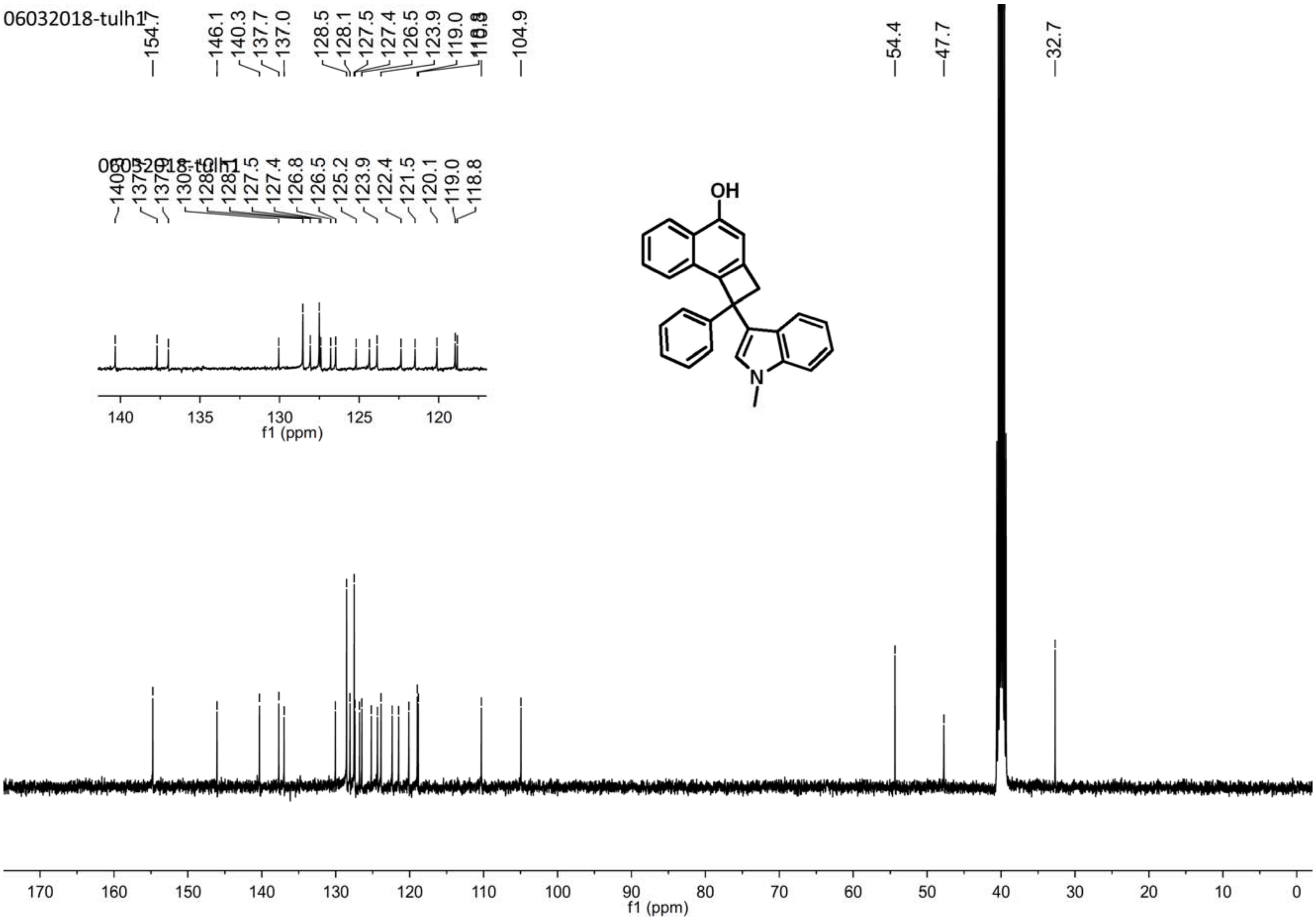

^{13}C NMR Spectrum of Compound 3a

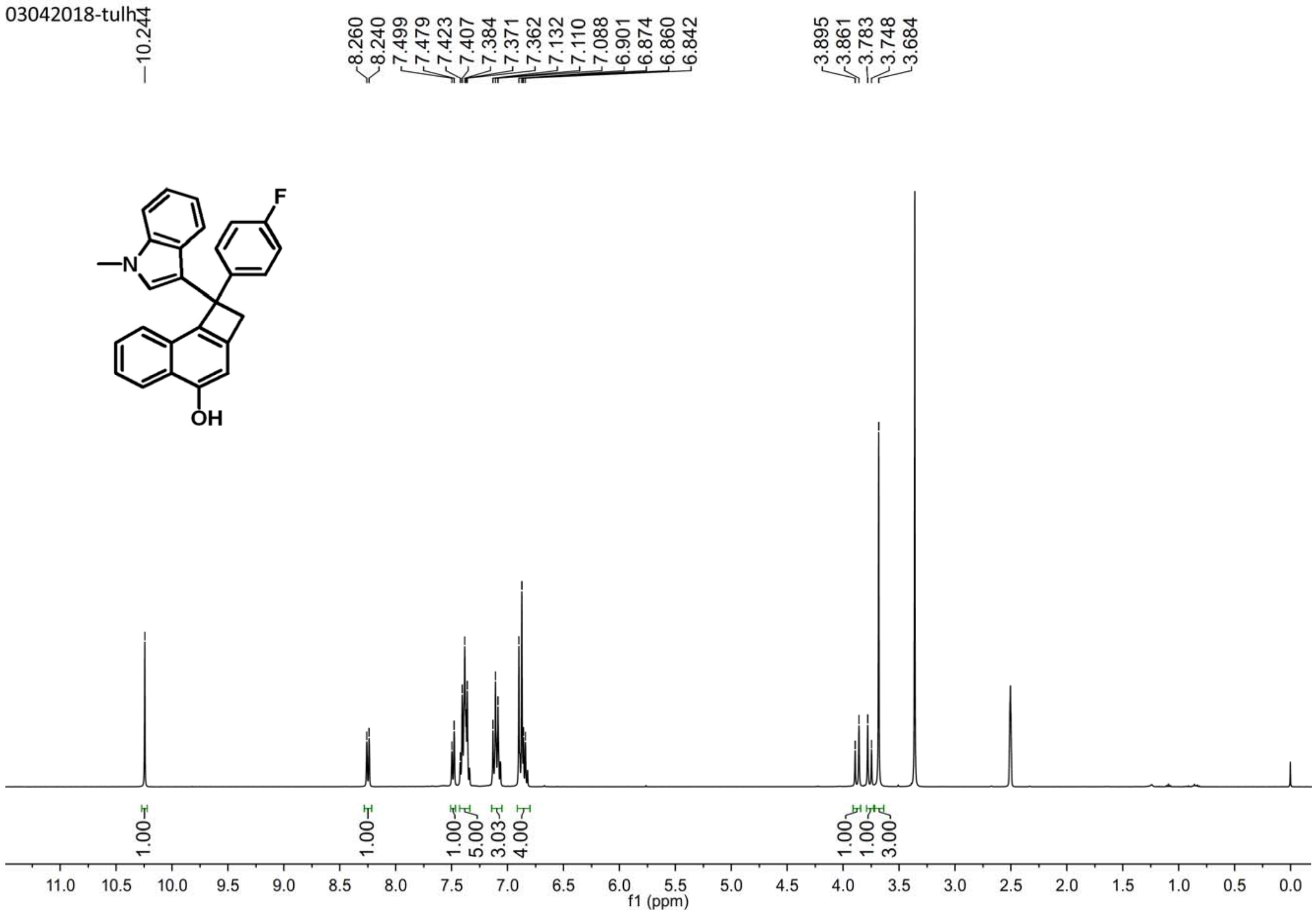

^{1}H NMR Spectrum of Compound 3b

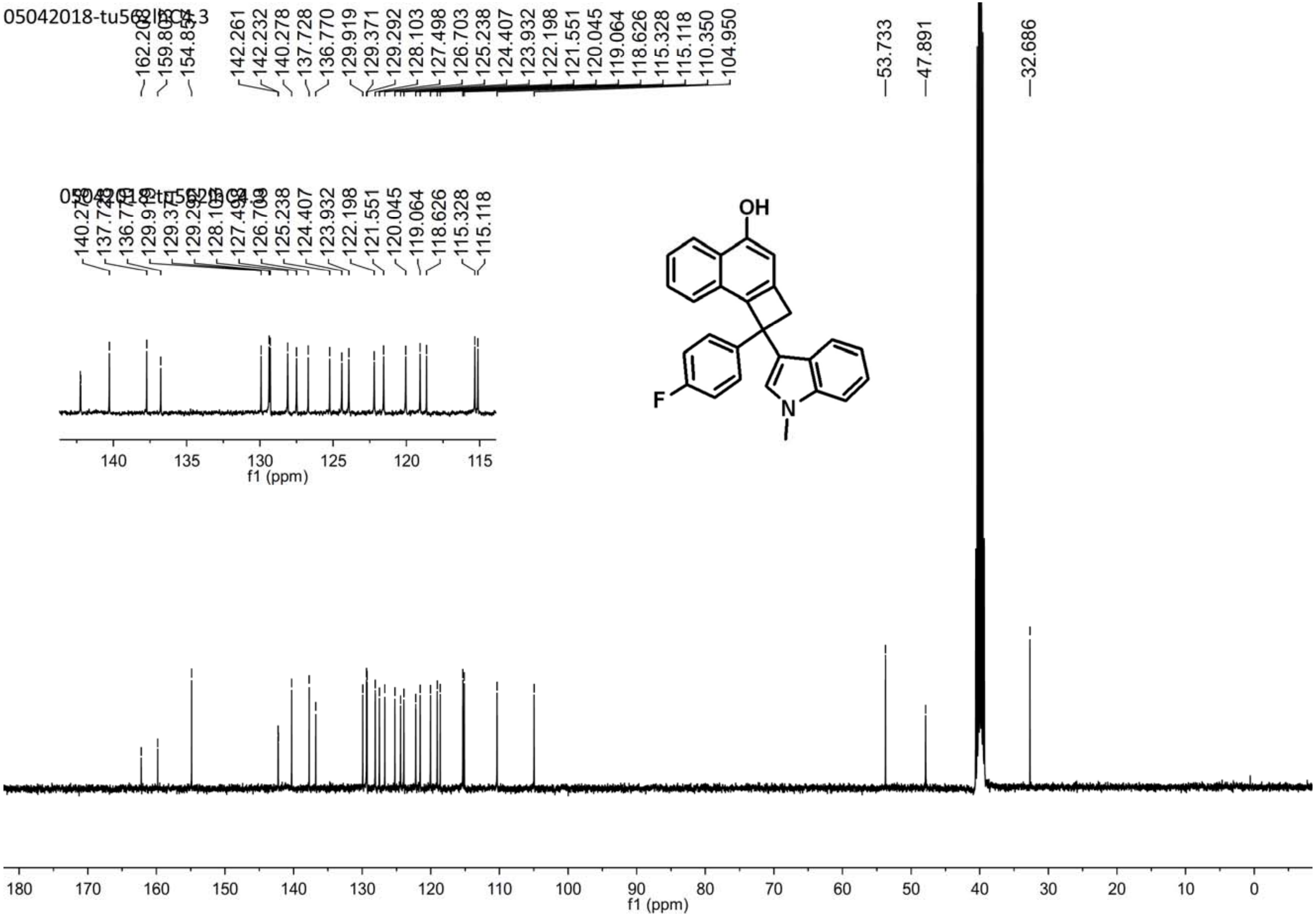

^{13}C NMR Spectrum of Compound 3b

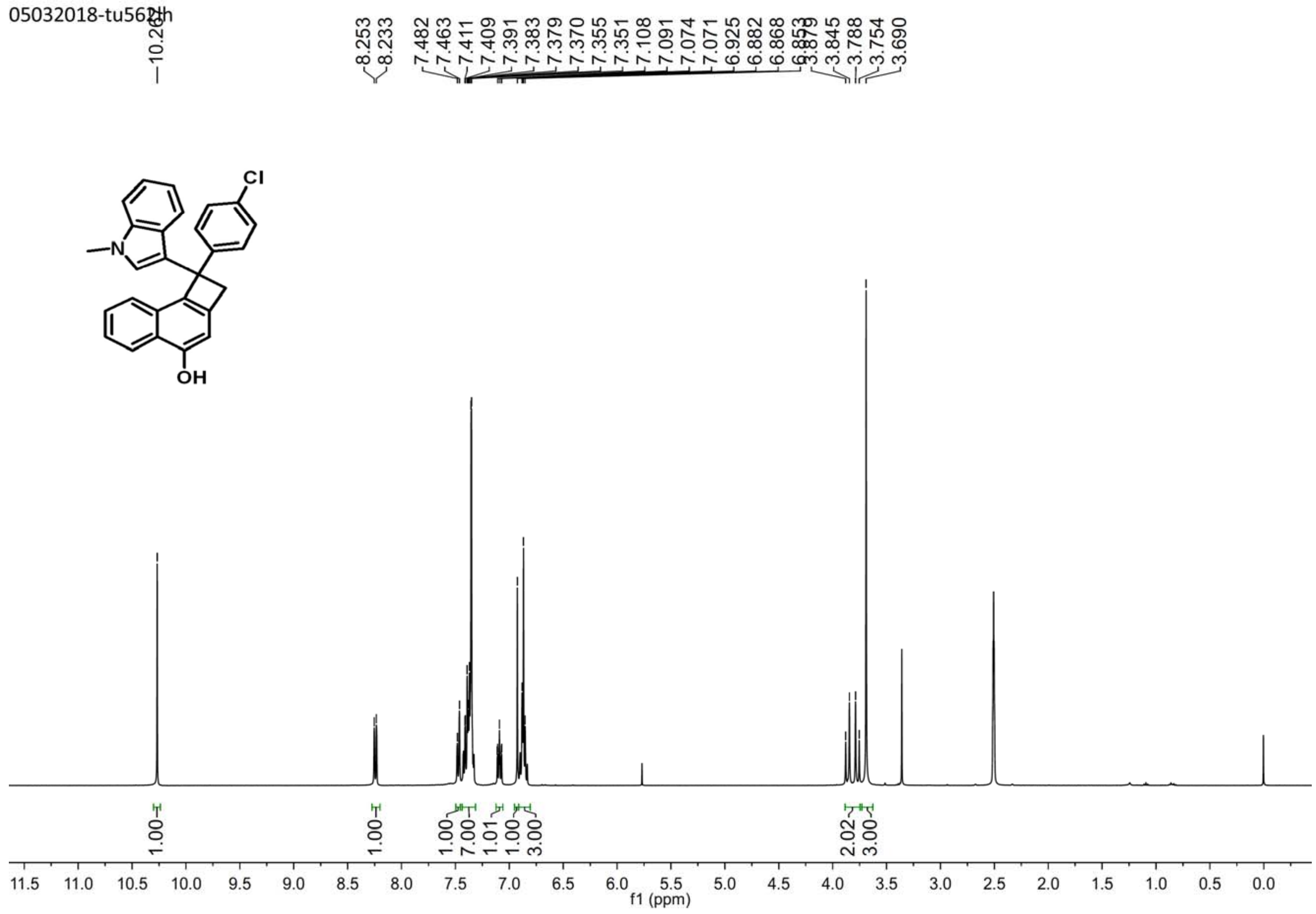

^{1}H NMR Spectrum of Compound 3c

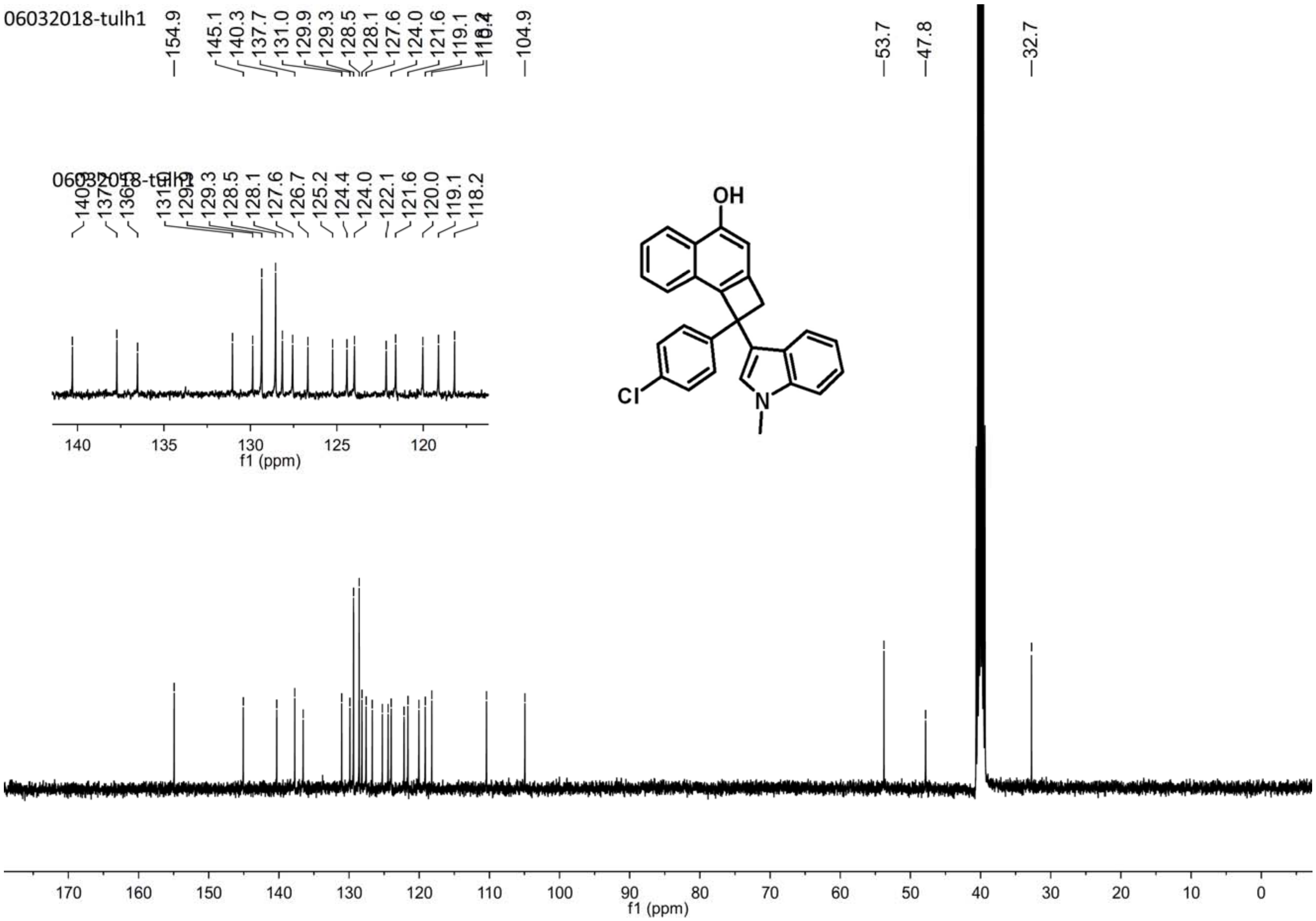

^{13}C NMR Spectrum of Compound 3c

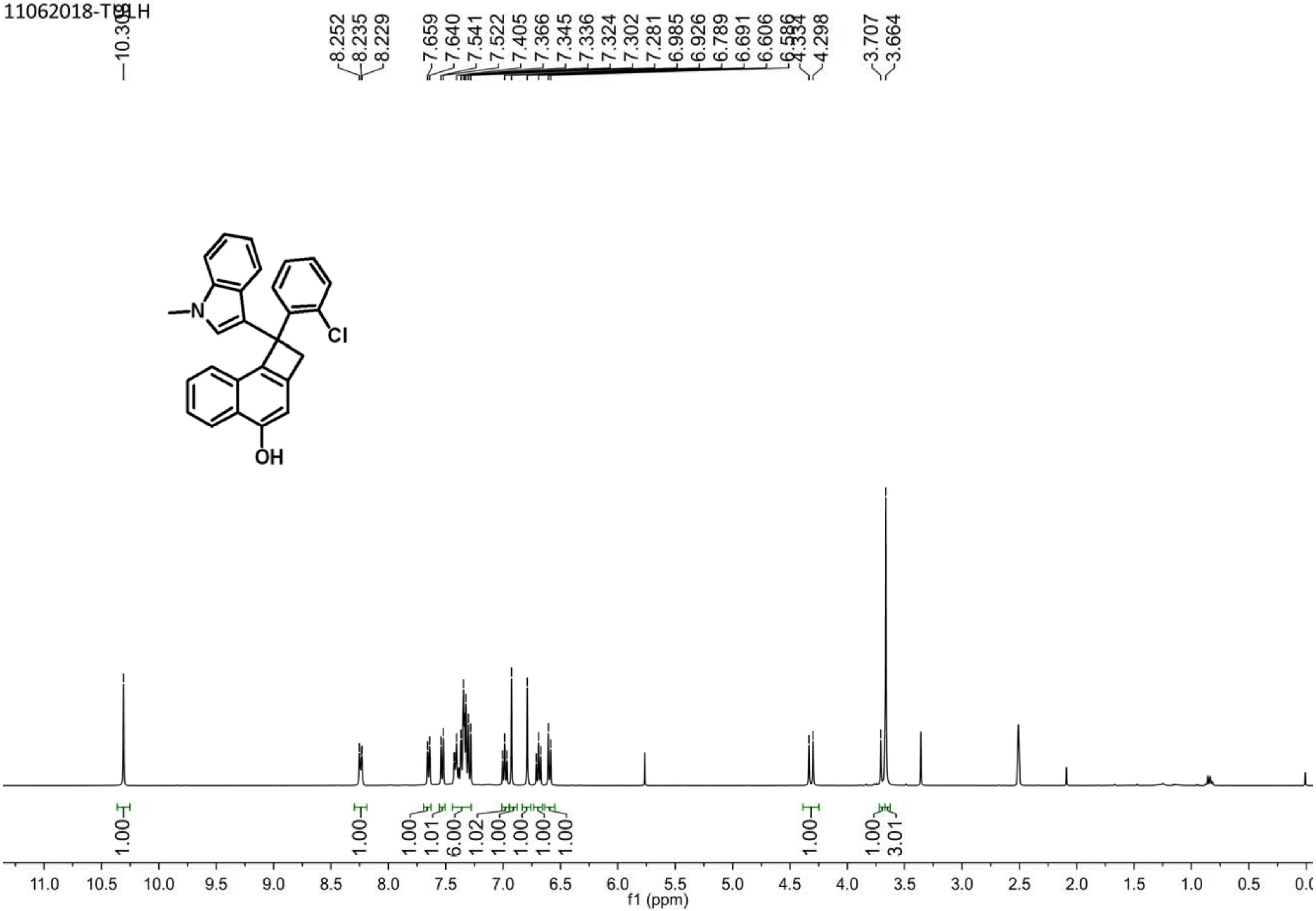

^{1}H NMR Spectrum of Compound 3d

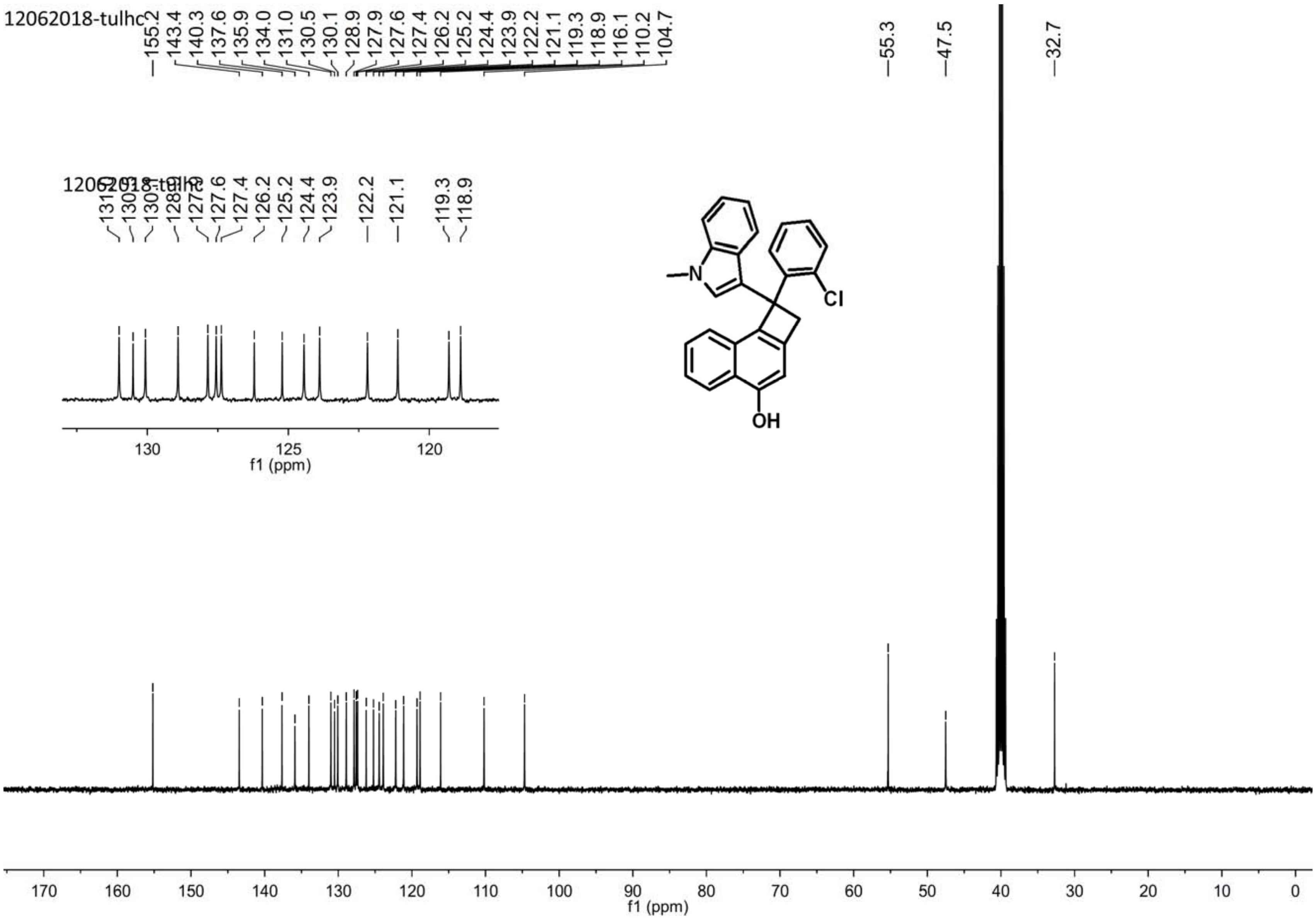

^{13}C NMR Spectrum of Compound 3d

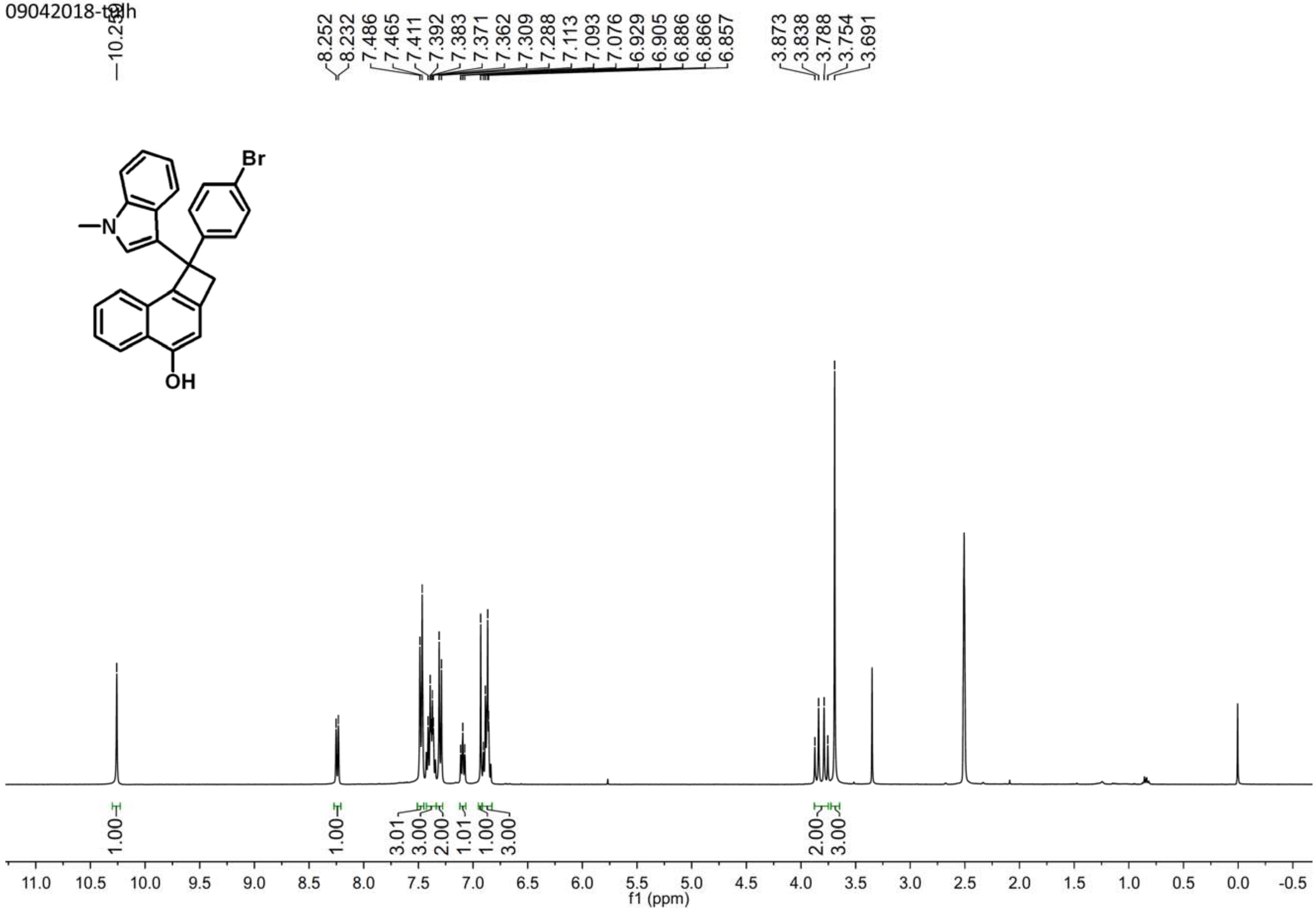

^{1}H NMR Spectrum of Compound 3e

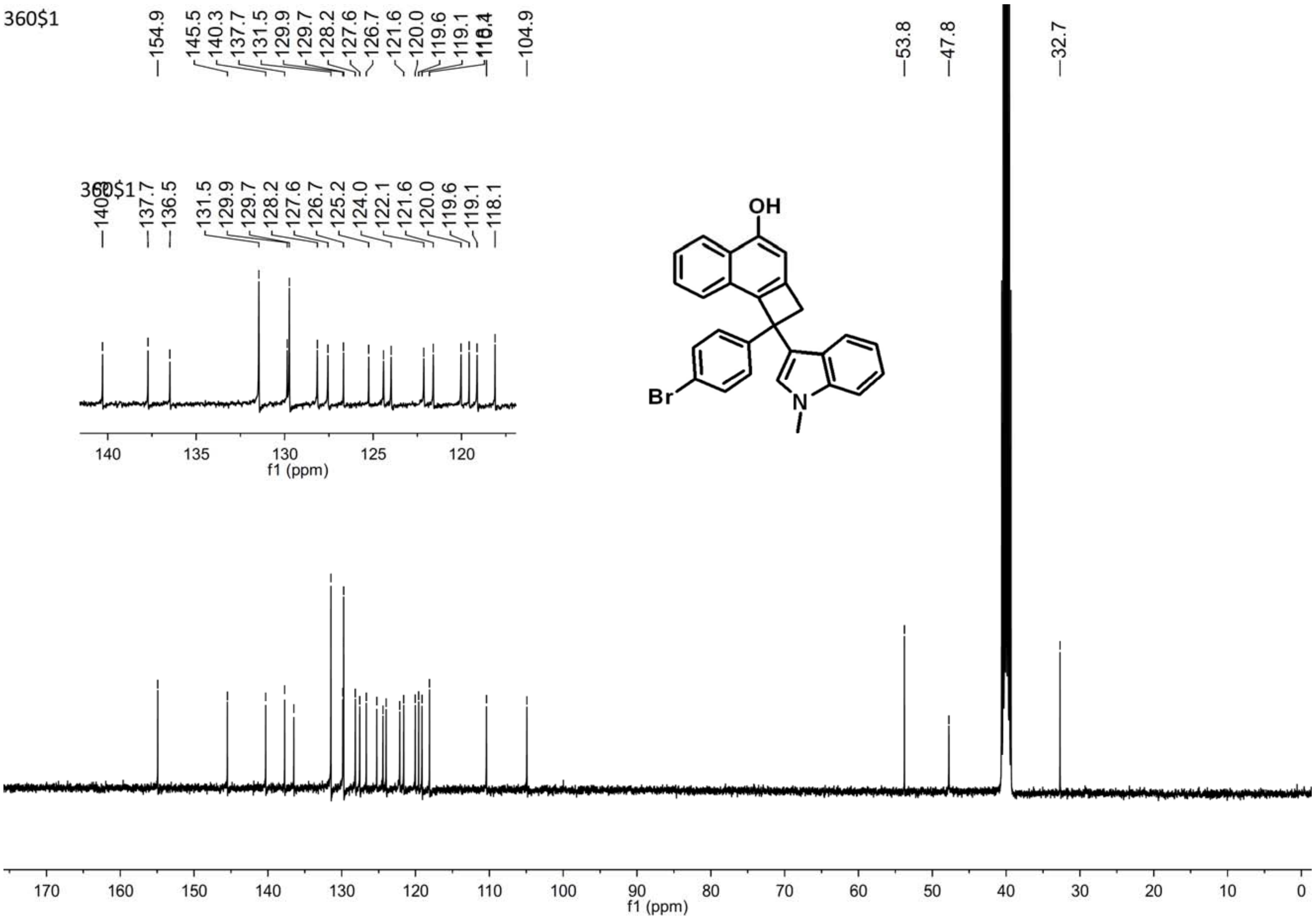

^{13}C NMR Spectrum of Compound 3e

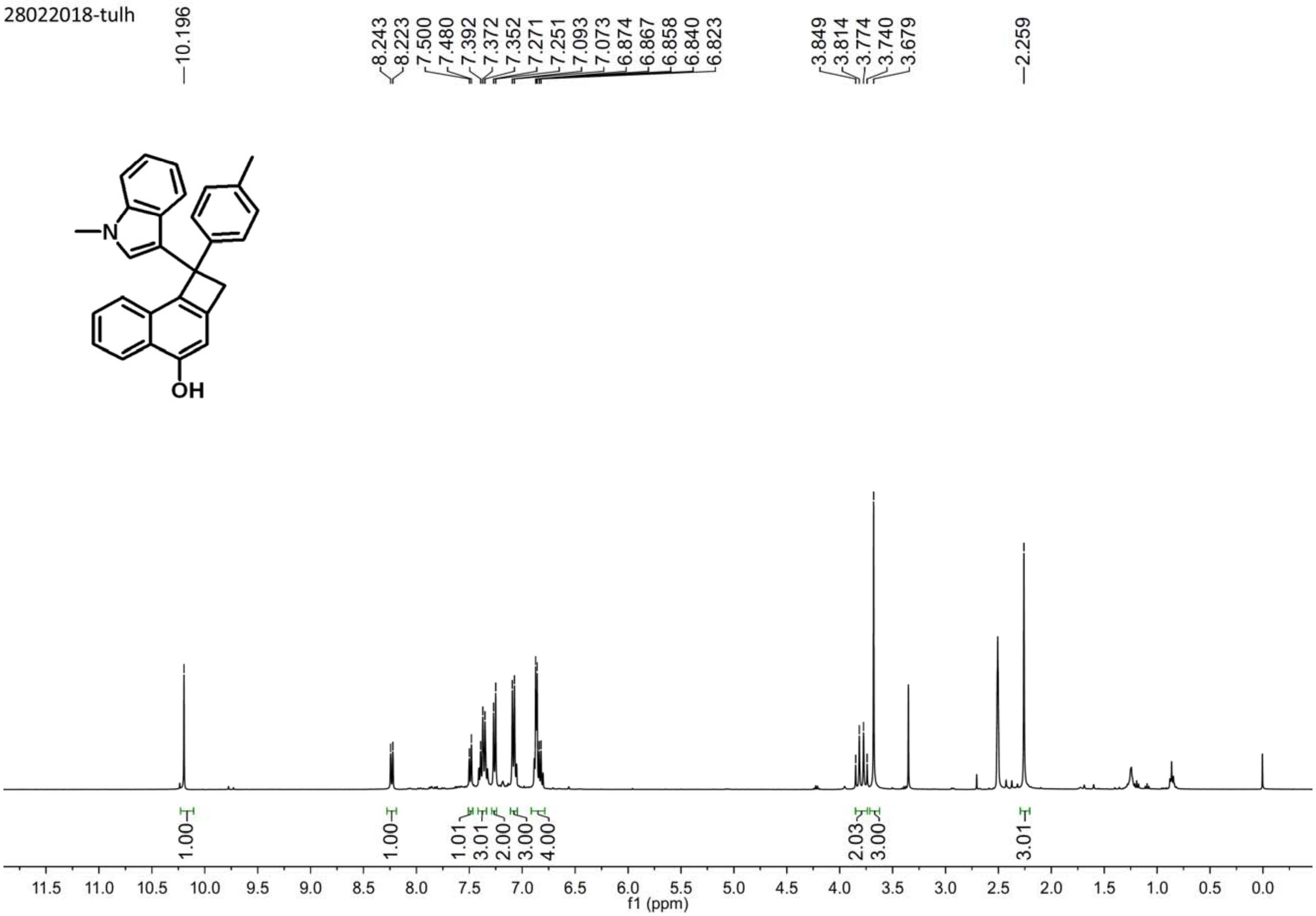

^{1}H NMR Spectrum of Compound 3f

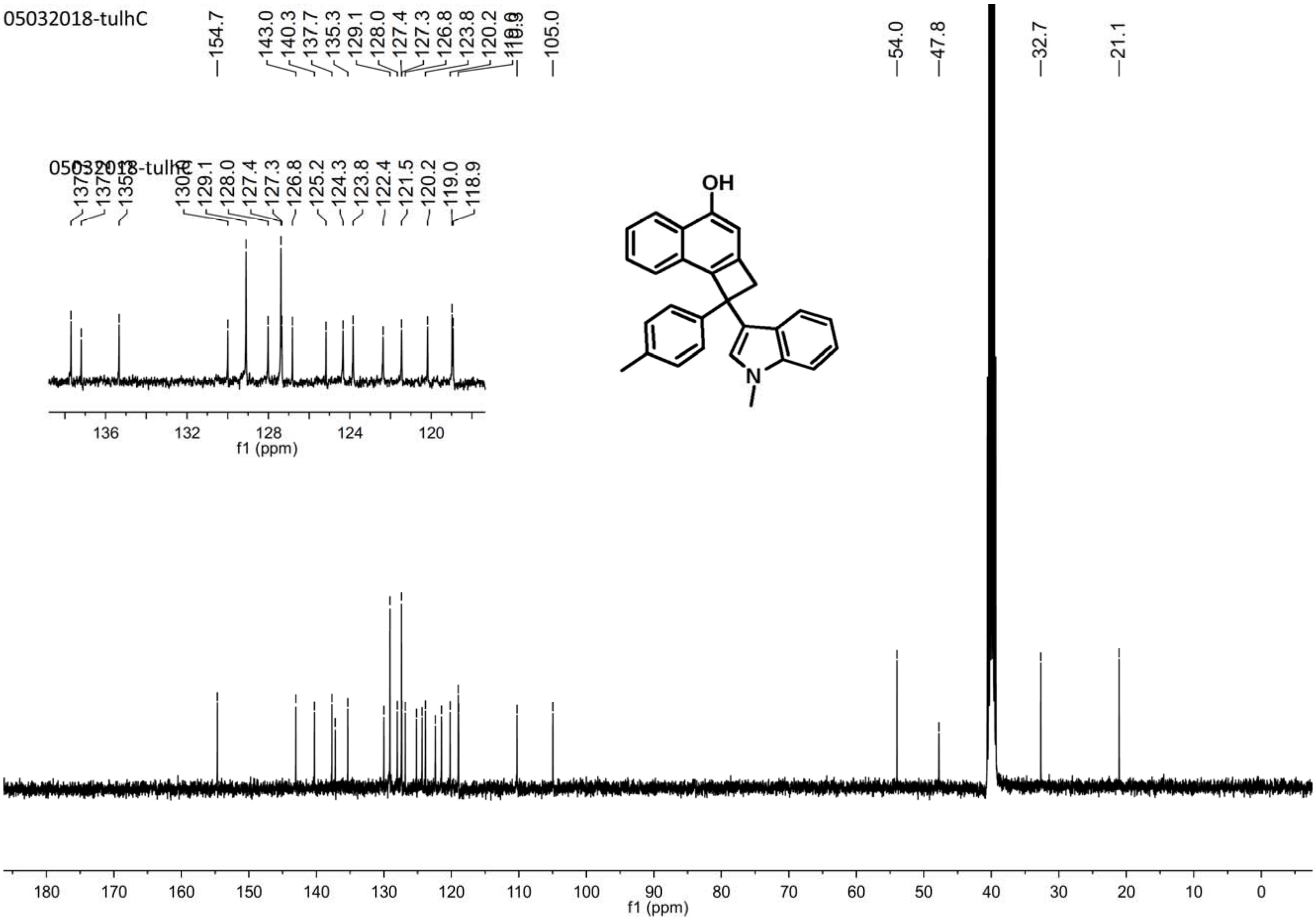

^{13}C NMR Spectrum of Compound 3f

22062018-tu562-1h

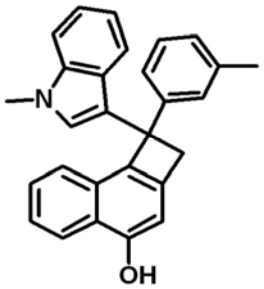

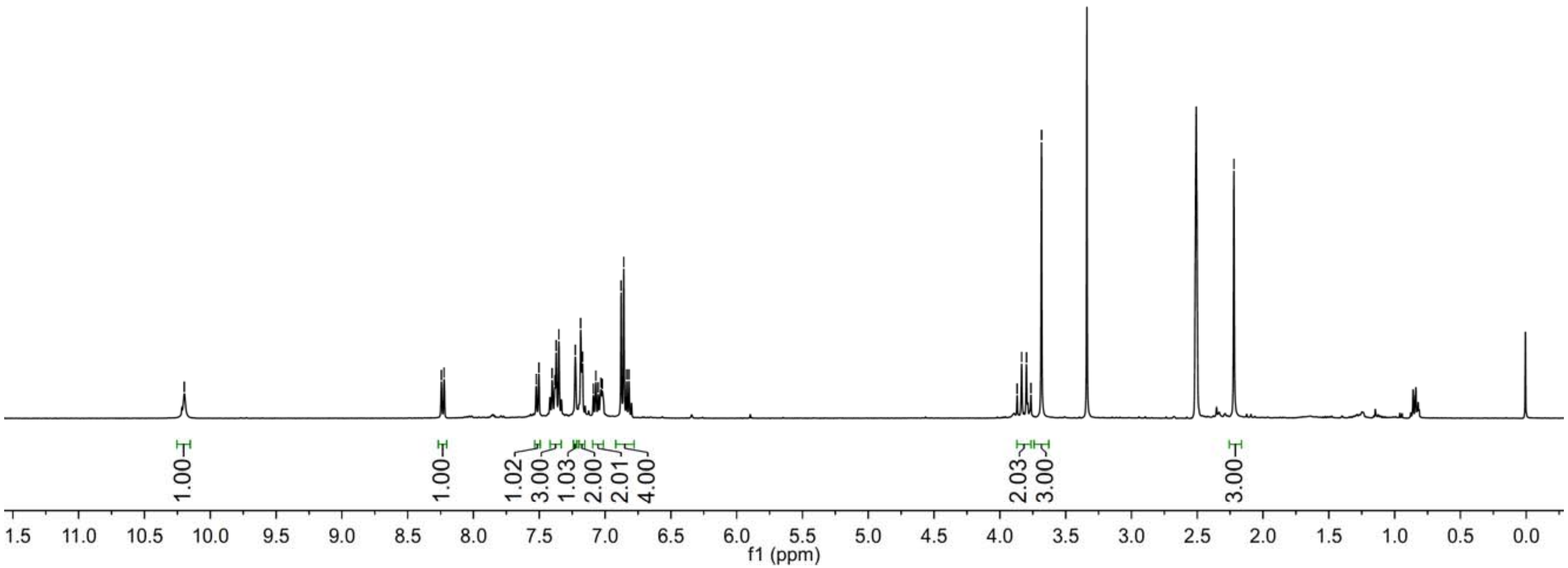

^{1}H NMR Spectrum of Compound 3g

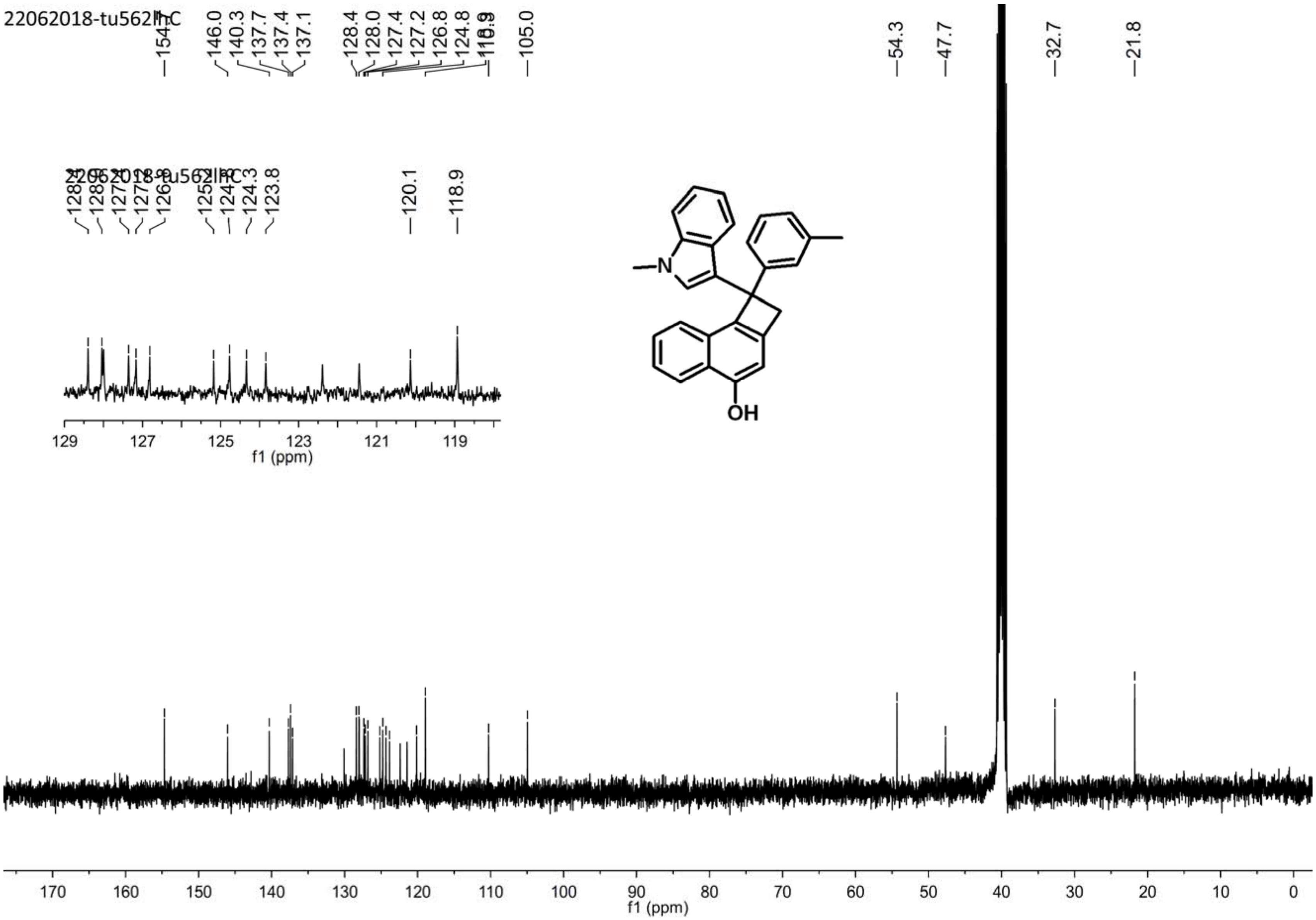

^{13}C NMR Spectrum of Compound 3g

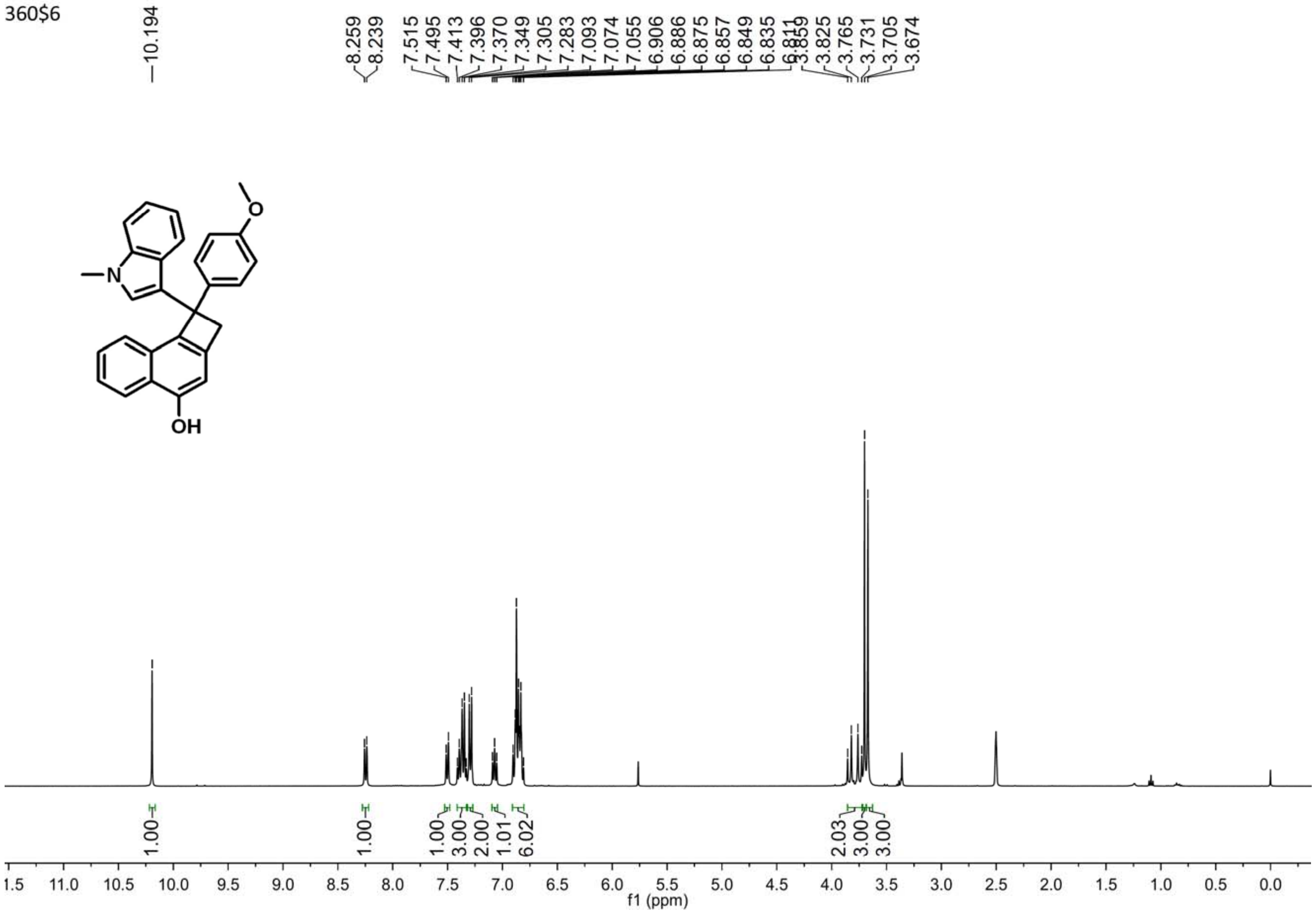

^{1}H NMR Spectrum of Compound 3h

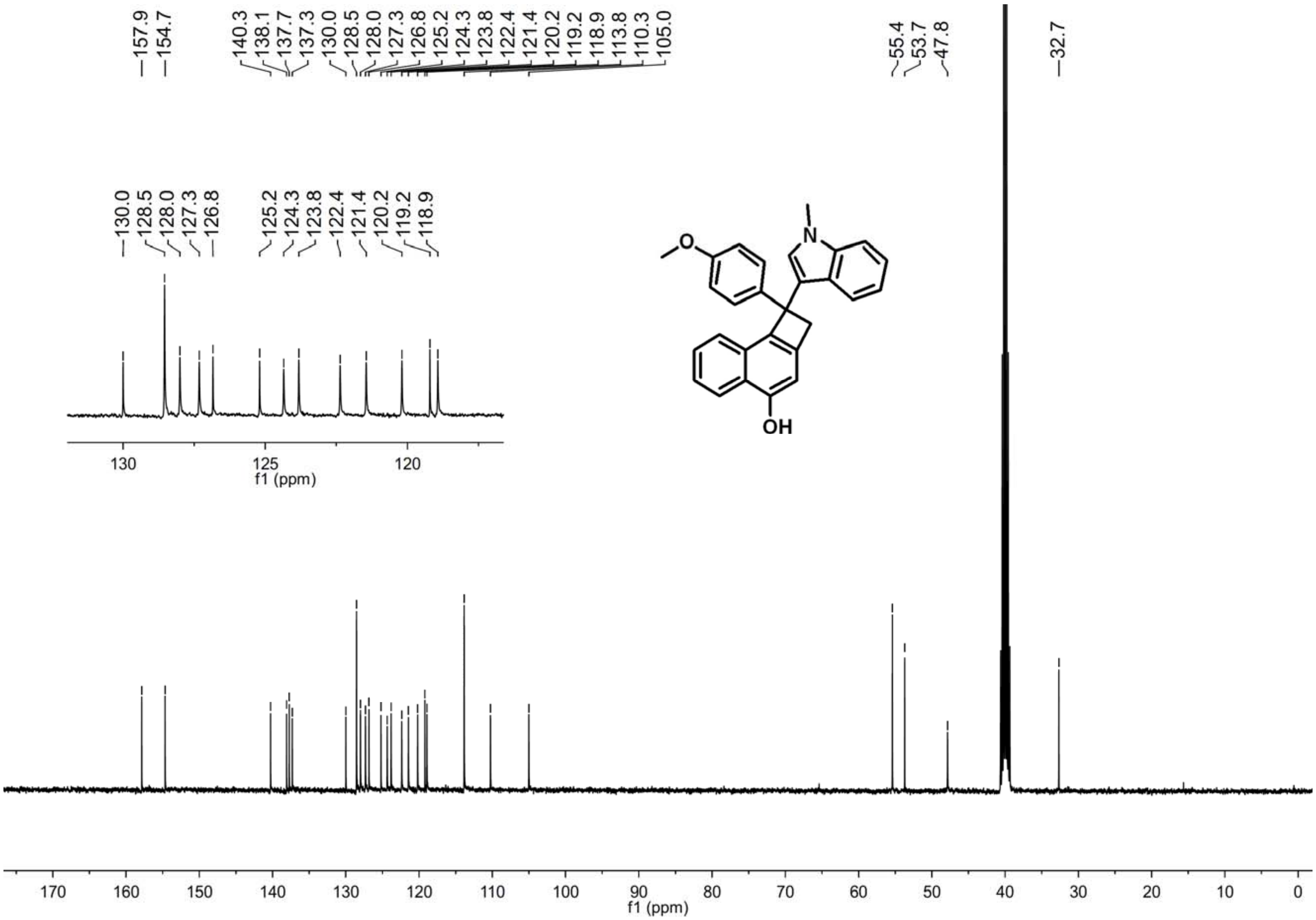

^{13}C NMR Spectrum of Compound 3h

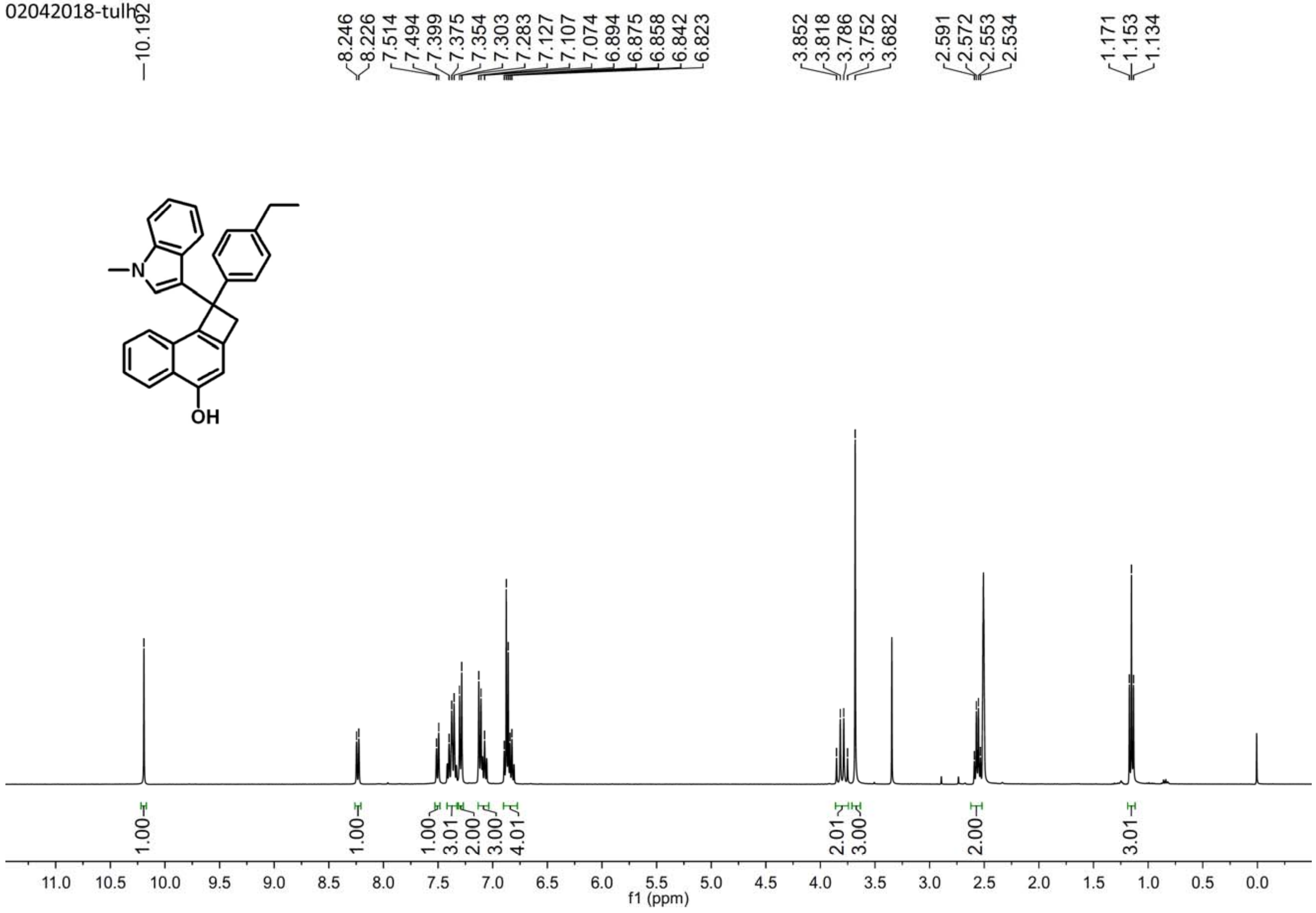

^{1}H NMR Spectrum of Compound 3i

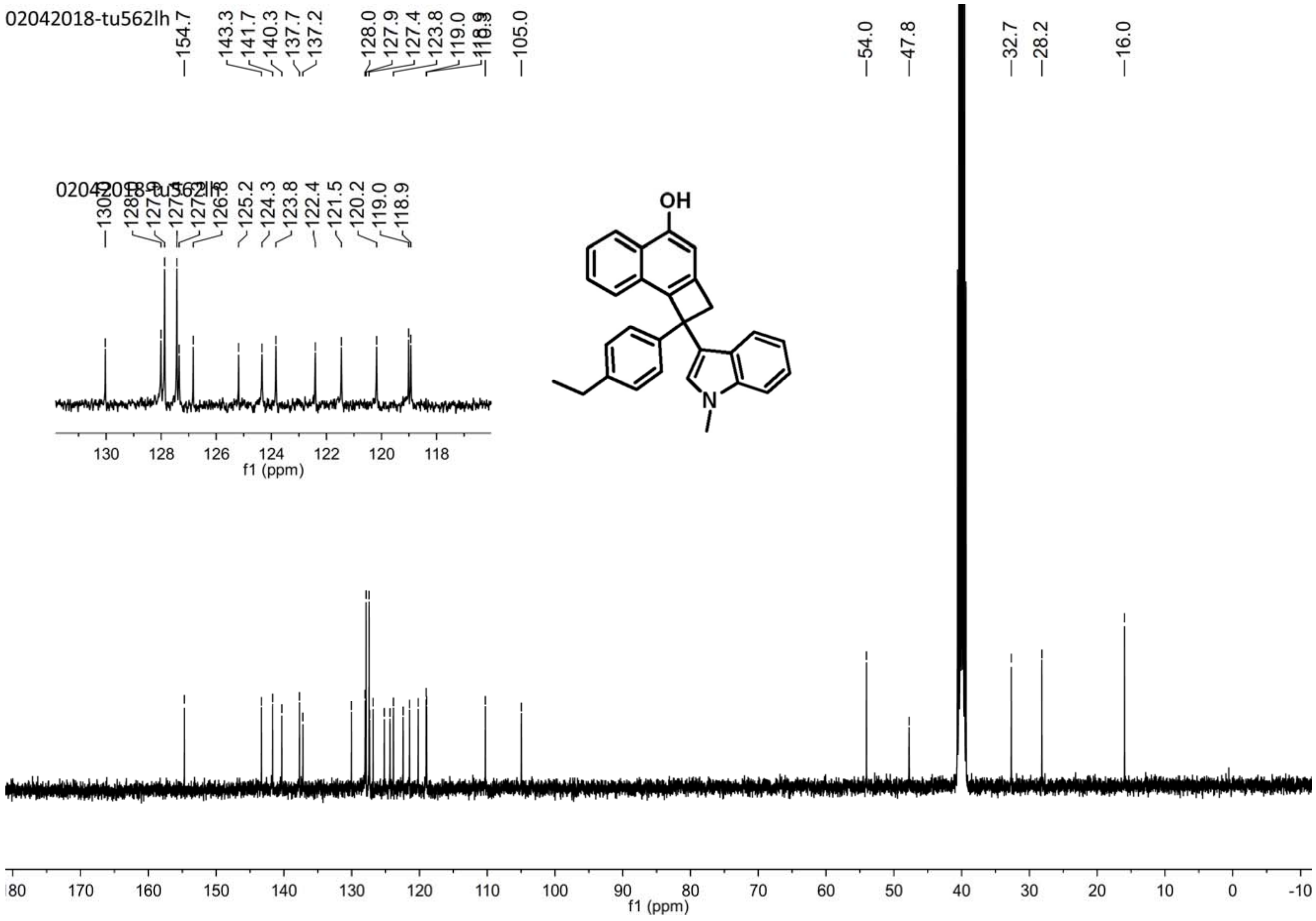

^{13}C NMR Spectrum of Compound 3i

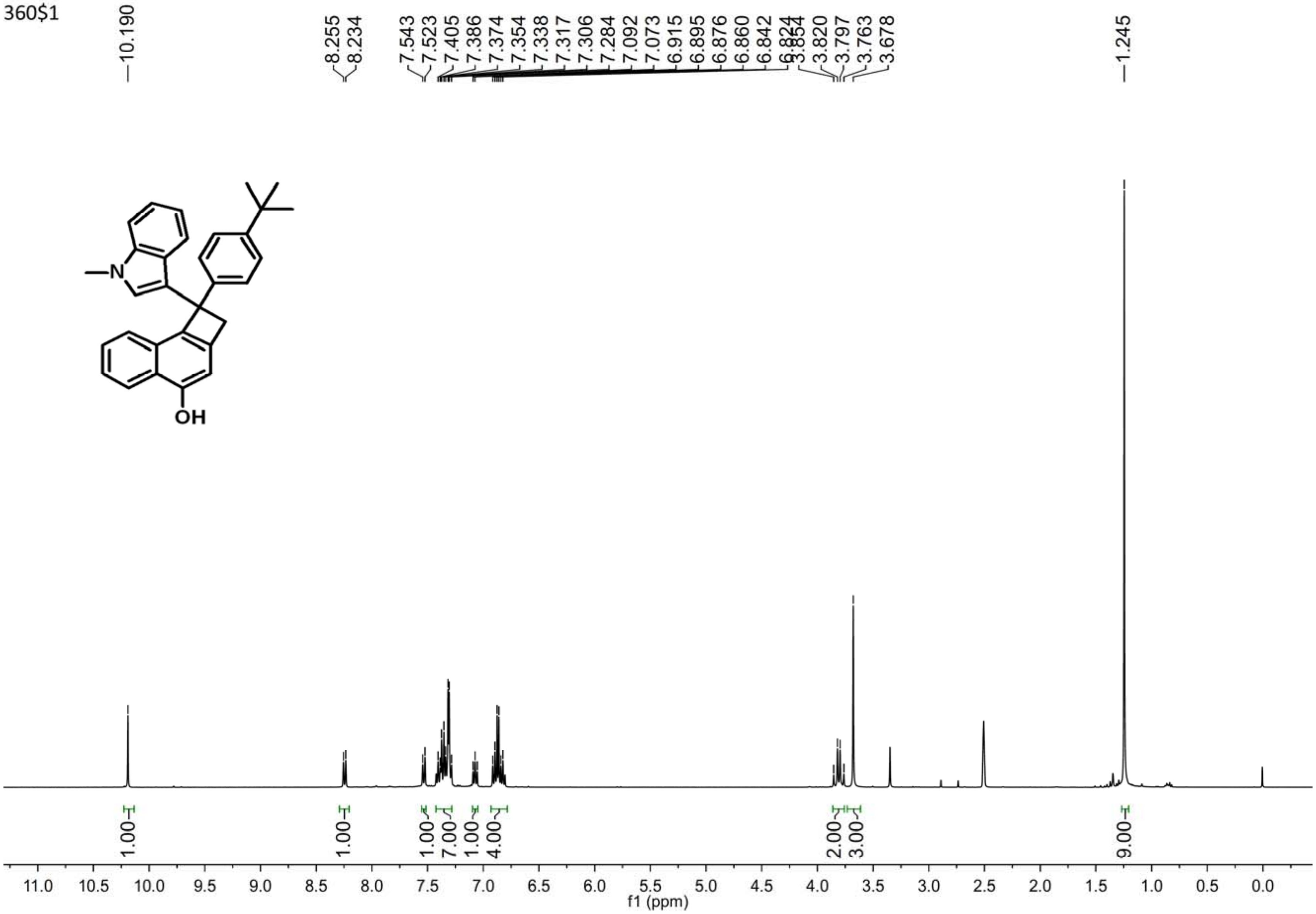

^{1}H NMR Spectrum of Compound 3j

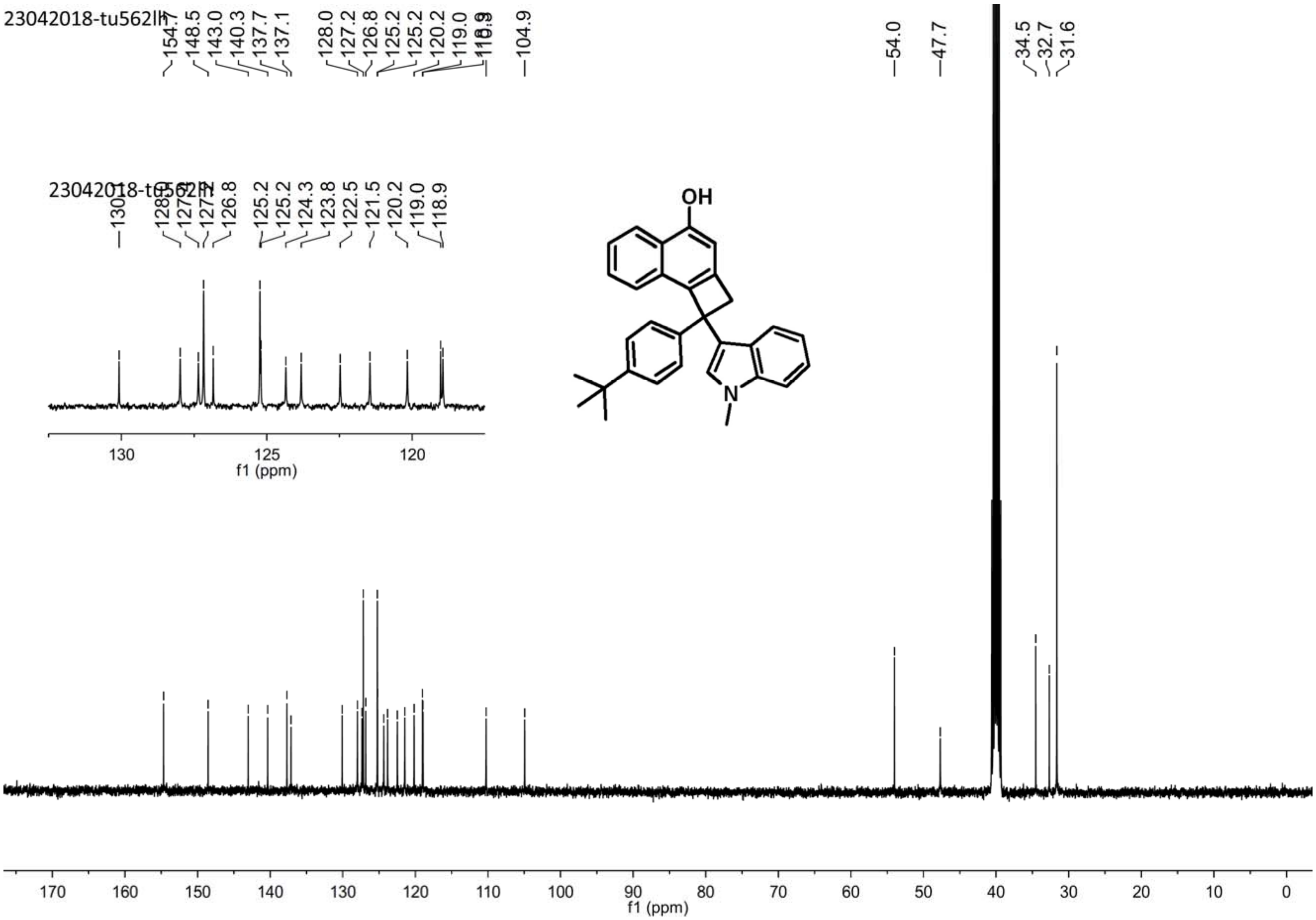

^{13}C NMR Spectrum of Compound 3j

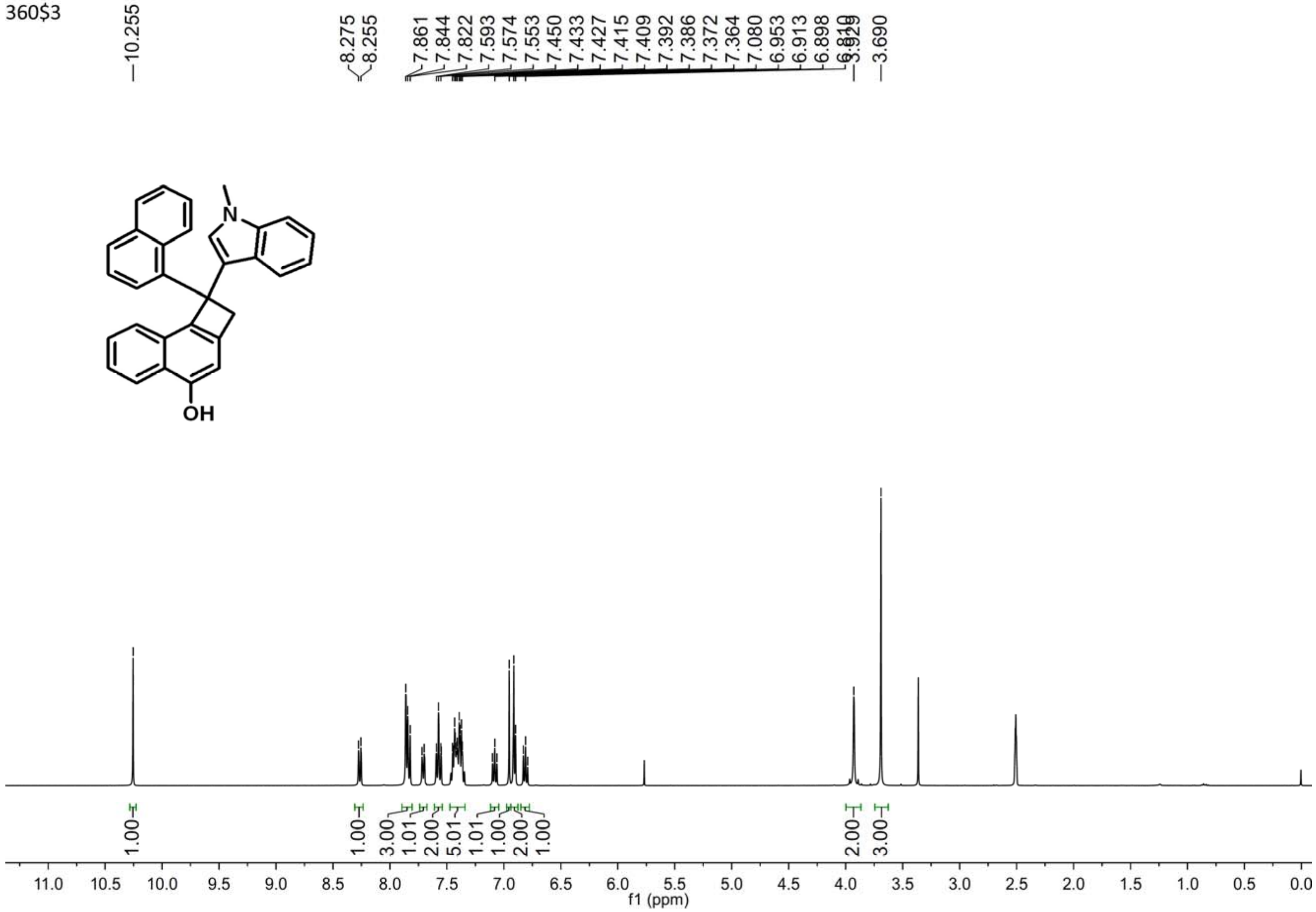

^{1}H NMR Spectrum of Compound 3k

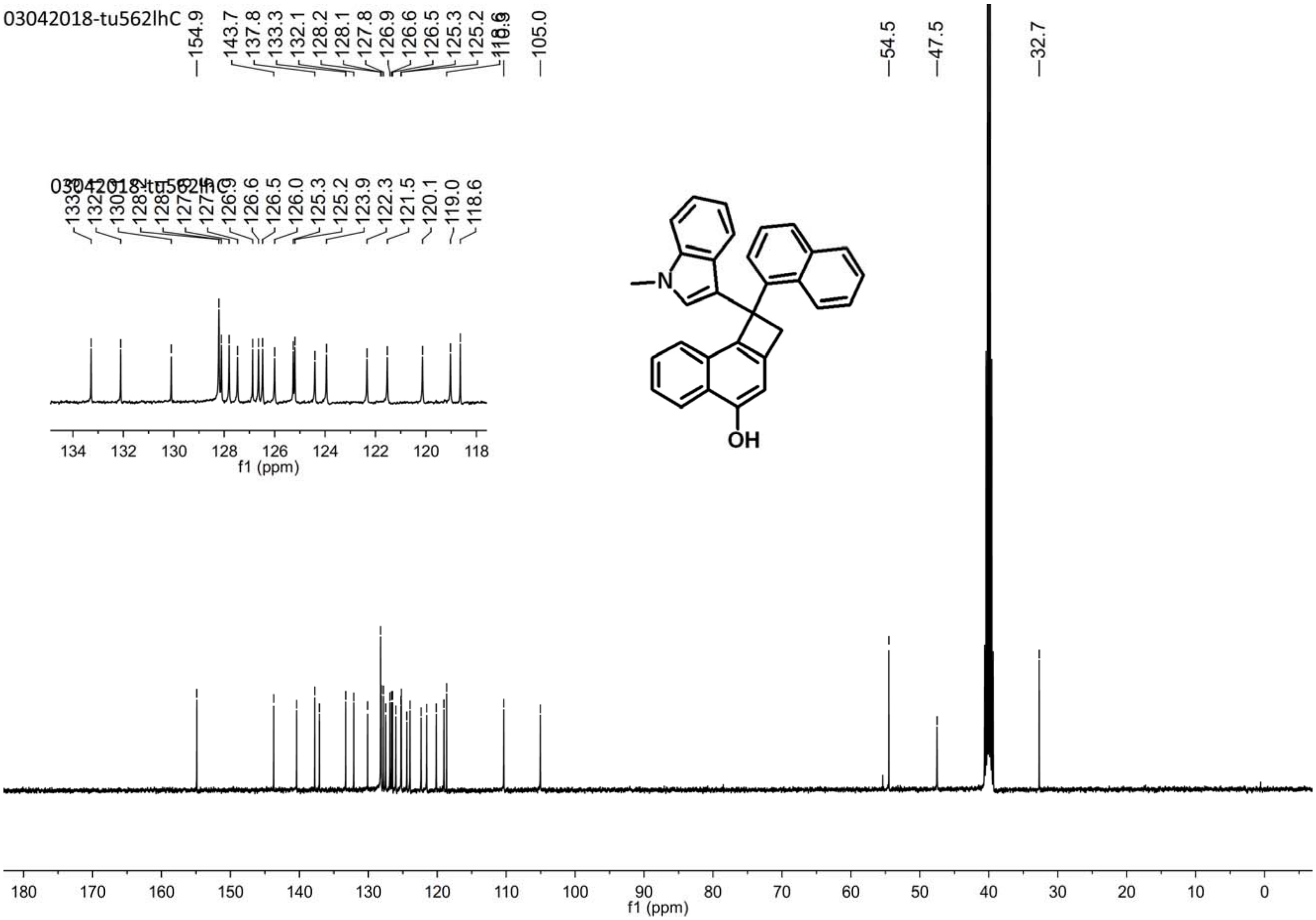

^{13}C NMR Spectrum of Compound 3k

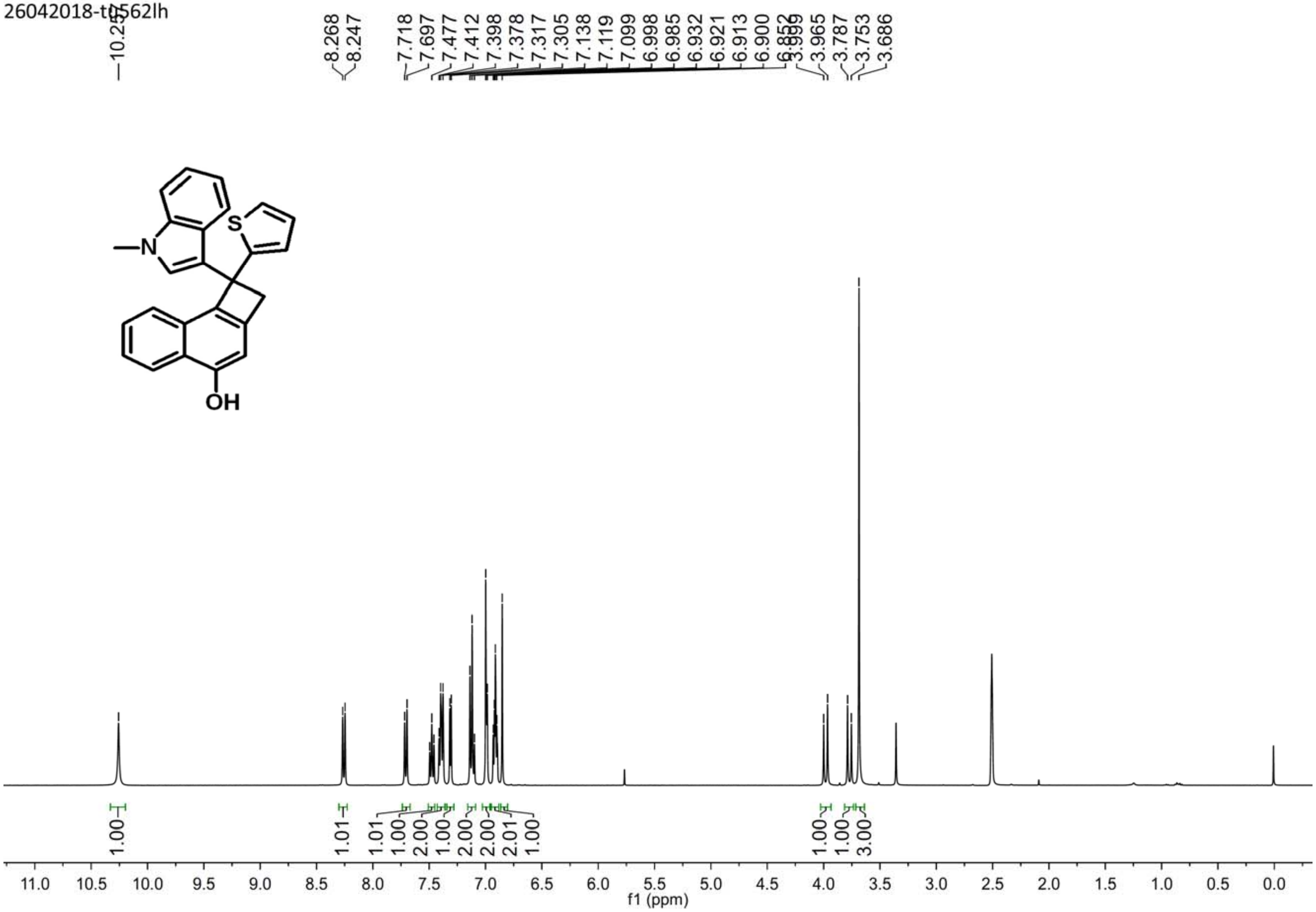

^{1}H NMR Spectrum of Compound 3l

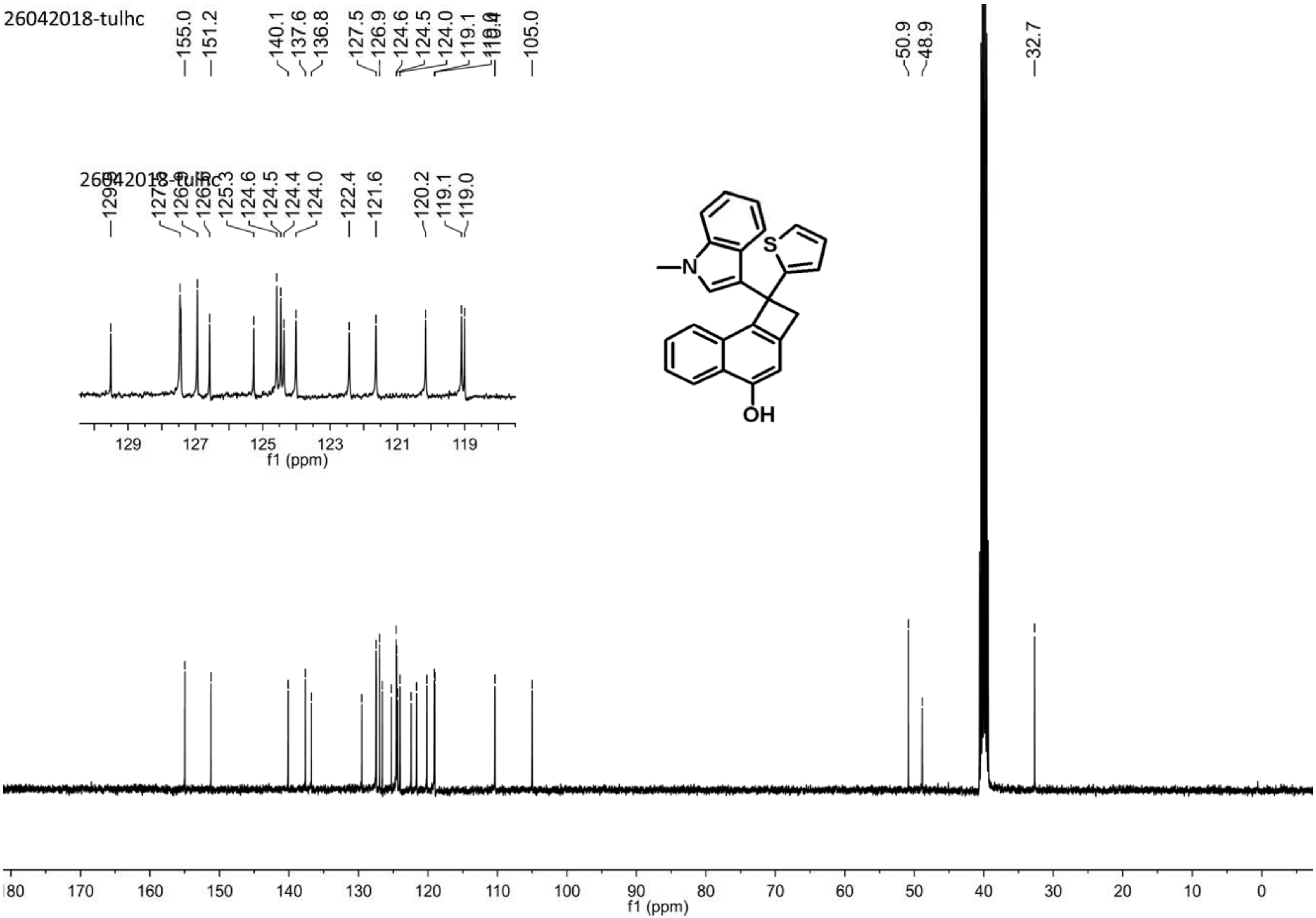

^{13}C NMR Spectrum of Compound 3l

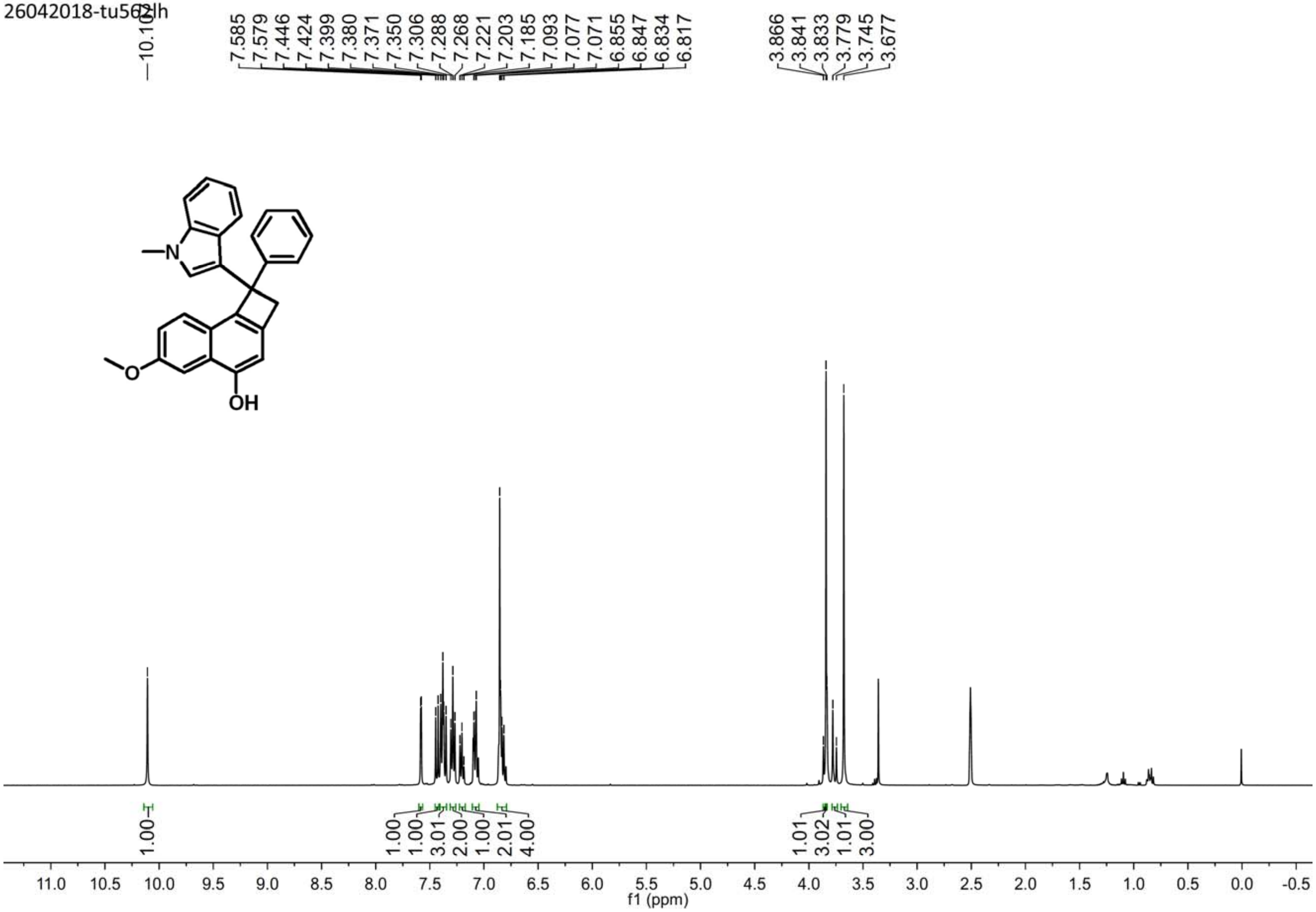

^{1}H NMR Spectrum of Compound 3m

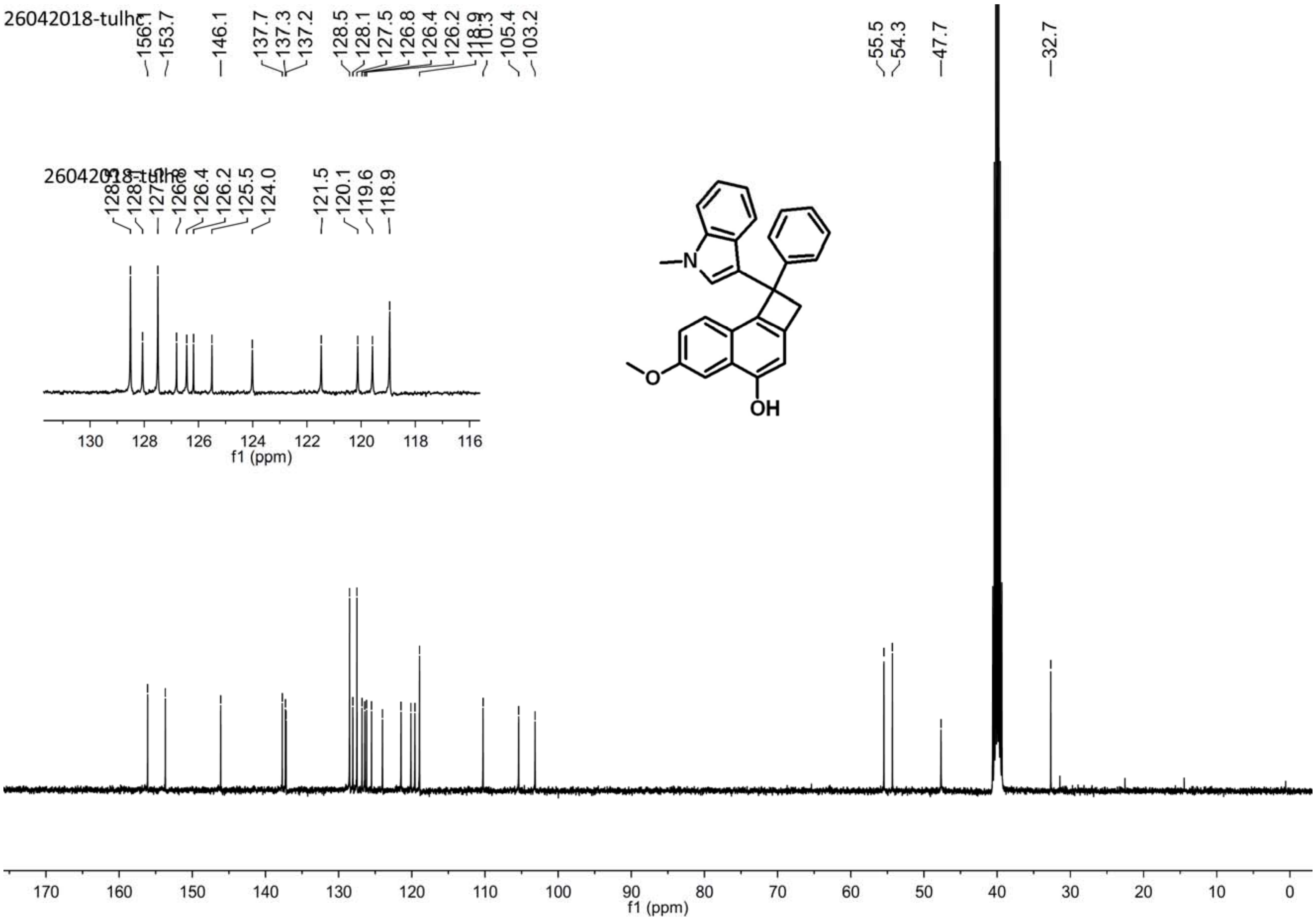

^{13}C NMR Spectrum of Compound 3m

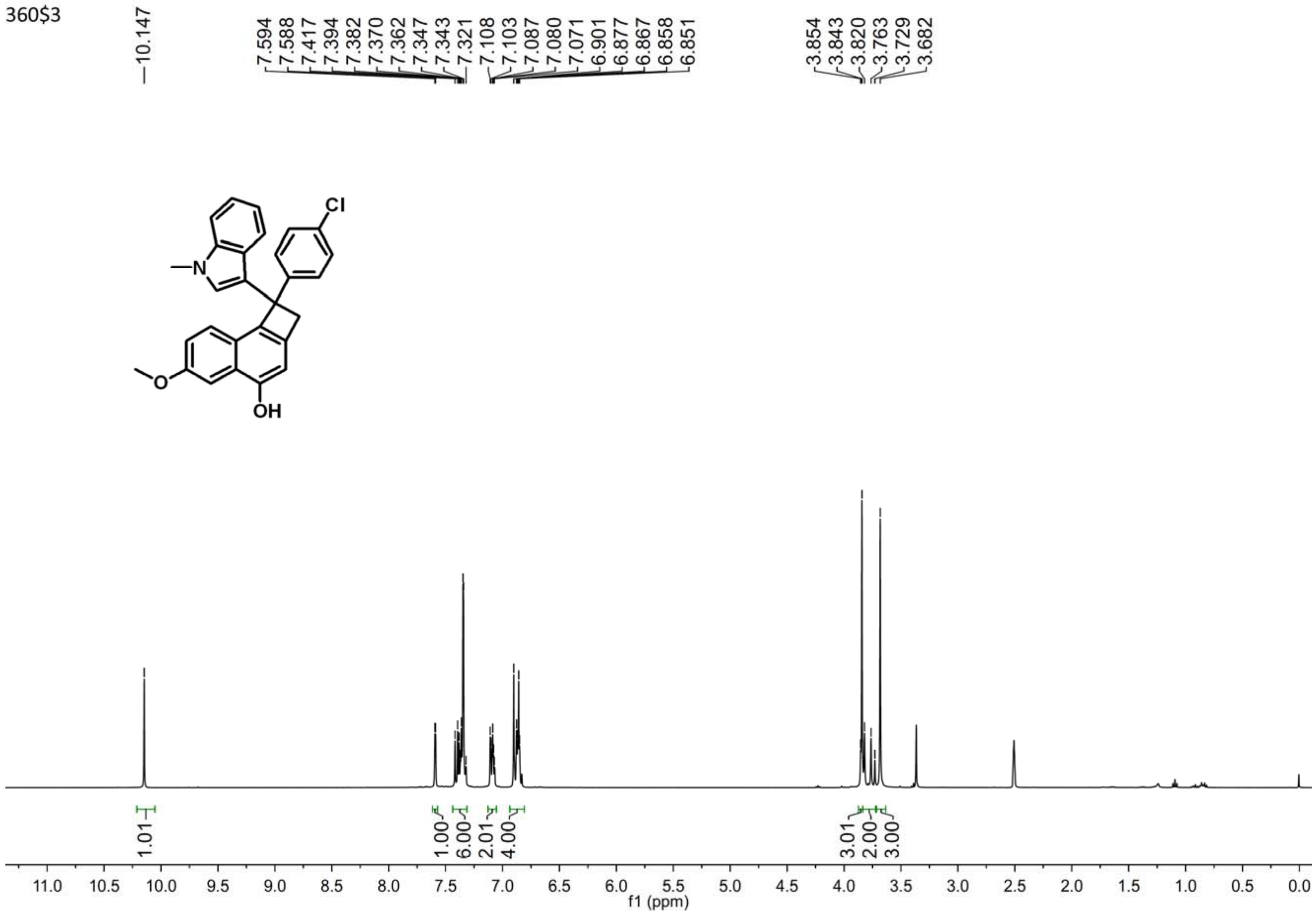

^{1}H NMR Spectrum of Compound 3n

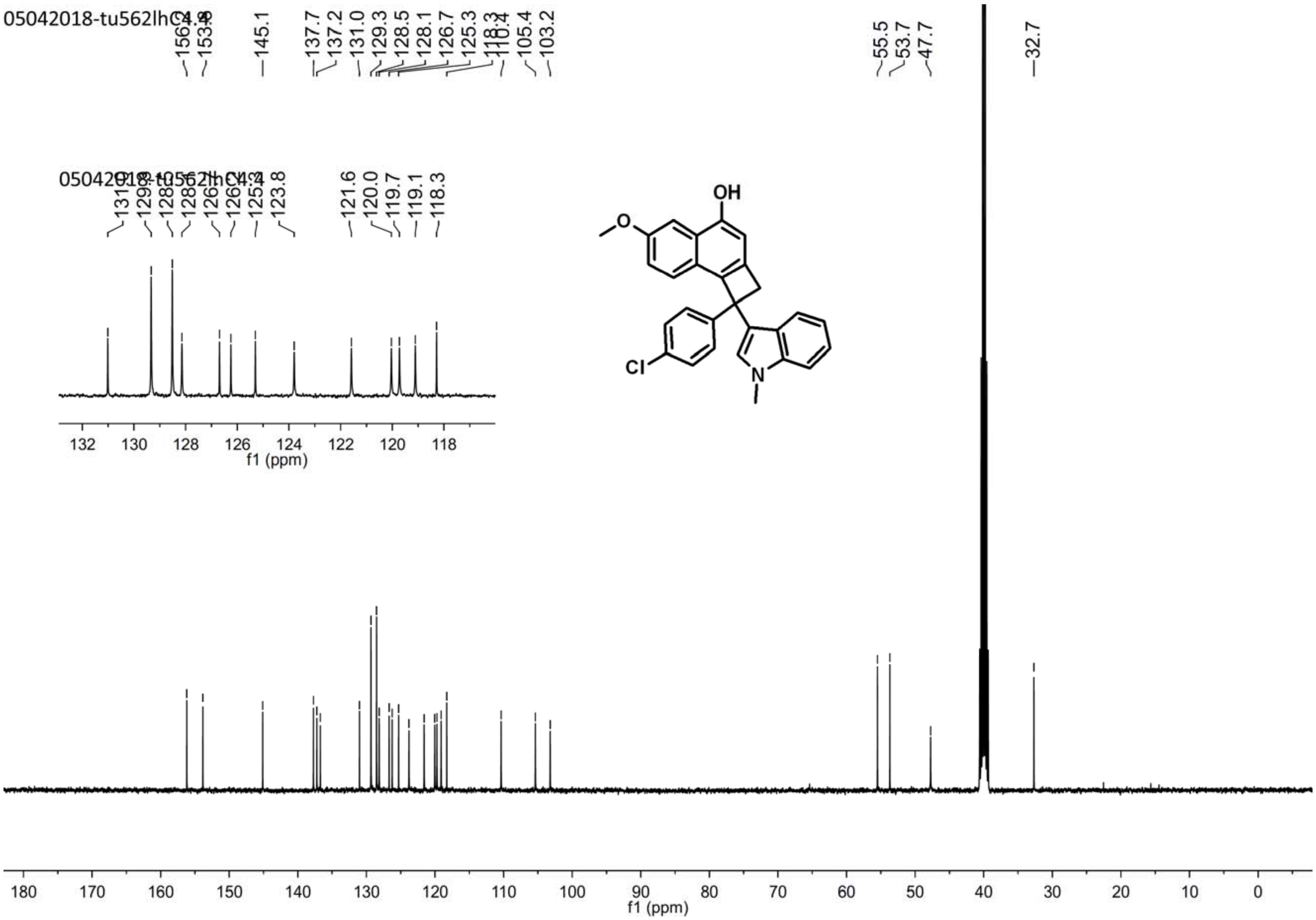

^{13}C NMR Spectrum of Compound 3n

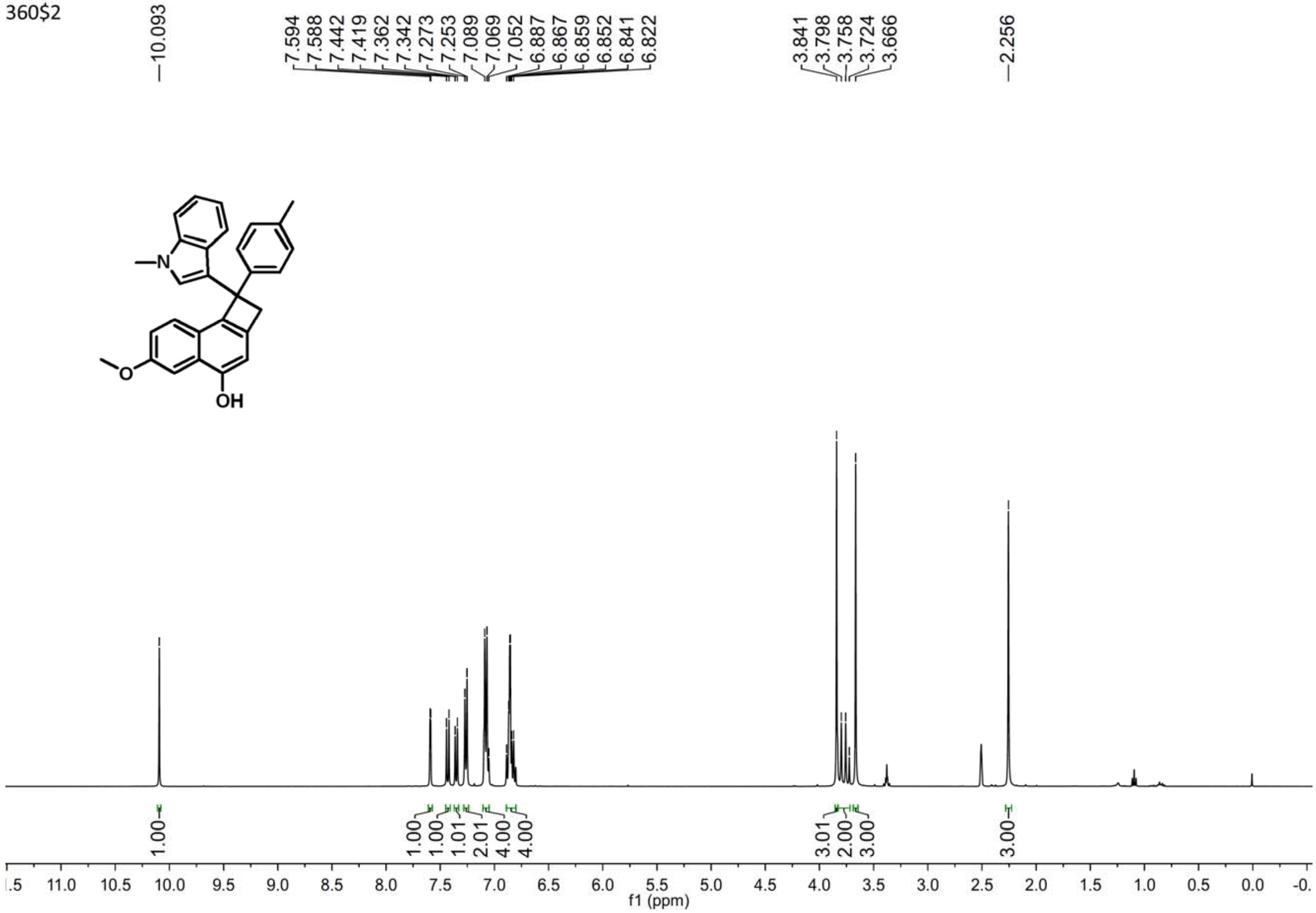

^{1}H NMR Spectrum of Compound 3o

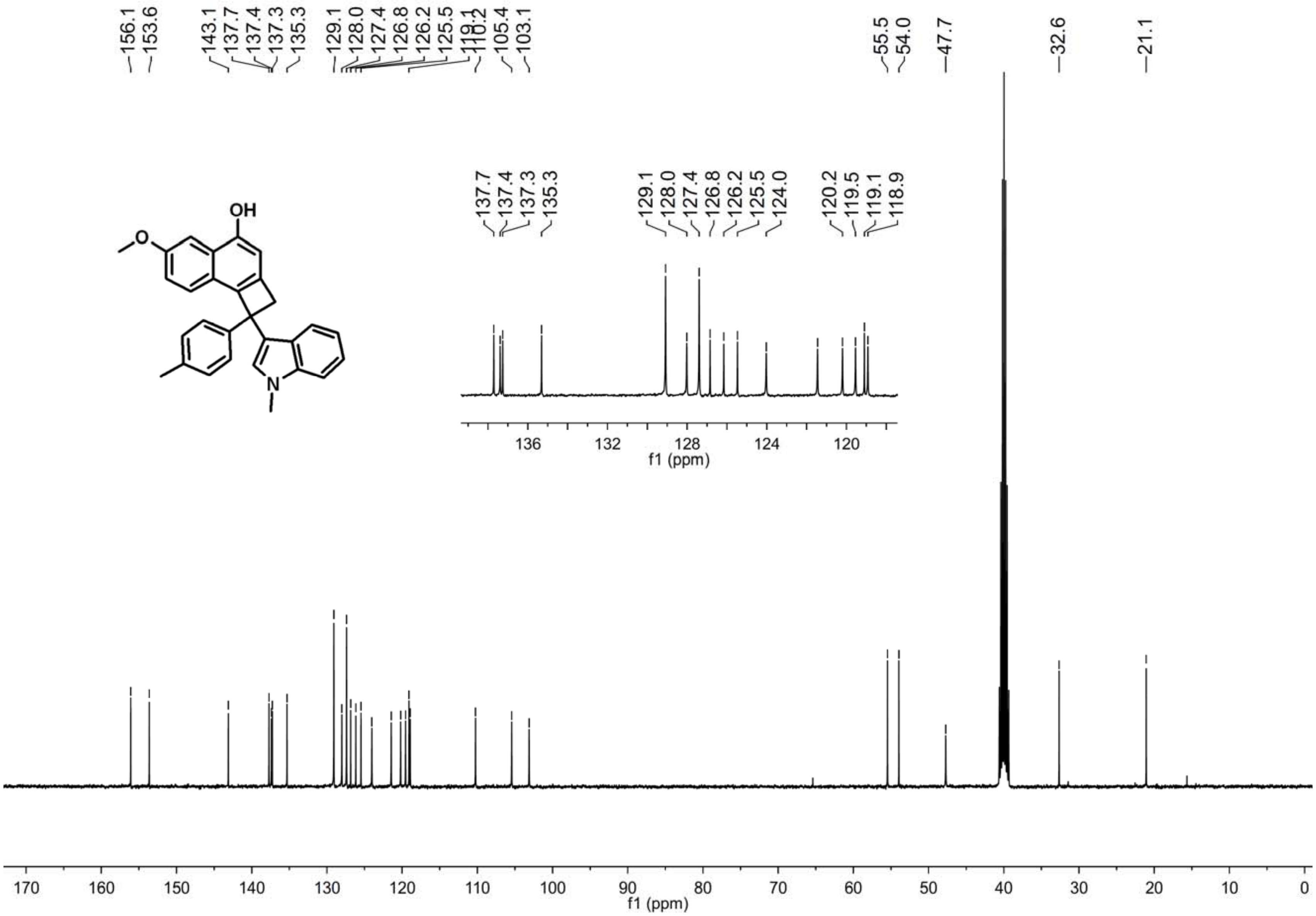

^{13}C NMR Spectrum of Compound 3o

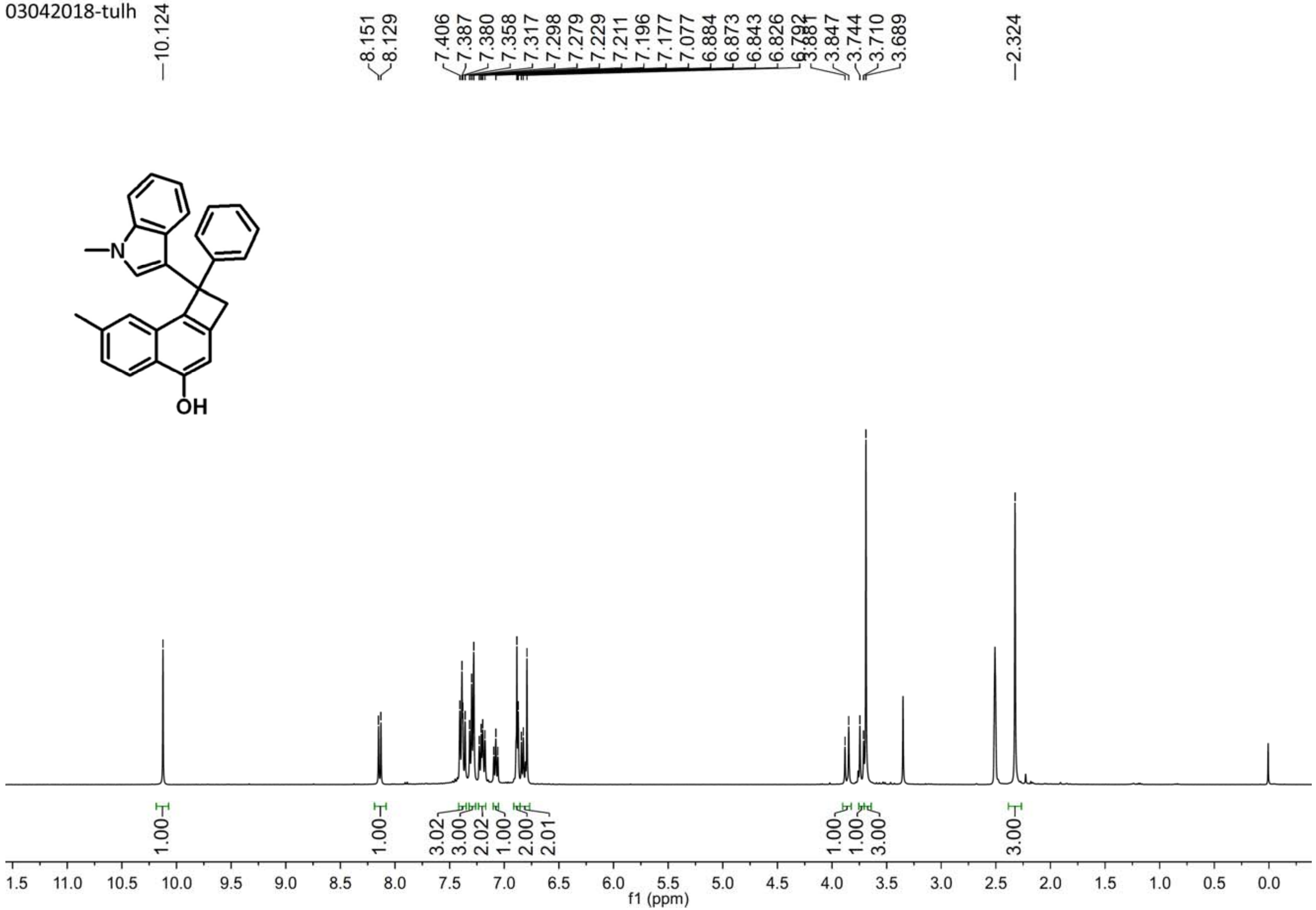

^{1}H NMR Spectrum of Compound 3p

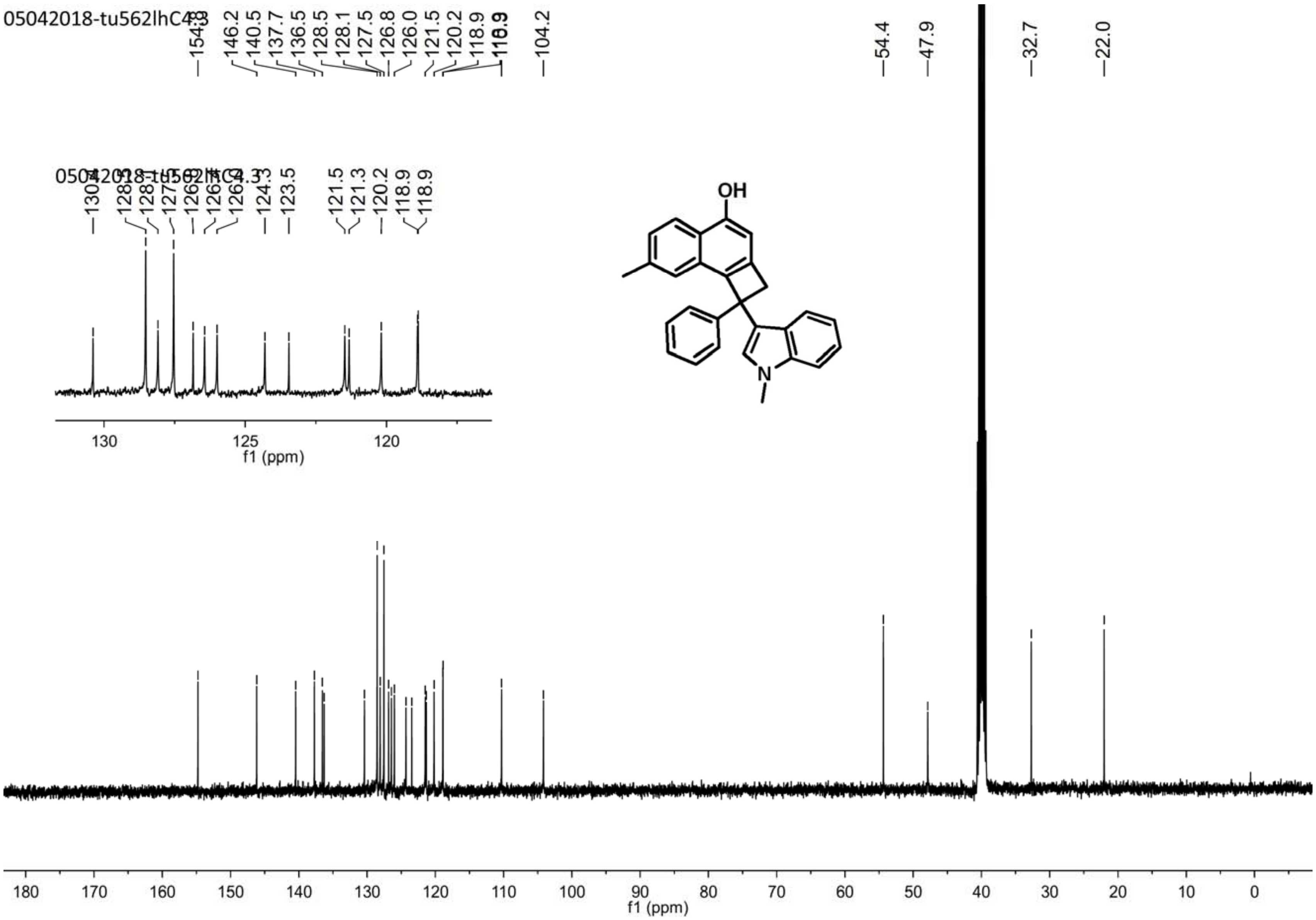

^{13}C NMR Spectrum of Compound 3p

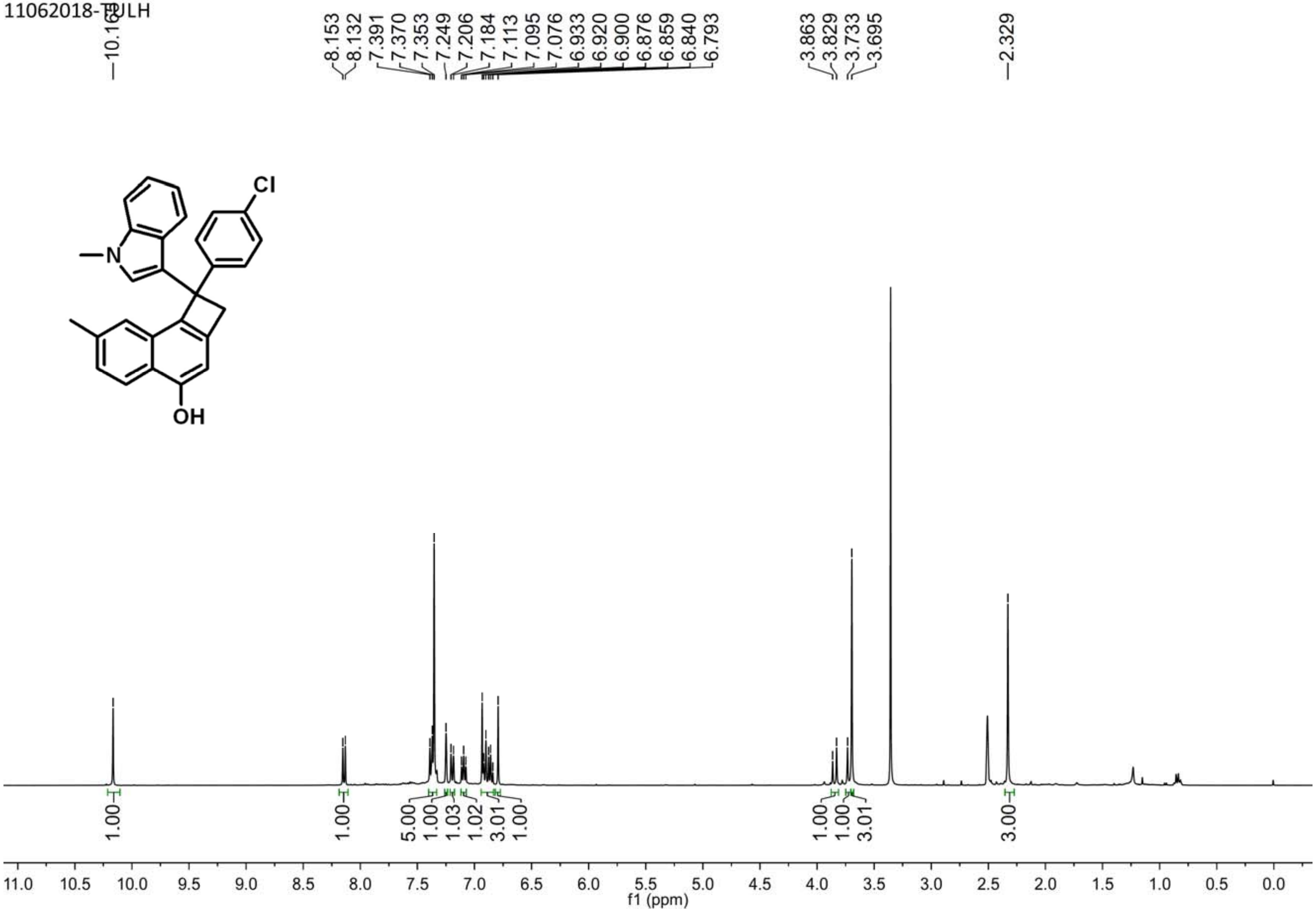

^{1}H NMR Spectrum of Compound 3q

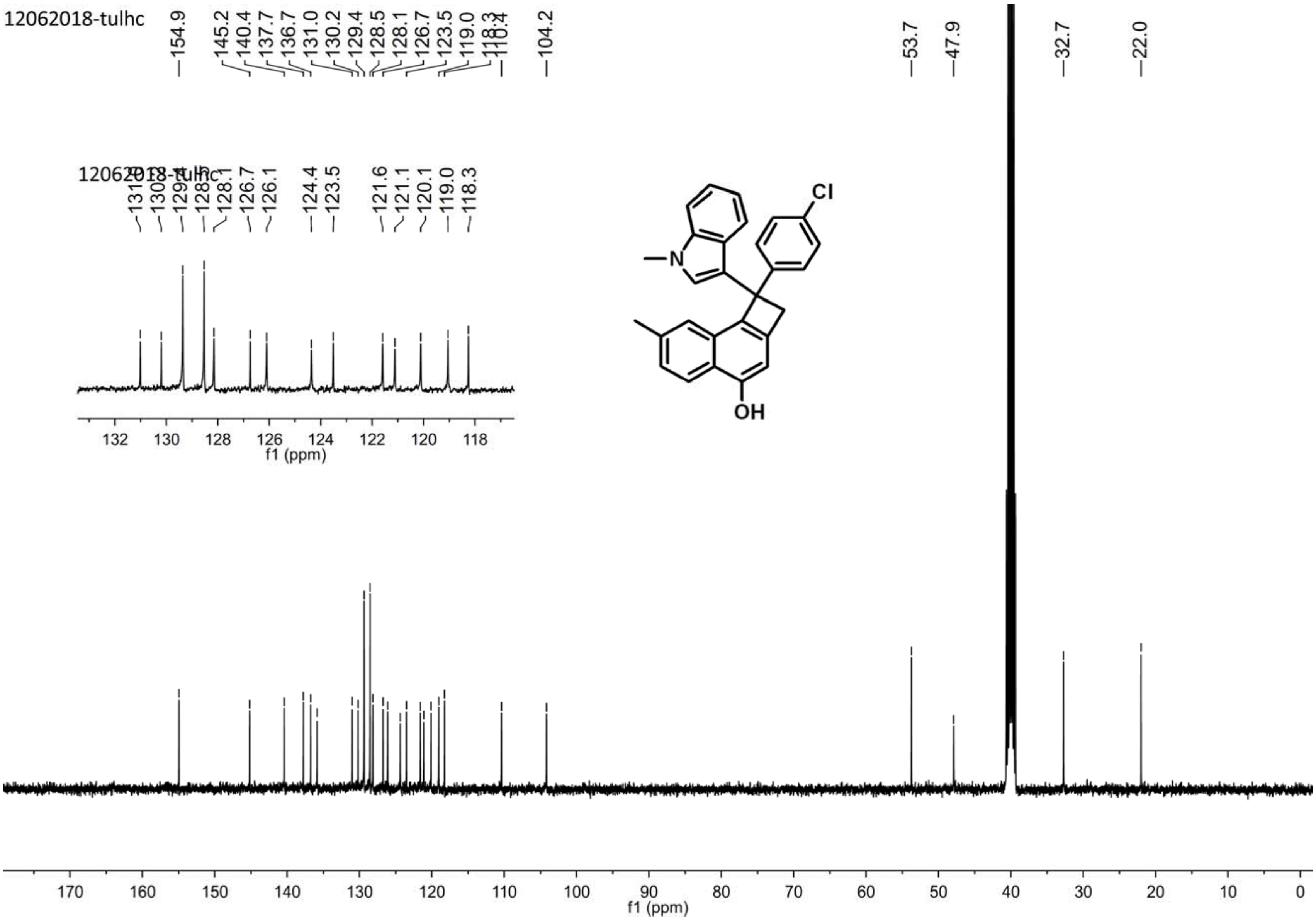

^{13}C NMR Spectrum of Compound 3q

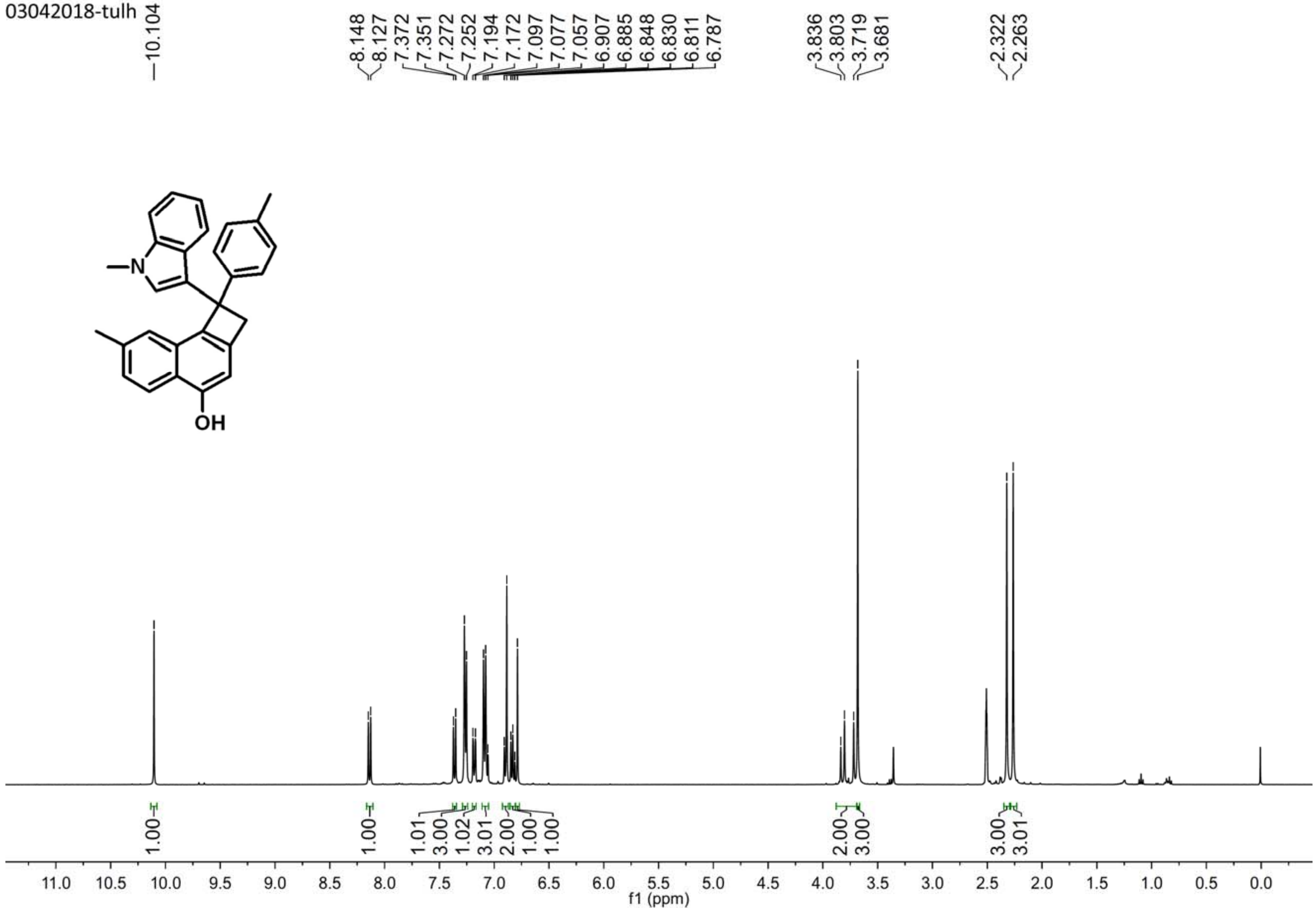

^{1}H NMR Spectrum of Compound 3r

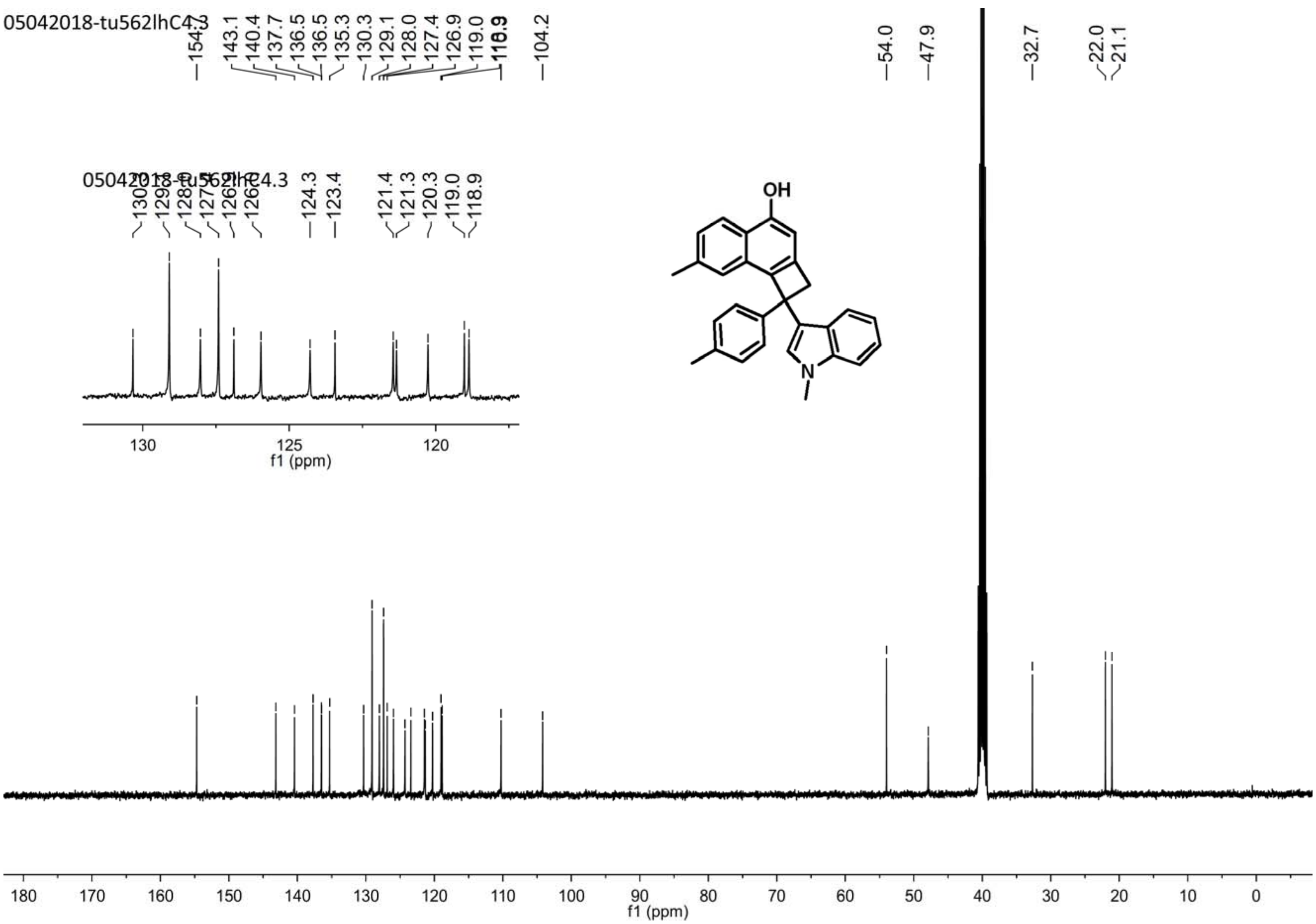

^{13}C NMR Spectrum of Compound 3r

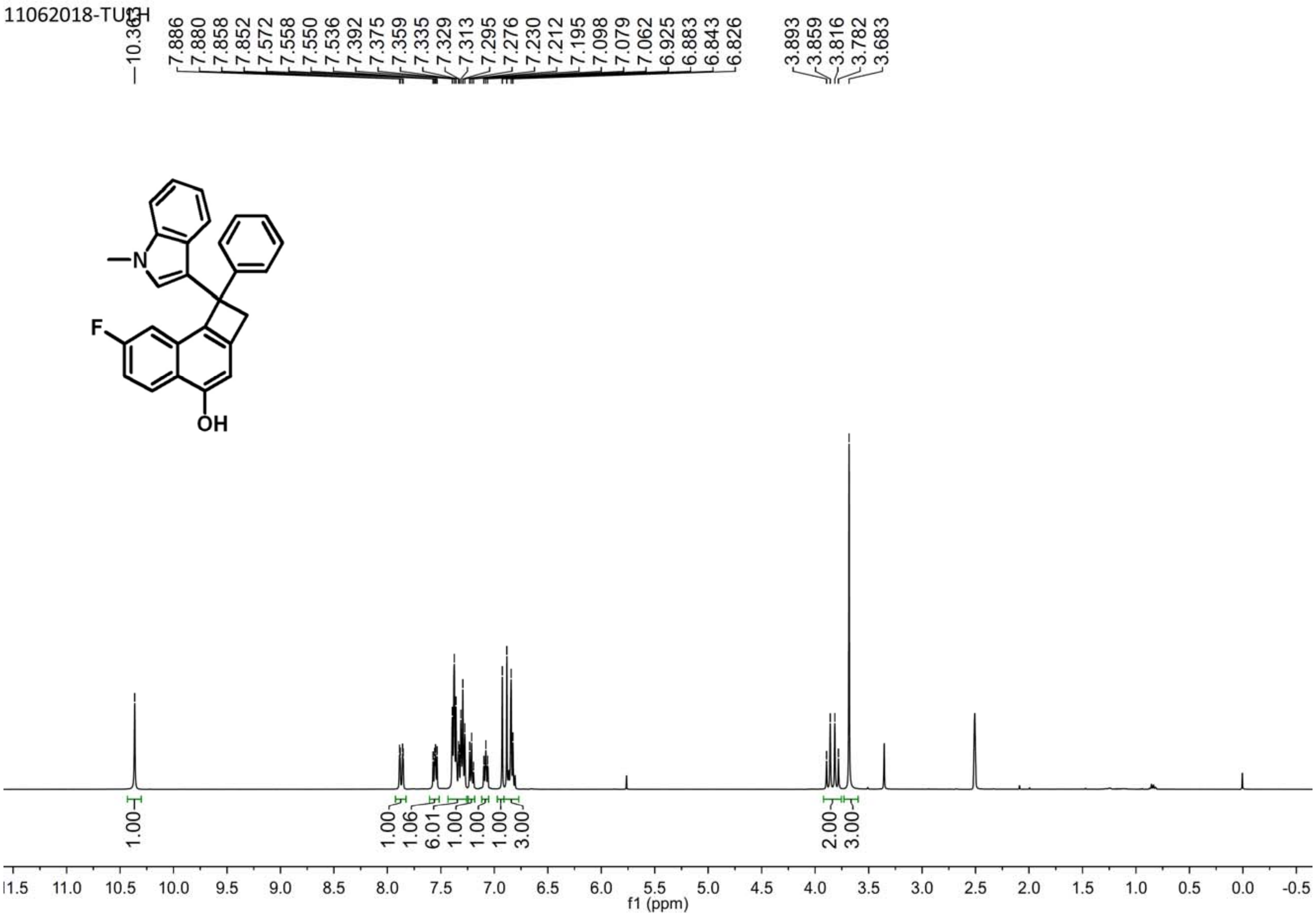

^{1}H NMR Spectrum of Compound 3s

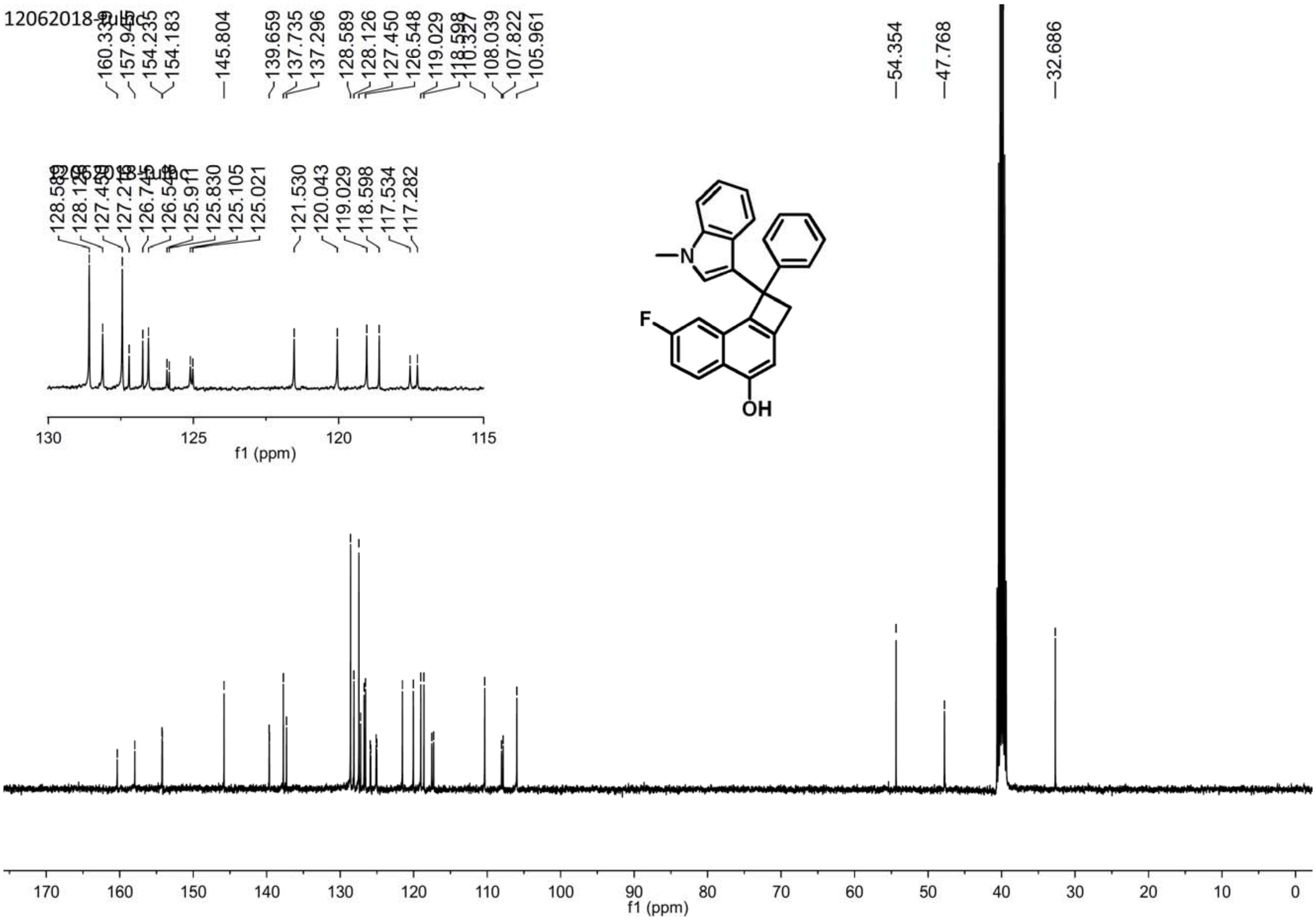

^{13}C NMR Spectrum of Compound 3s

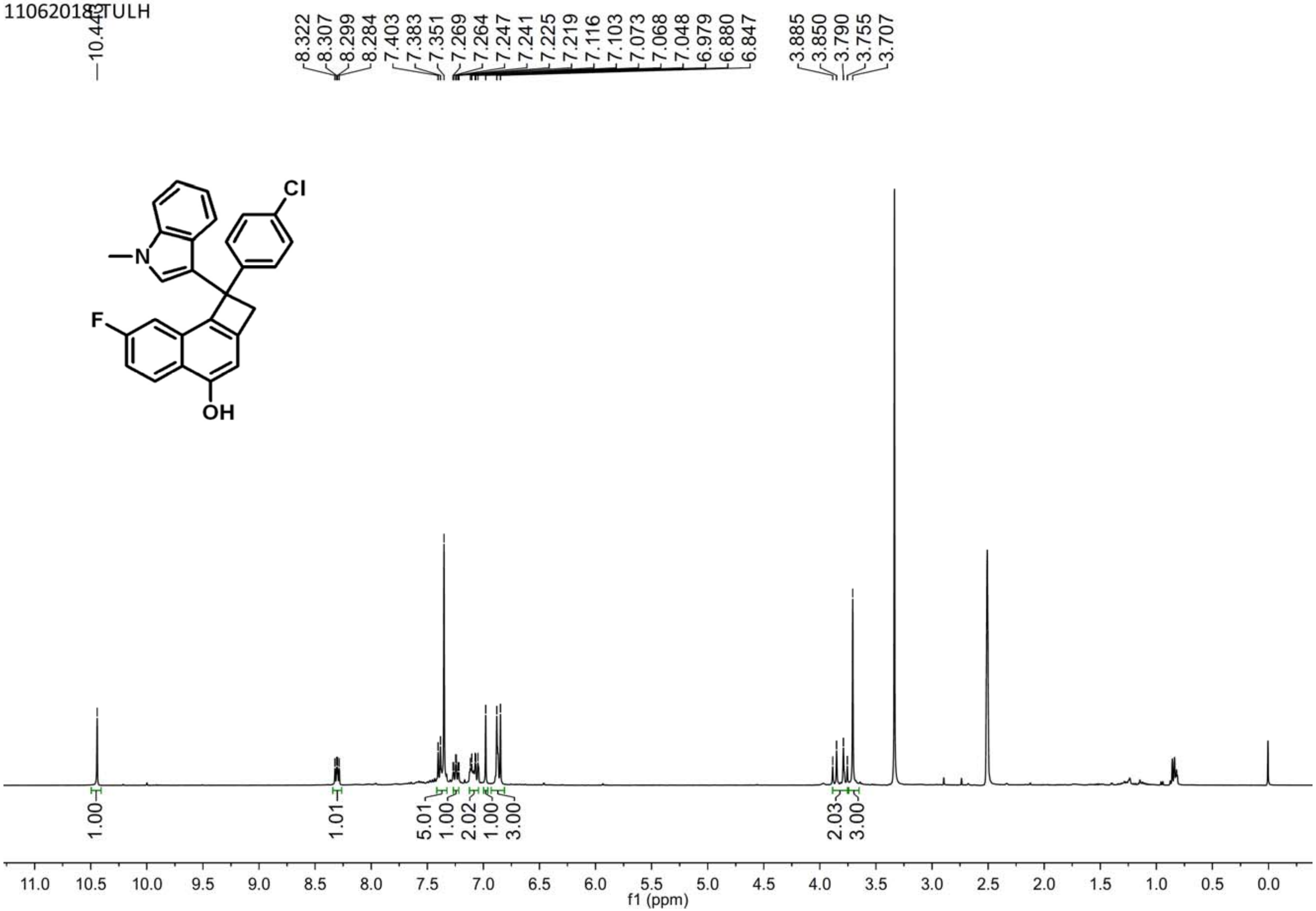

^{1}H NMR Spectrum of Compound 3t

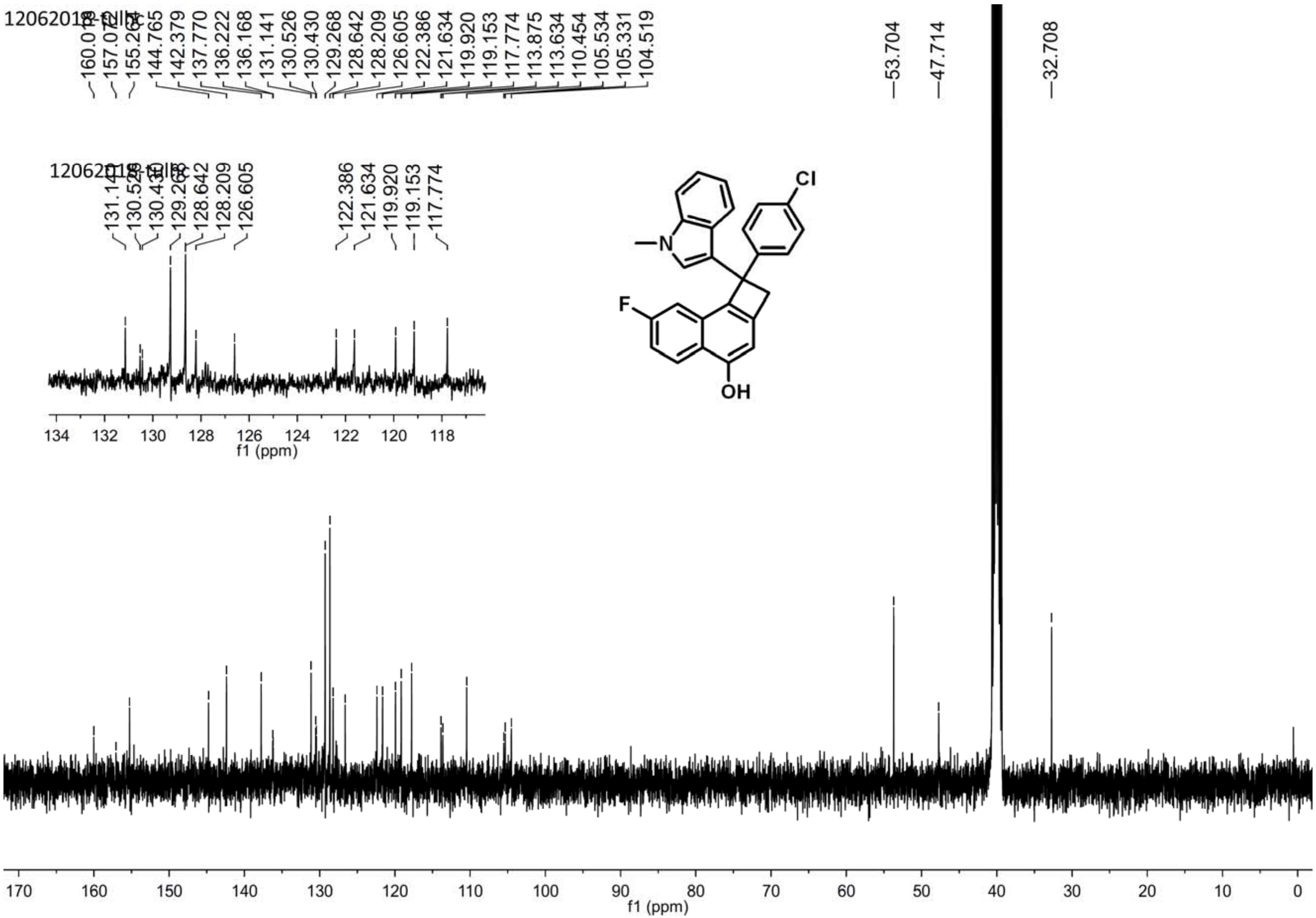

^{13}C NMR Spectrum of Compound 3t

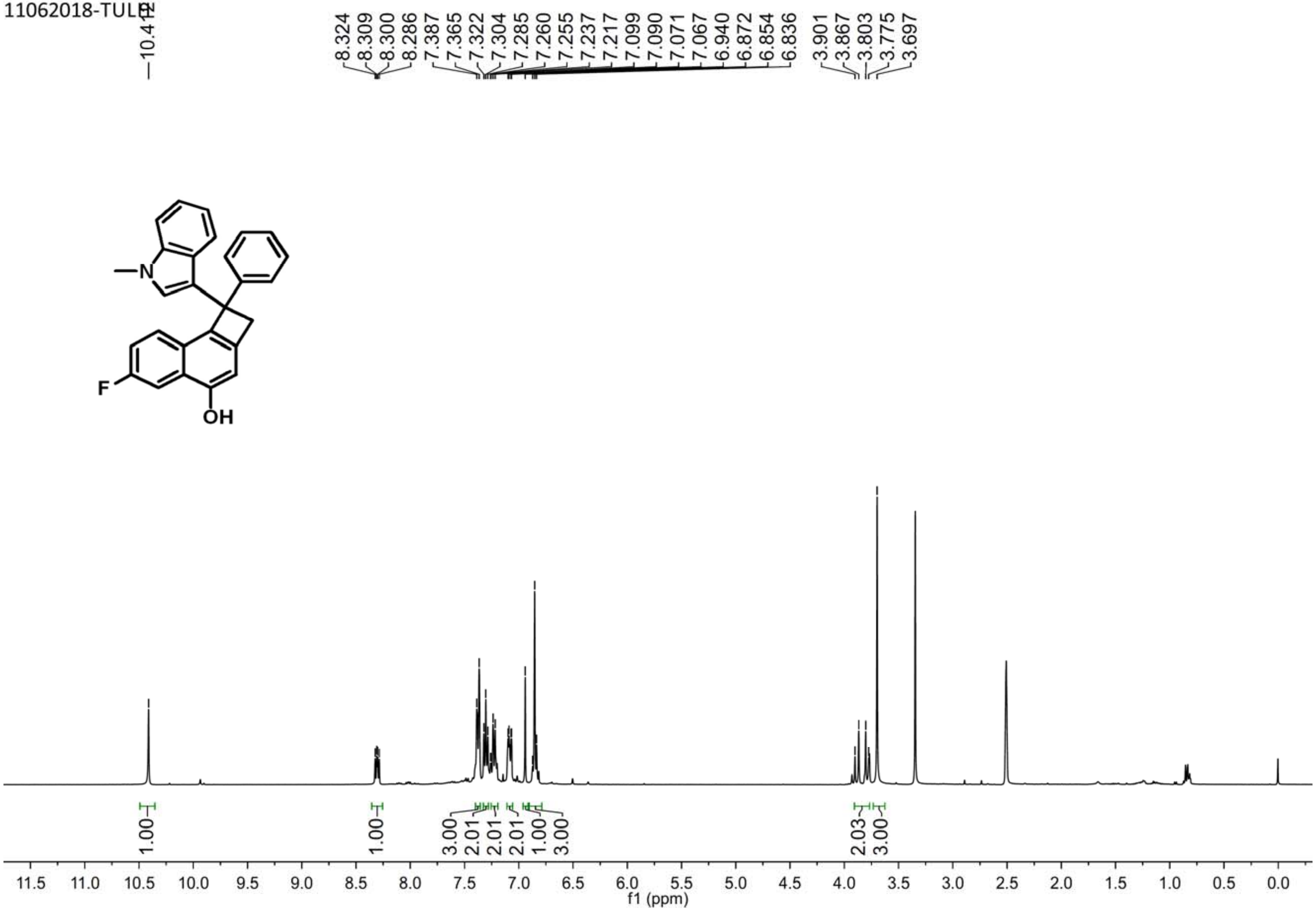

^{1}H NMR Spectrum of Compound 3u

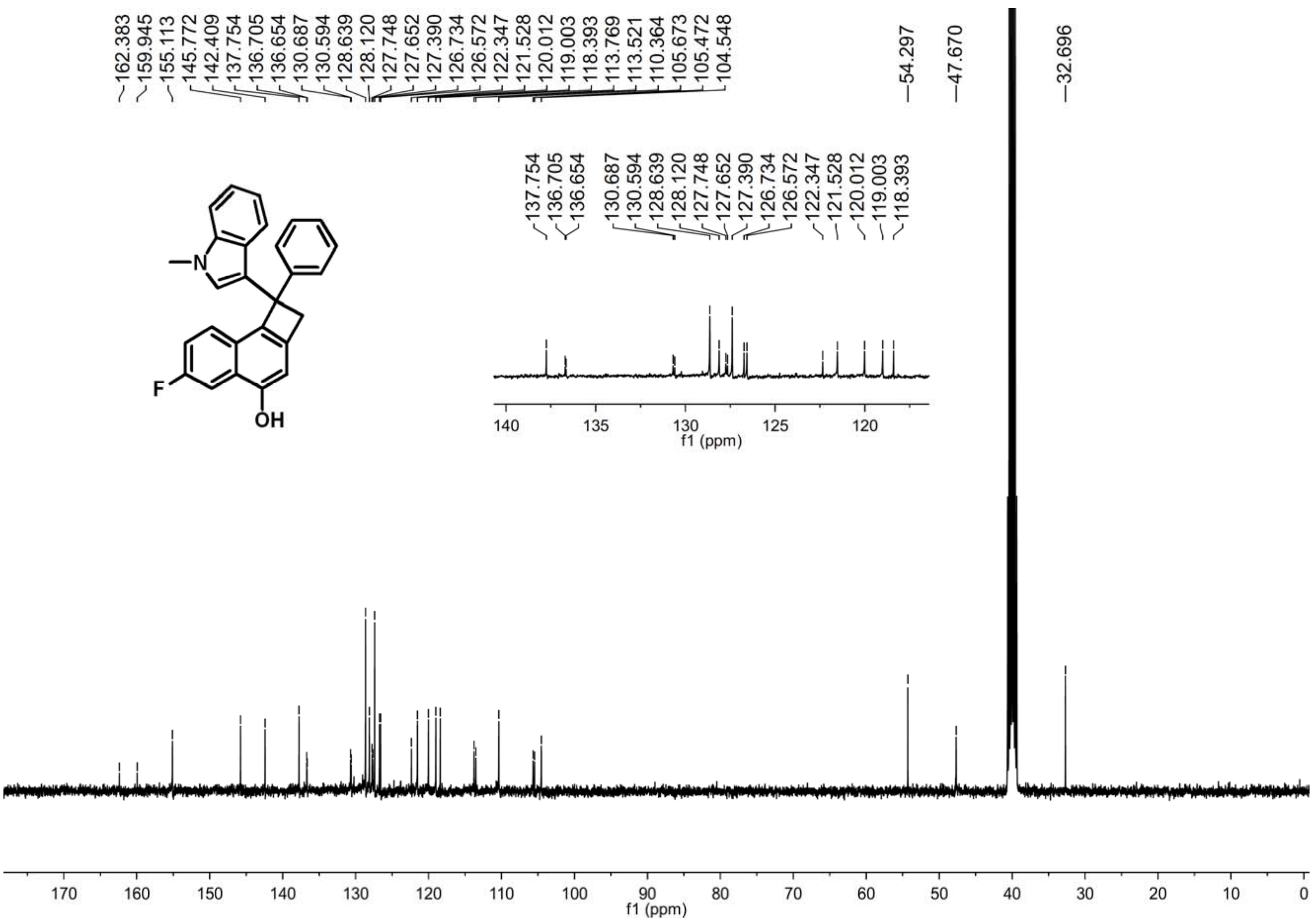

^{13}C NMR Spectrum of Compound 3u

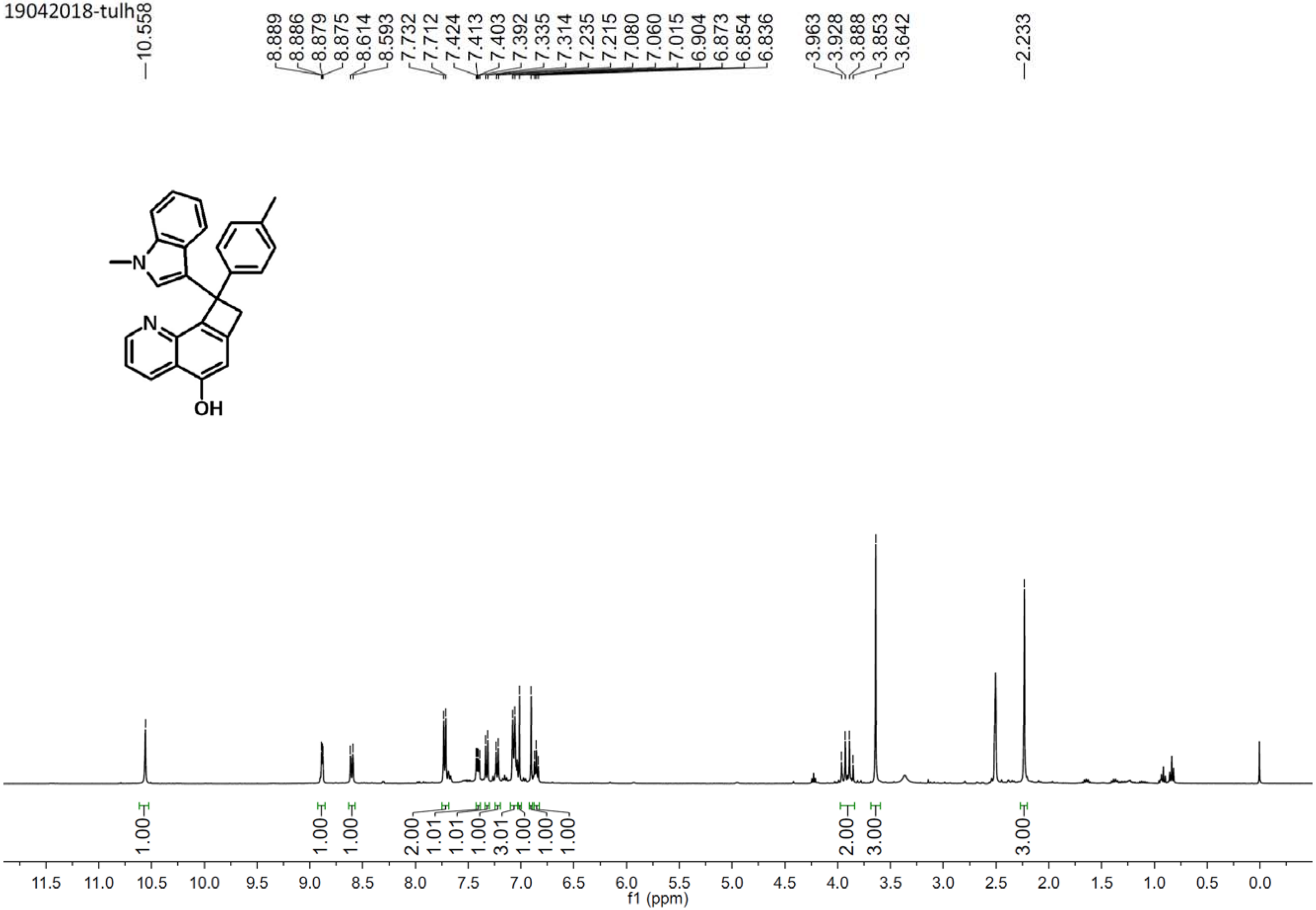

^{1}H NMR Spectrum of Compound 3v

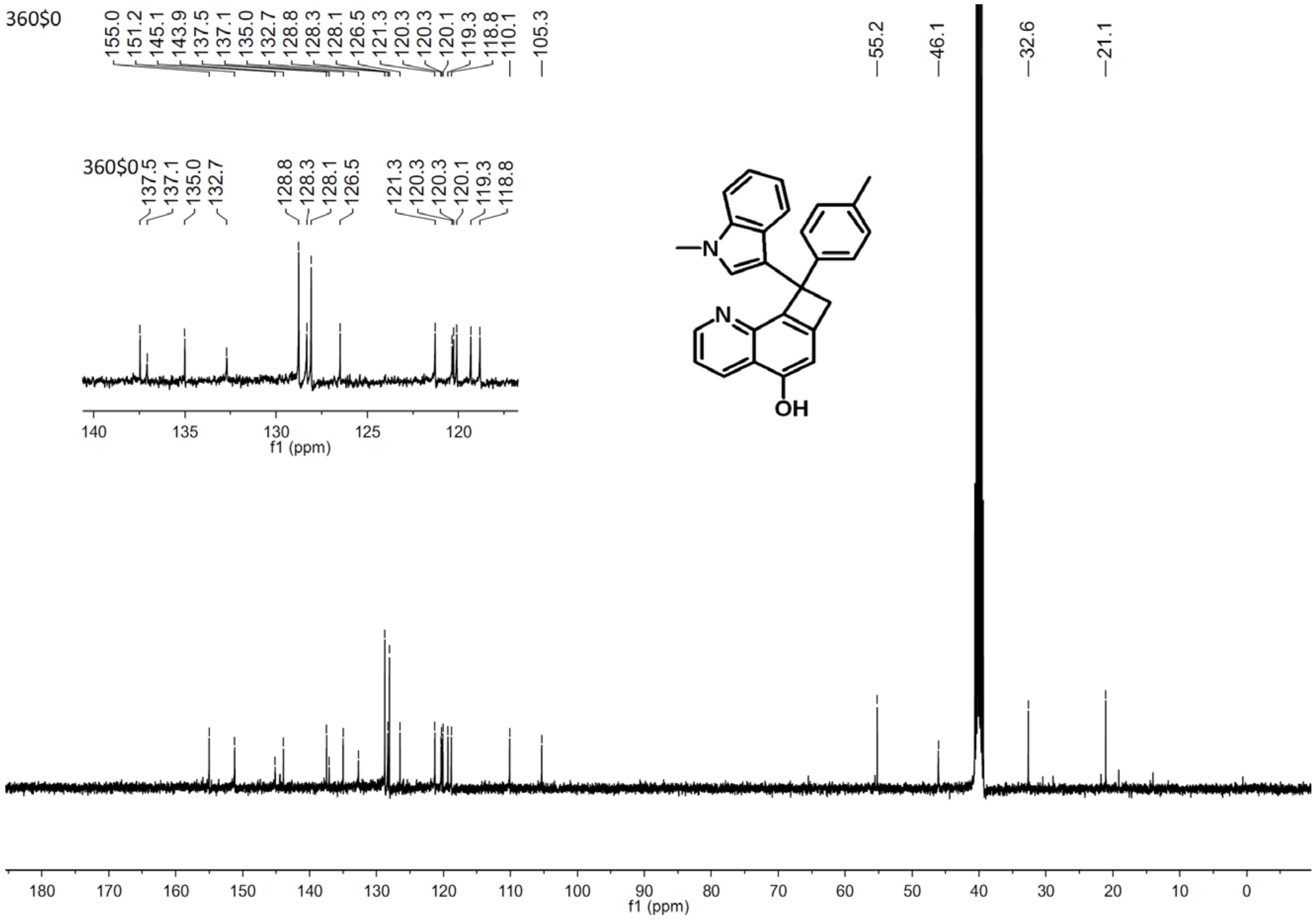

^{13}C NMR Spectrum of Compound 3v

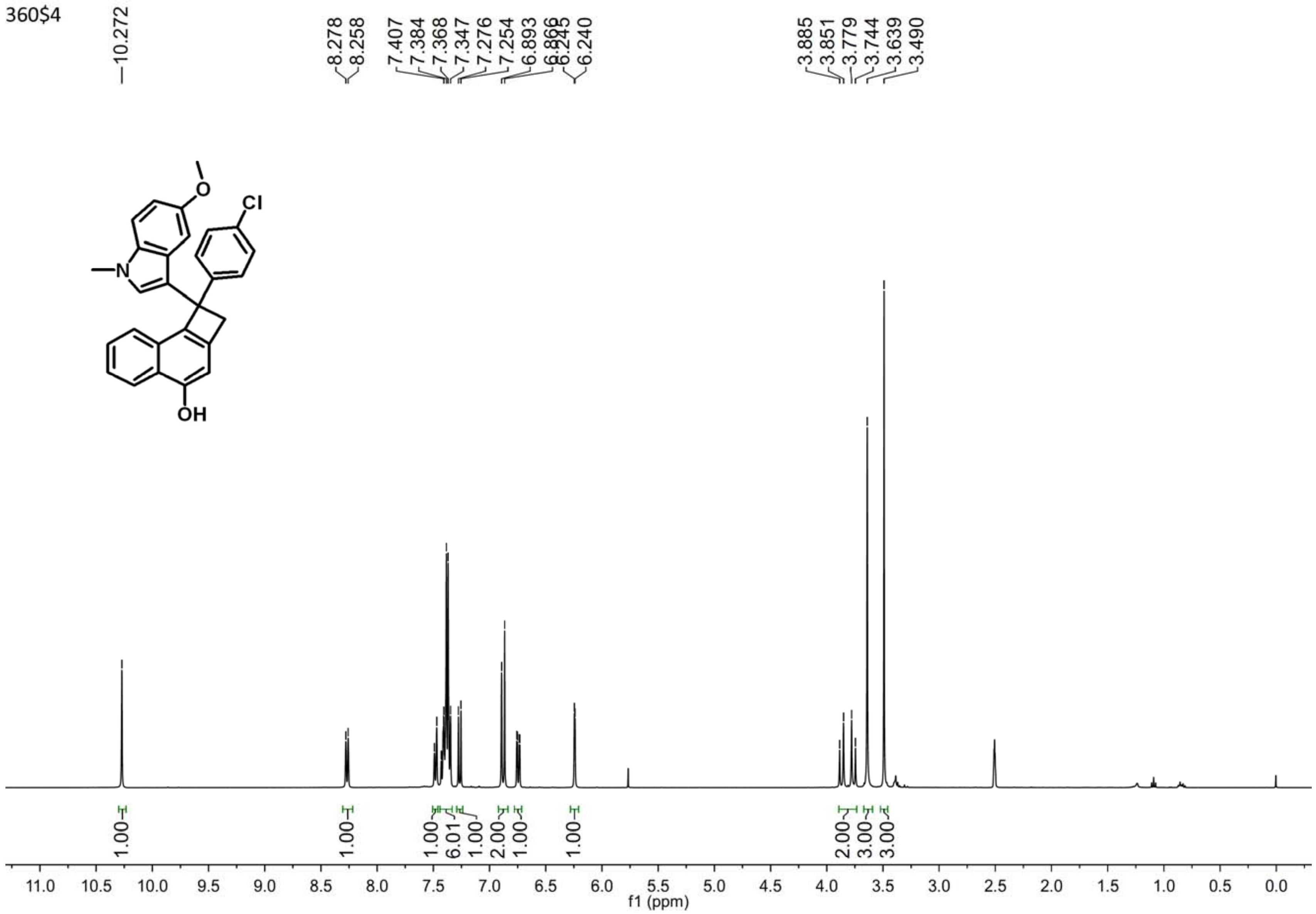

^{1}H NMR Spectrum of Compound 3w

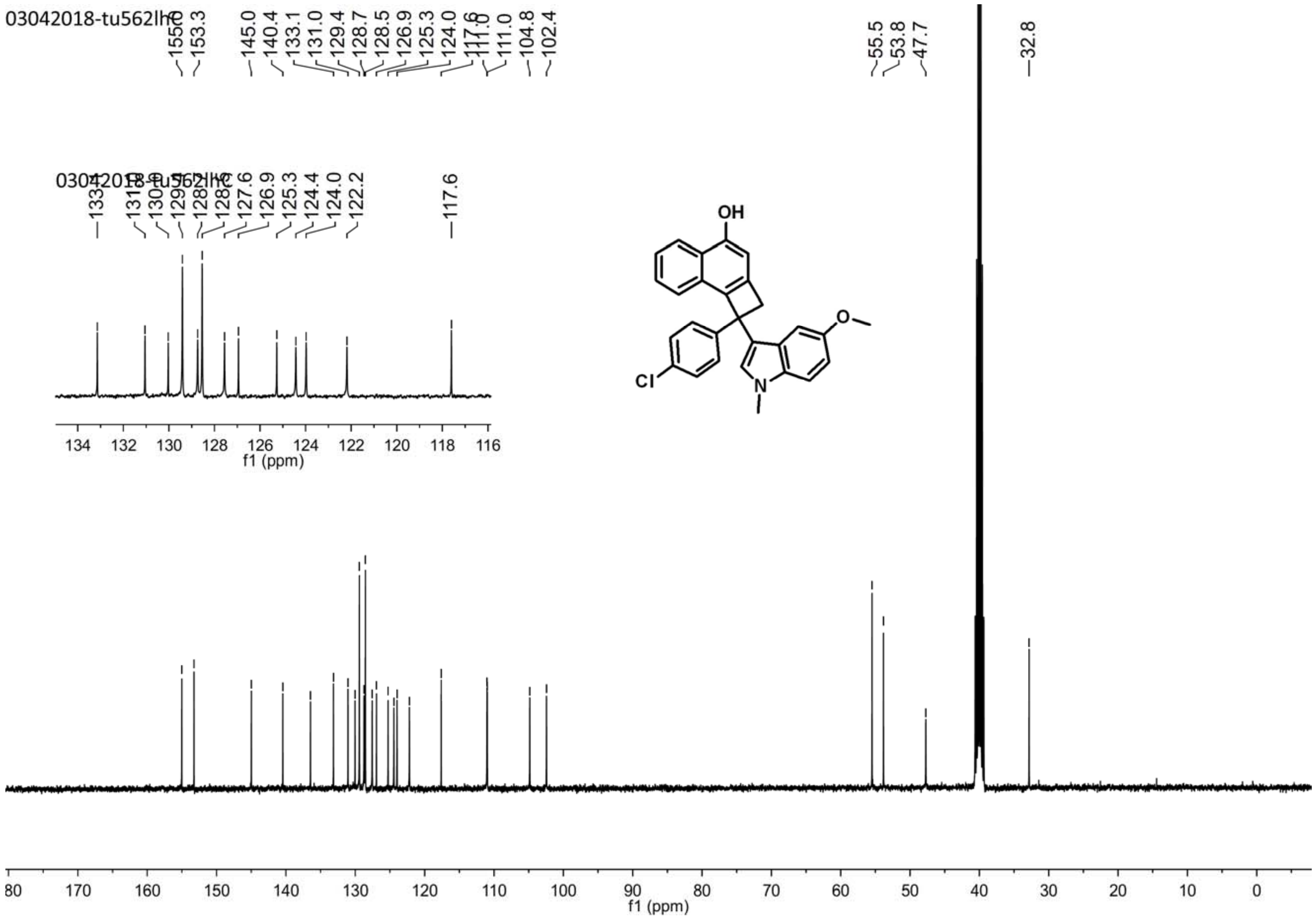

^{13}C NMR Spectrum of Compound 3w

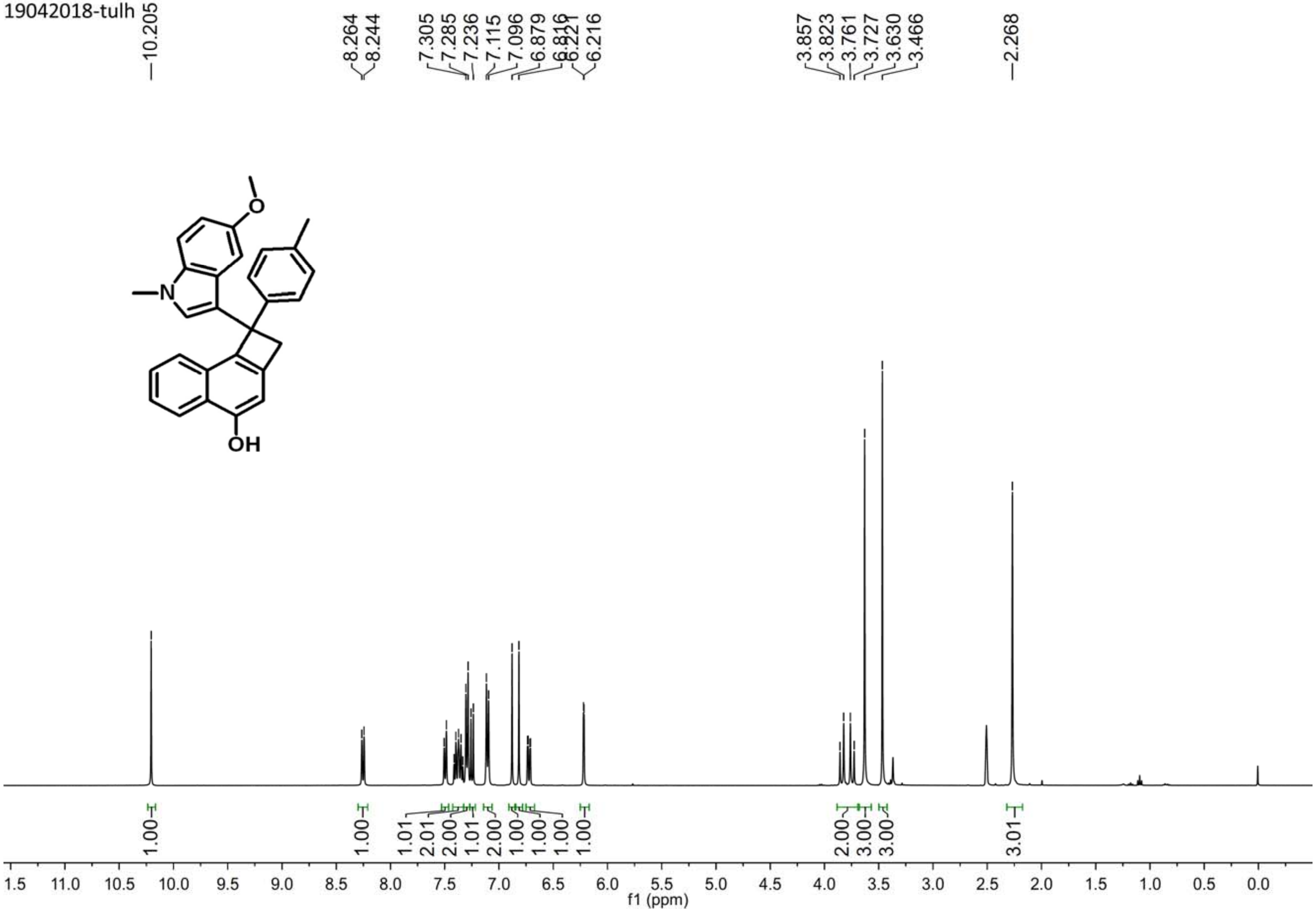

^{1}H NMR Spectrum of Compound 3x

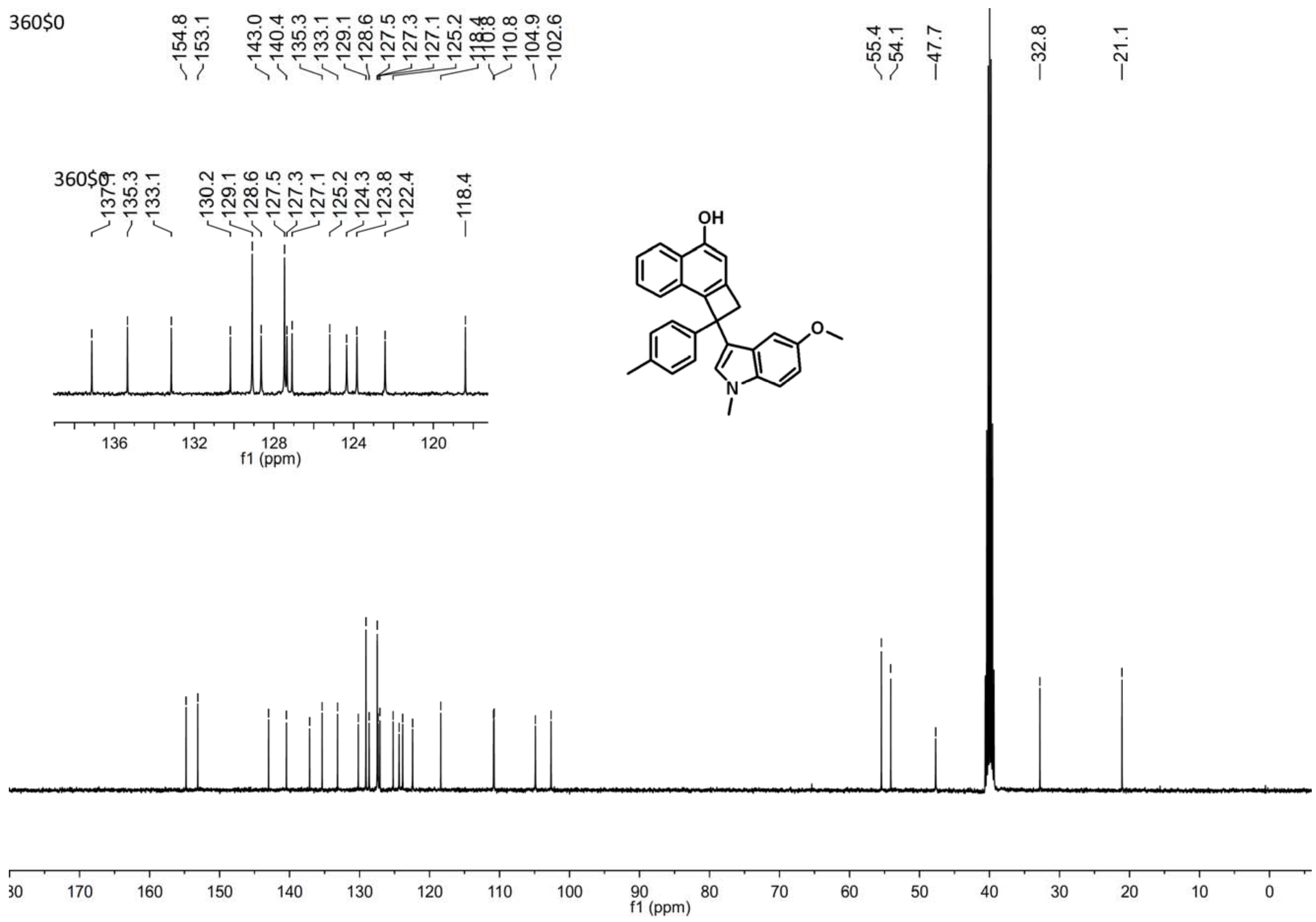

^{13}C NMR Spectrum of Compound 3x

360$4

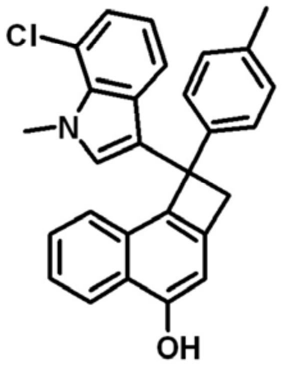

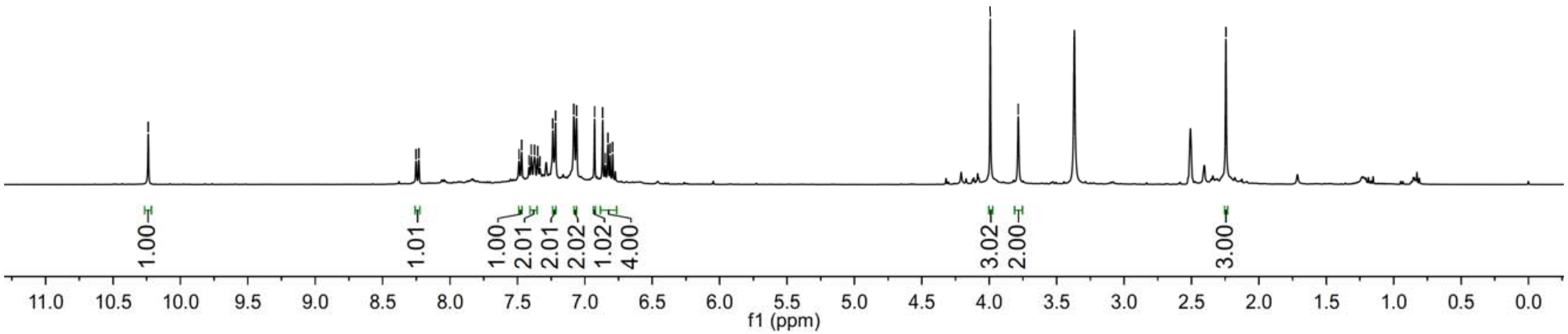

^{1}H NMR Spectrum of Compound 3y

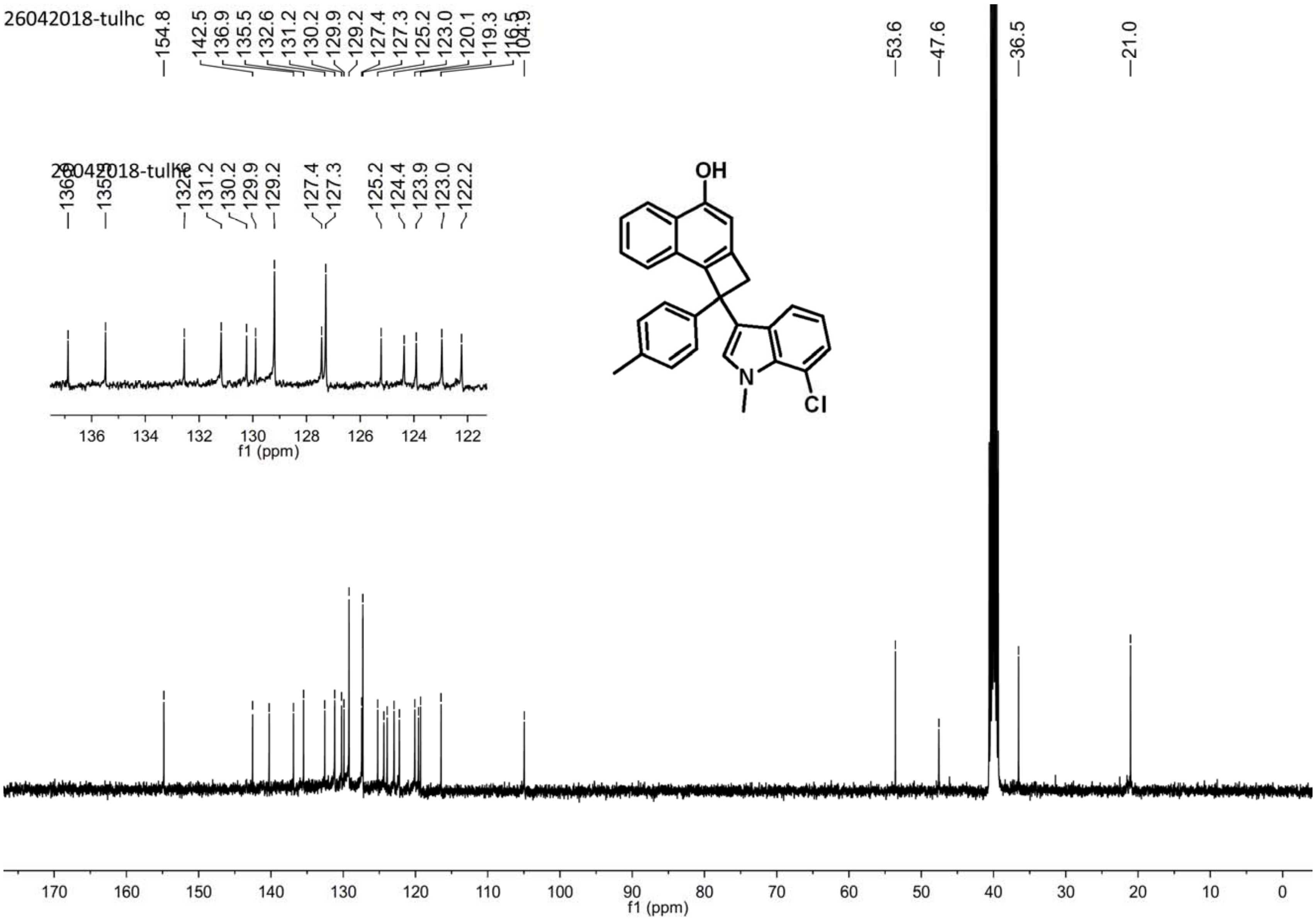

^{13}C NMR Spectrum of Compound 3y

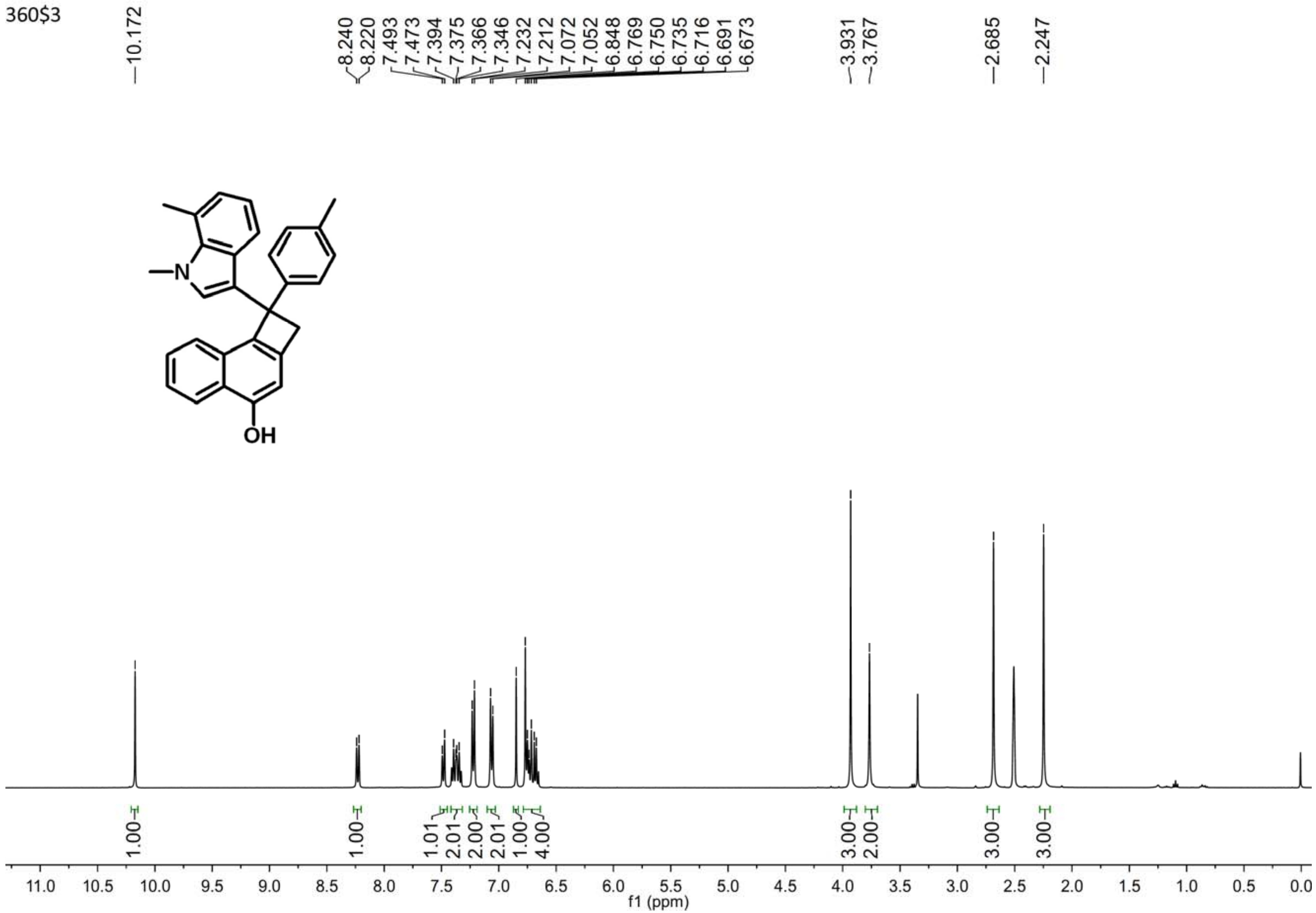

^{1}H NMR Spectrum of Compound 3z

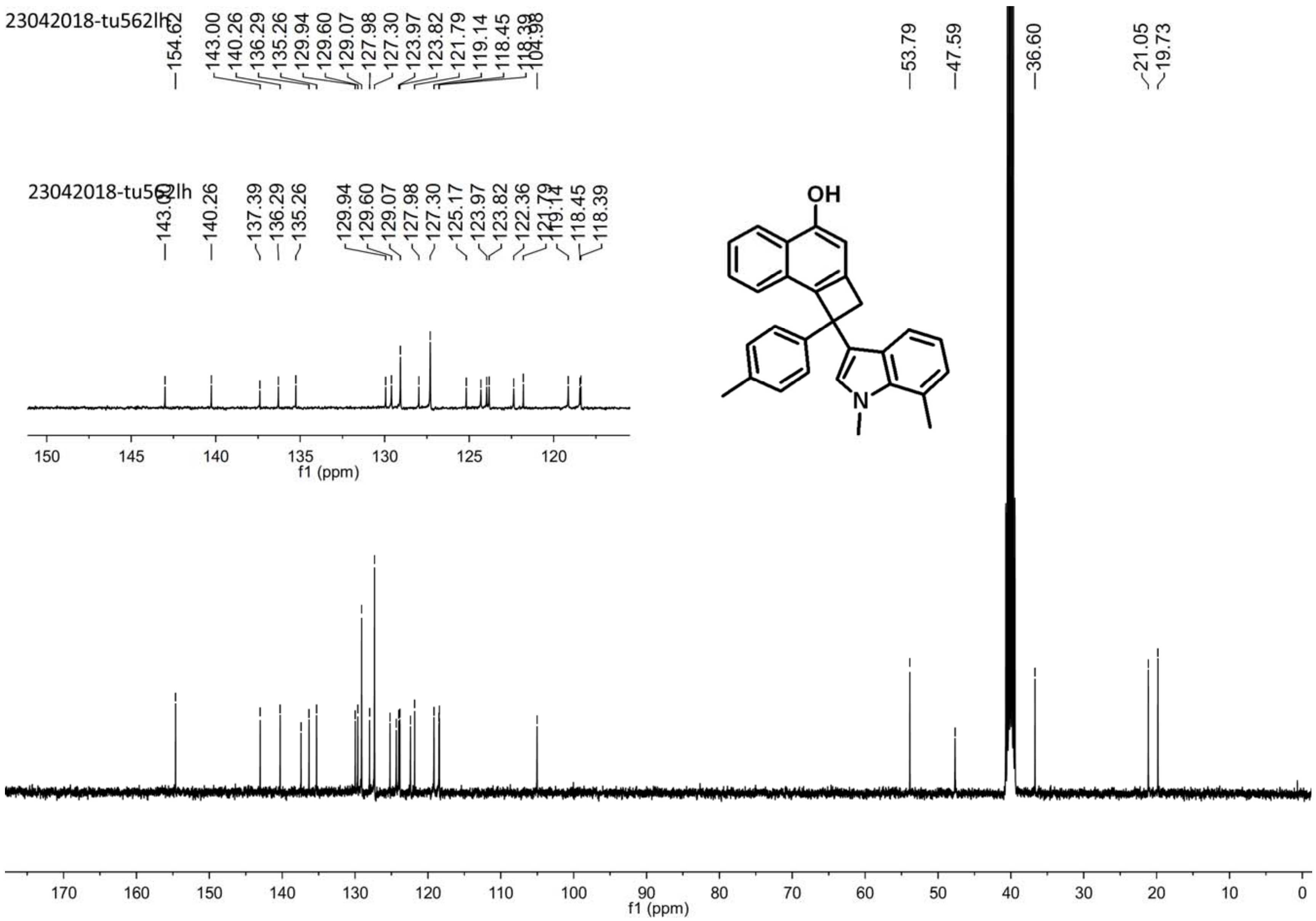

^{13}C NMR Spectrum of Compound 3z

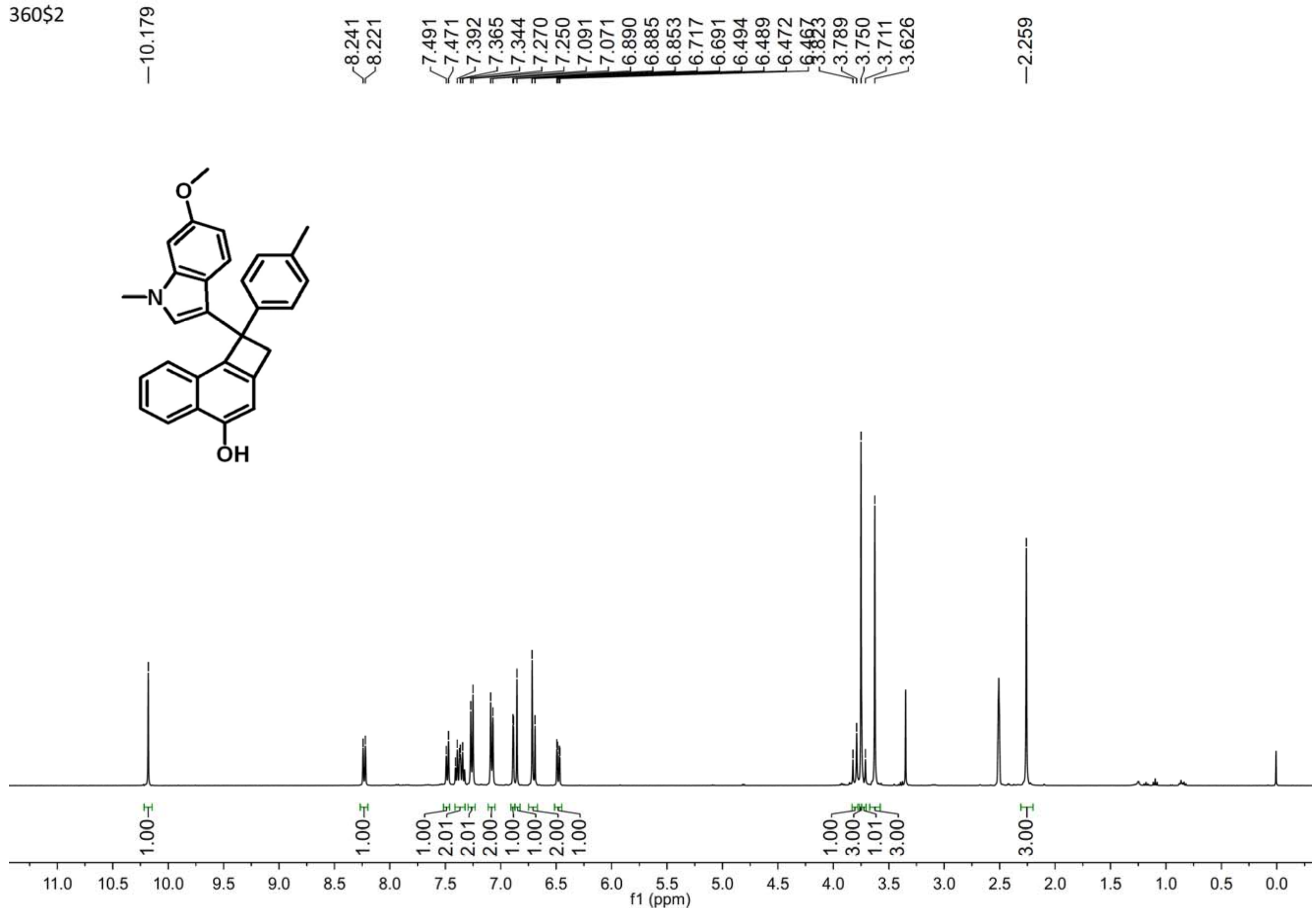

^{1}H NMR Spectrum of Compound 3aa

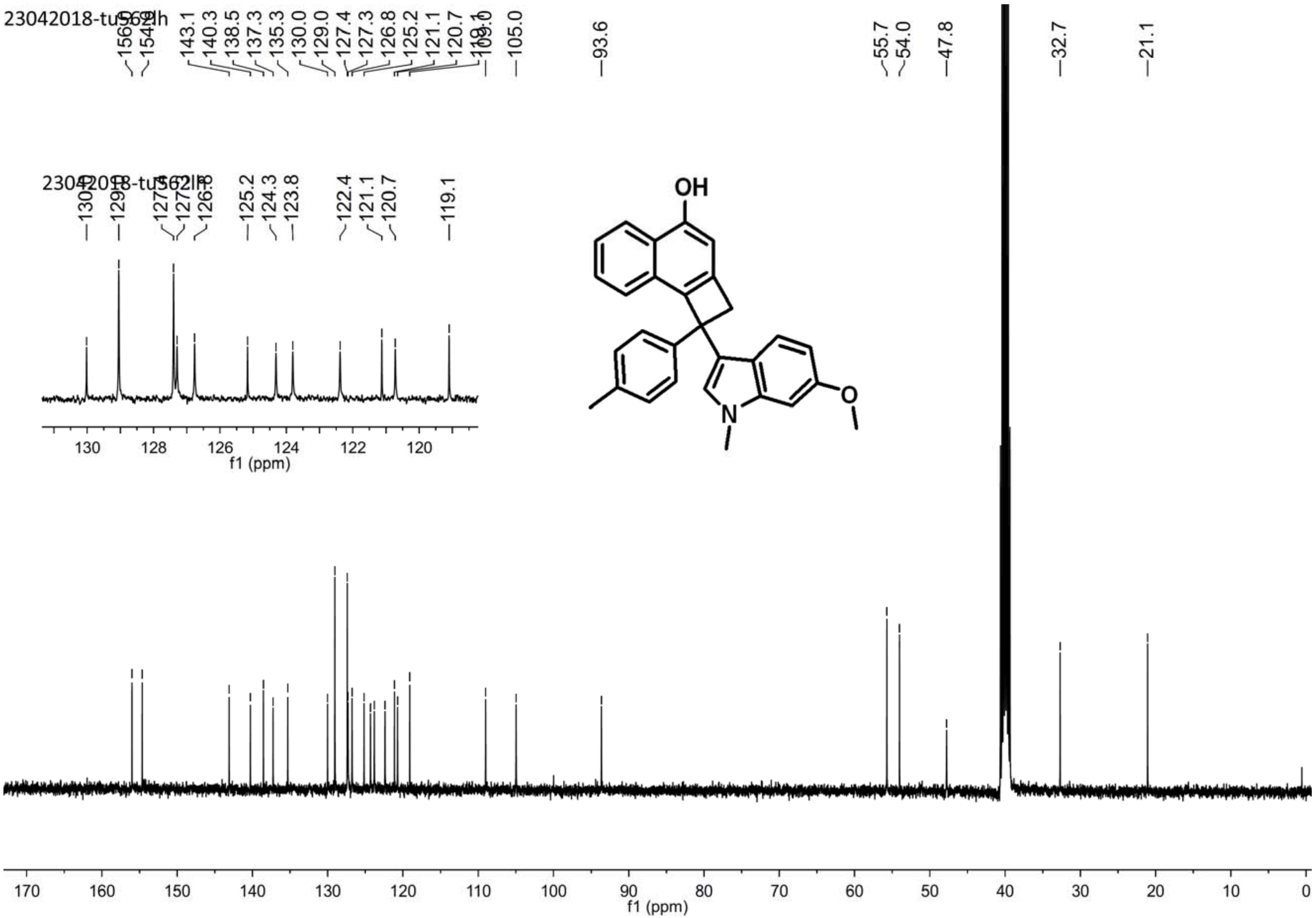

^{13}C NMR Spectrum of Compound 3aa

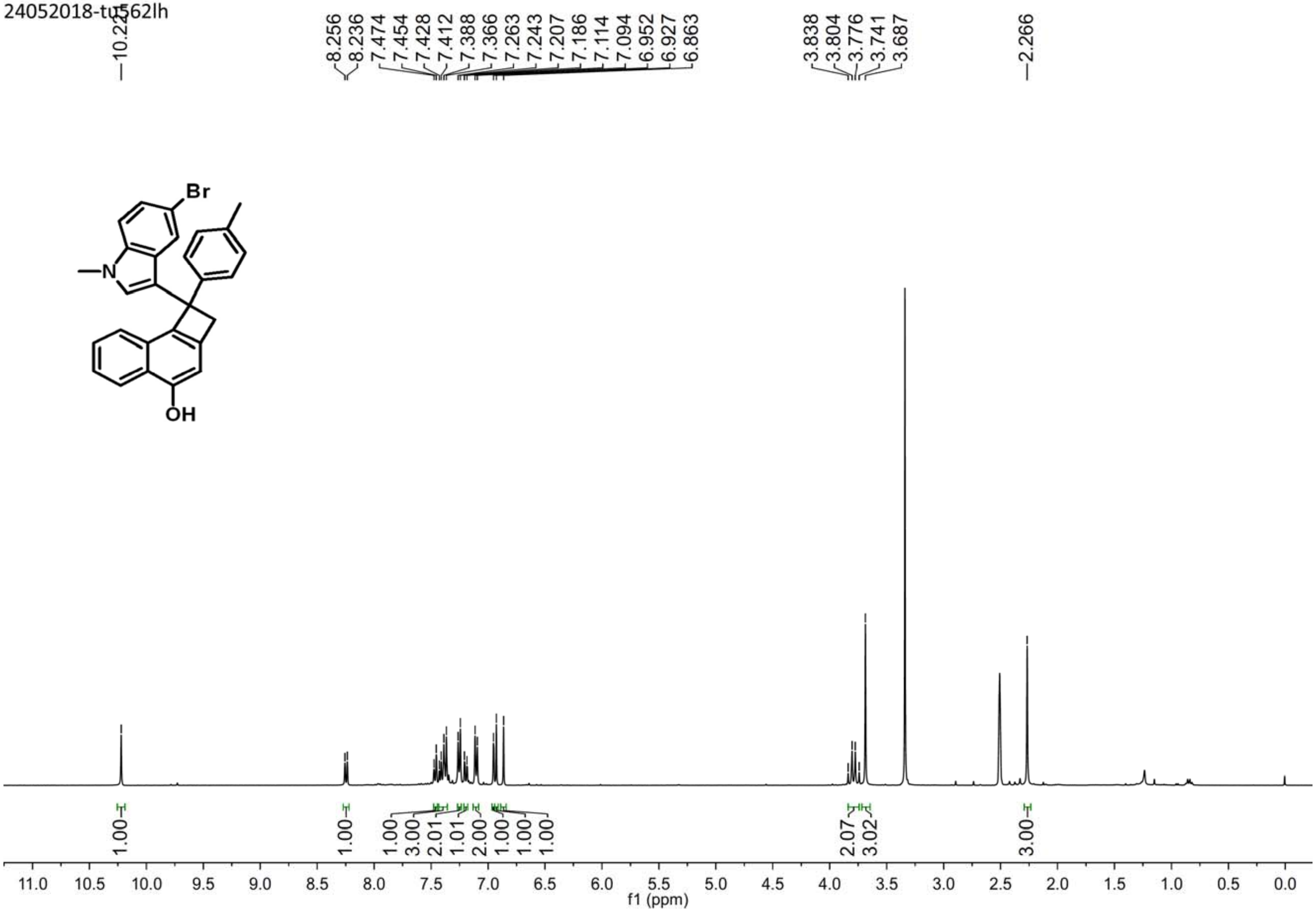

^{1}H NMR Spectrum of Compound 3bb

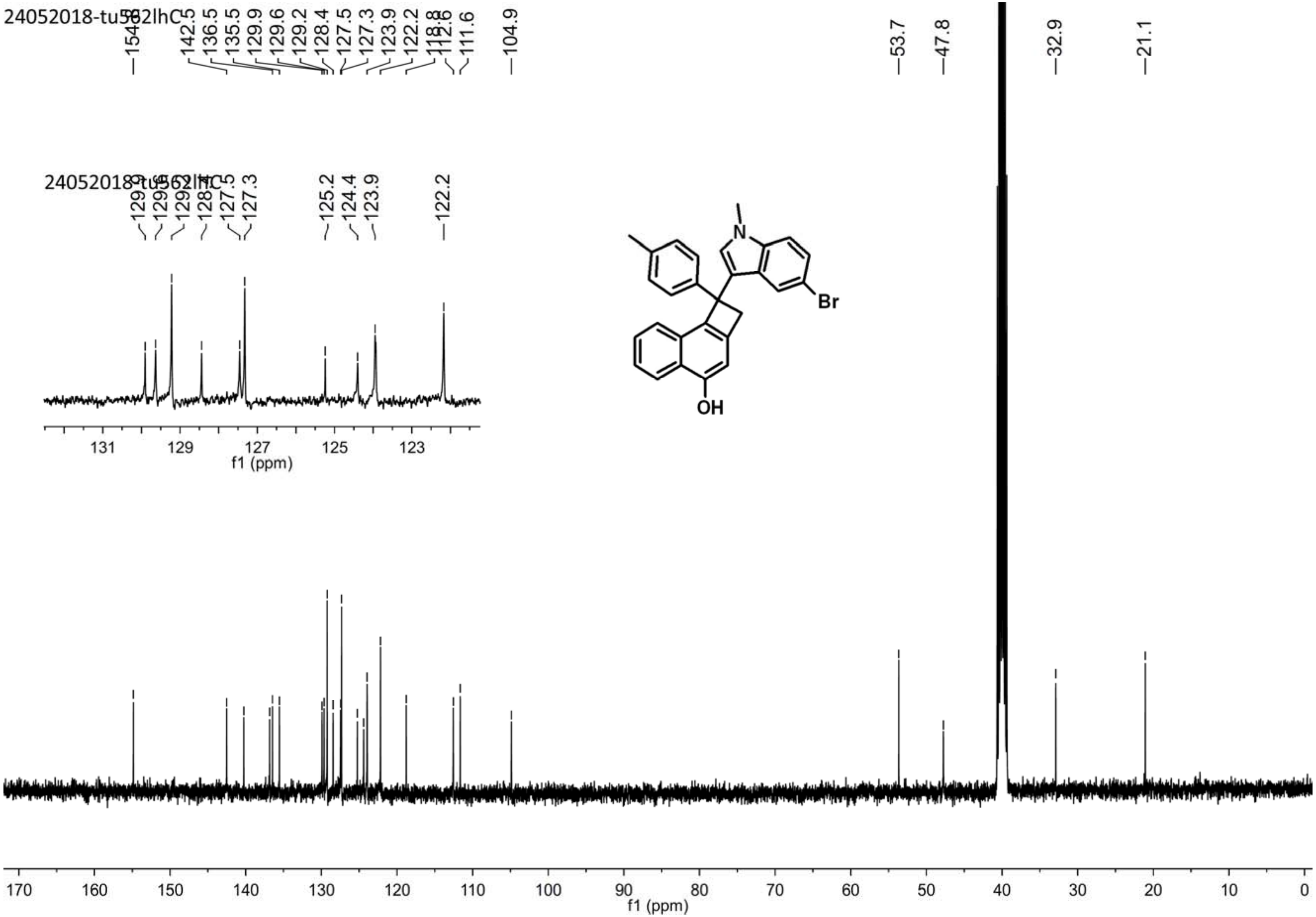

^{13}C NMR Spectrum of Compound 3bb

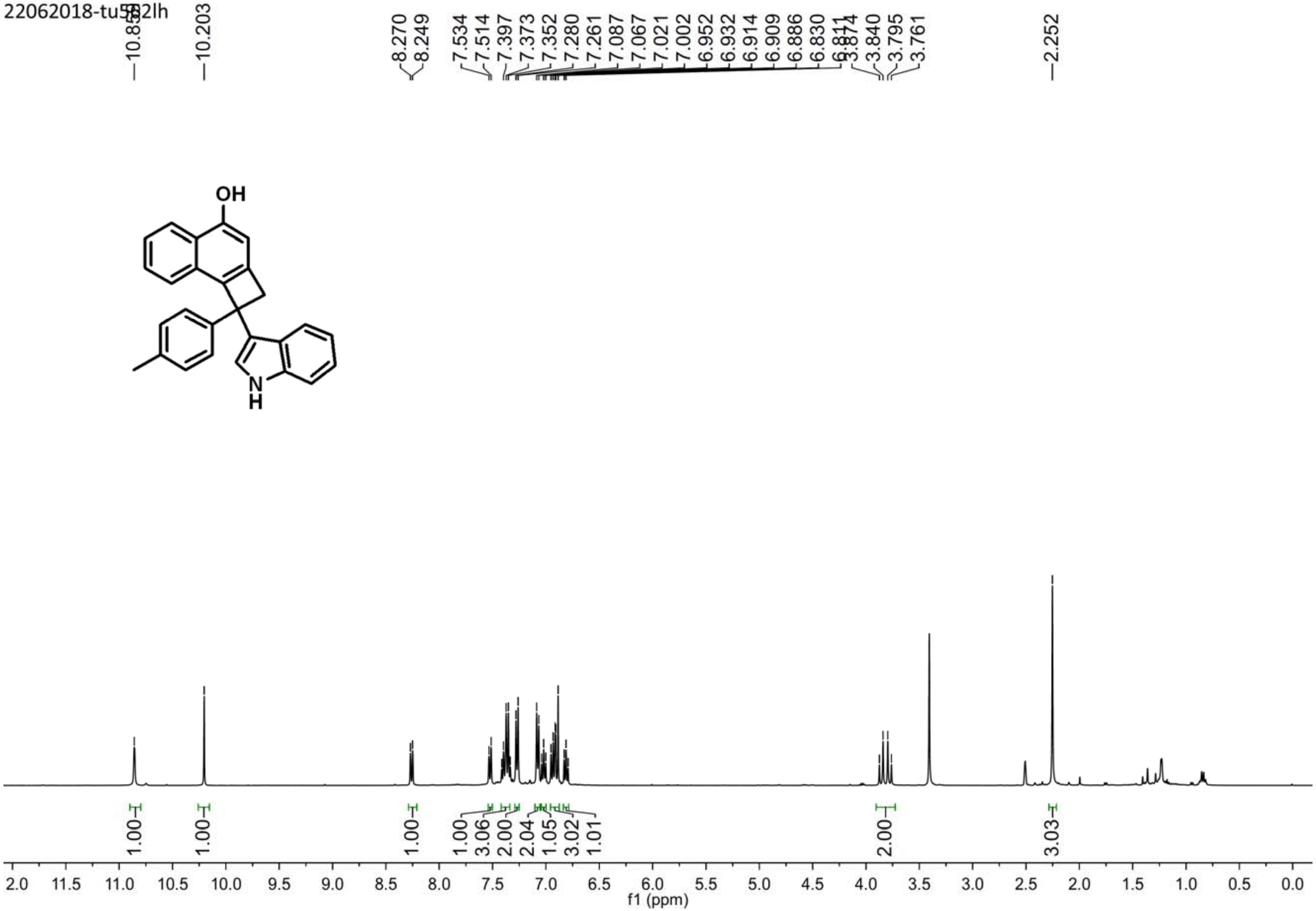

^{1}H NMR Spectrum of Compound 3cc

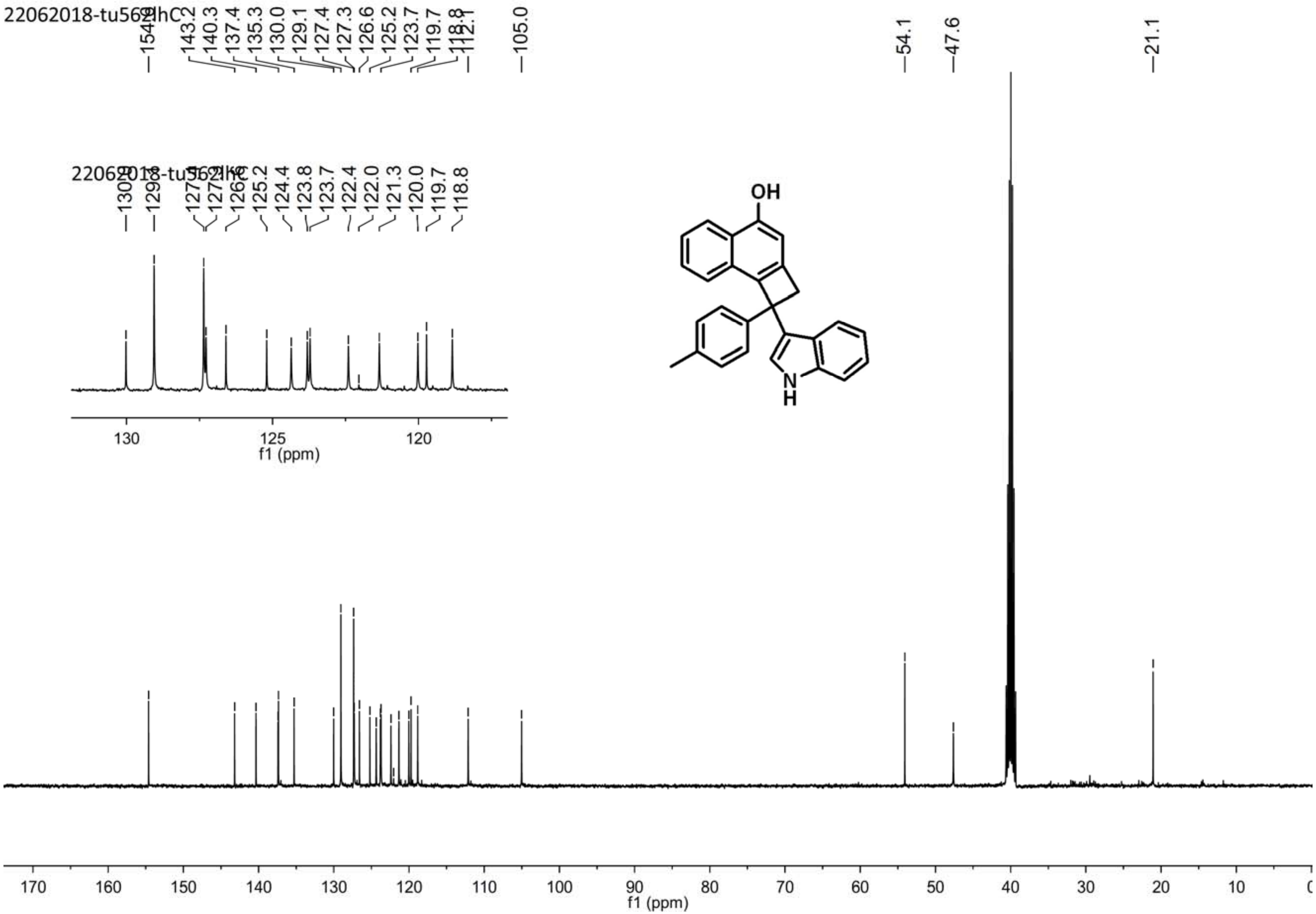

^{13}C NMR Spectrum of Compound 3cc

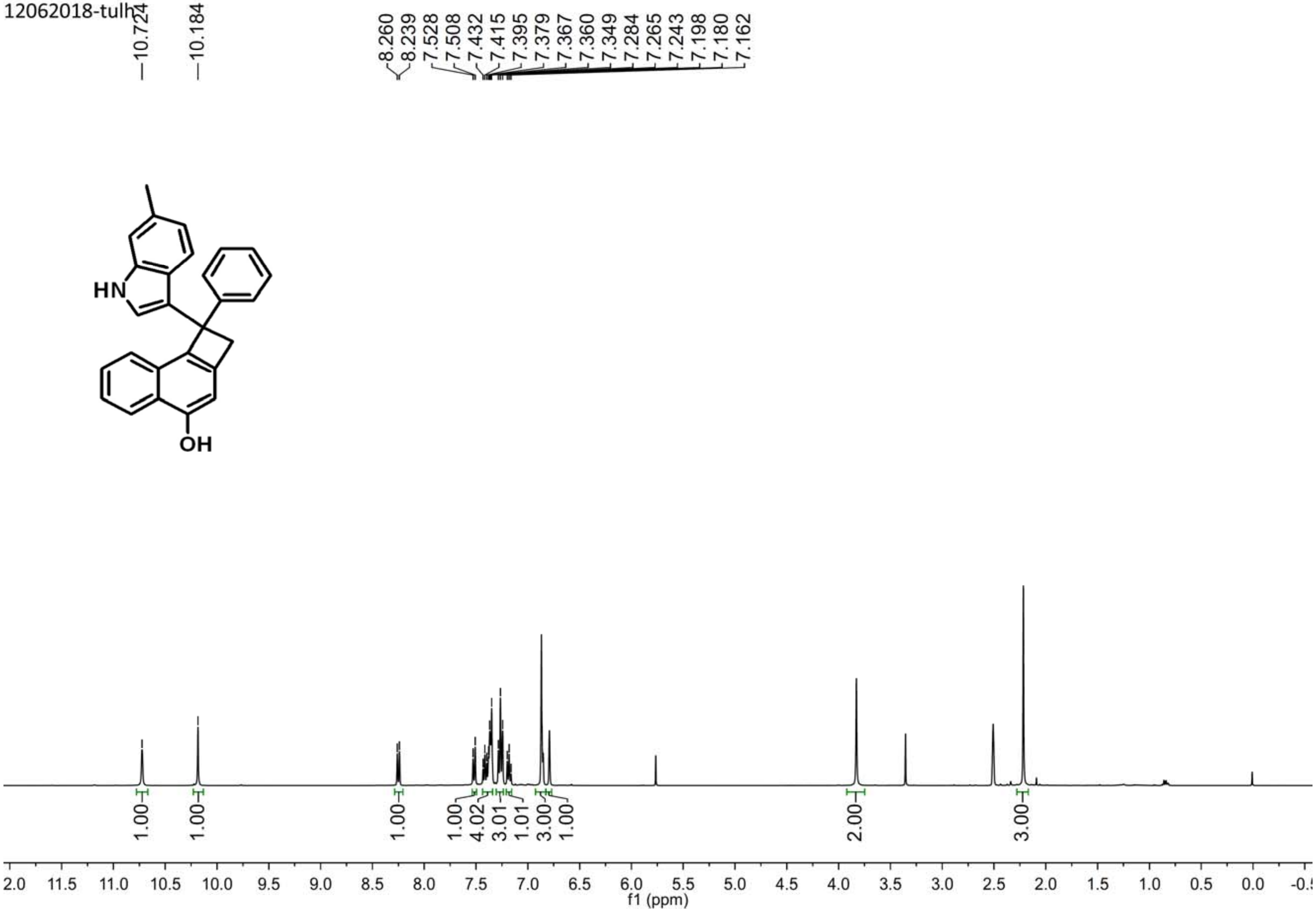

^{1}H NMR Spectrum of Compound 3dd

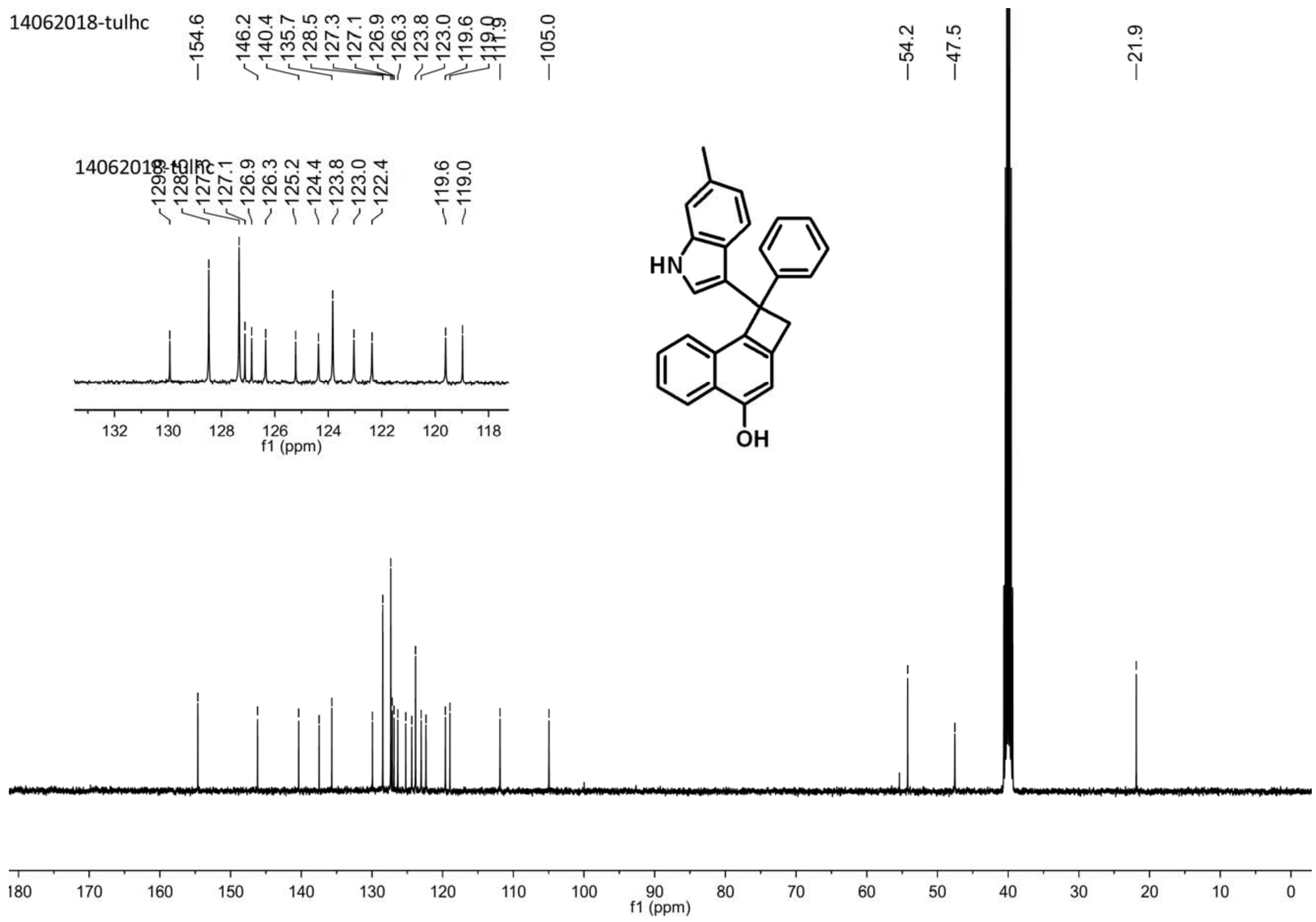

^{13}C NMR Spectrum of Compound 3dd
